# Tyrosinases: a family of copper-containing metalloenzymes

**DOI:** 10.1007/s40828-024-00195-y

**Published:** 2024-11-30

**Authors:** Matthias Pretzler, Annette Rompel

**Affiliations:** Institut für Biophysikalische Chemie, Fakultät für Chemie, Universität Wien, Josef-Holaubek-Platz 2, 1090 Vienna, Austria

**Keywords:** Oxygen activation, Copper enzyme, Polyphenol oxidase, Melanin, Mushroom tyrosinase, Protein expression

## Abstract

Tyrosinases (TYRs) are a family of copper-containing metalloenzymes that are present in all domains of life. TYRs catalyze the reactions that start the biosynthesis of melanin, the main pigment of the animal kingdom, and are also involved in the formation of the bright colors seen on the caps of mushrooms and in the petals of flowers. TYRs catalyze the *ortho*-hydroxylation and oxidation of phenols and the oxidation of catechols to the respective *o*-quinones. They only need molecular oxygen to do that, and the products of TYRs—*o*-quinones—are highly reactive and will usually react with the next available nucleophile. This reactivity can be harnessed for pharmaceutical applications as well as in environmental and food biotechnology. The majority of both basic and applied research on TYRs utilizes “mushroom tyrosinase”, a crude enzyme preparation derived from button mushroom (*Agaricus bisporus*) fruiting bodies. Access to pure TYR preparations comes almost exclusively from the production of recombinant TYRs as the purification of these enzymes from the natural source is usually very laborious and plagued by low yields. In this text an introduction into the biochemistry of the enzyme TYR will be given, followed by an overview of available structural data of TYRs, the current model for the catalytic mechanism, a survey of reports on the recombinant production of this important metalloenzyme family, and a review of the applications of TYRs for the synthesis of catechols, as biosensors, in bioremediation, for the cross-linking of proteins and medical hydrogels as well as for melanoma treatment.

**Graphical Abstract:**

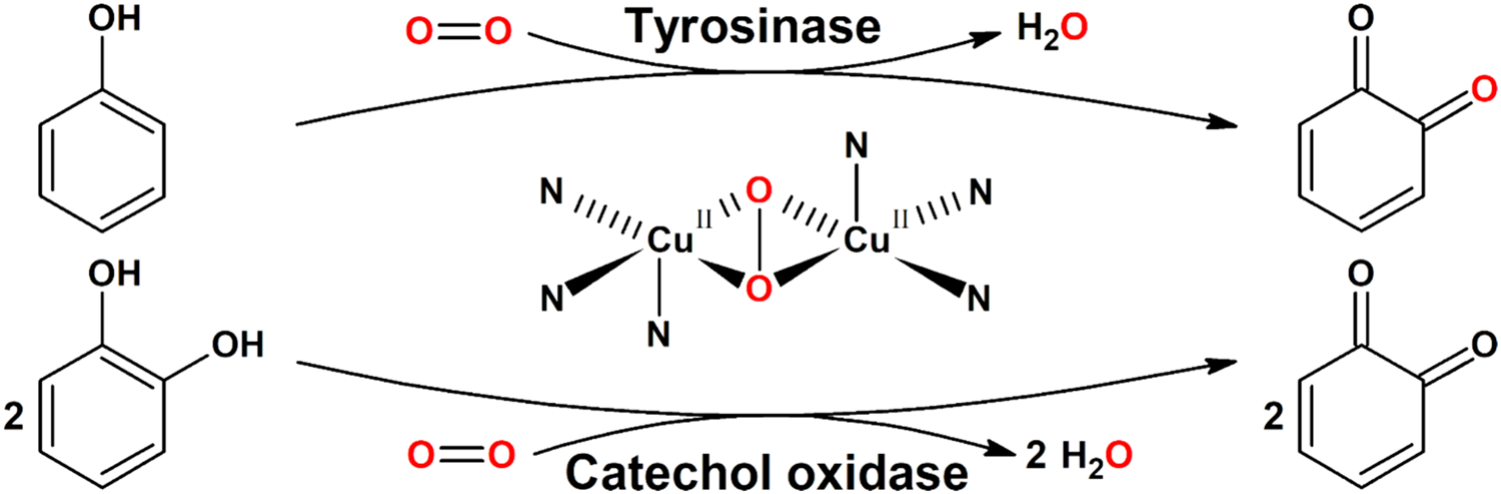

## Tyrosinase

**Scheme 1:**
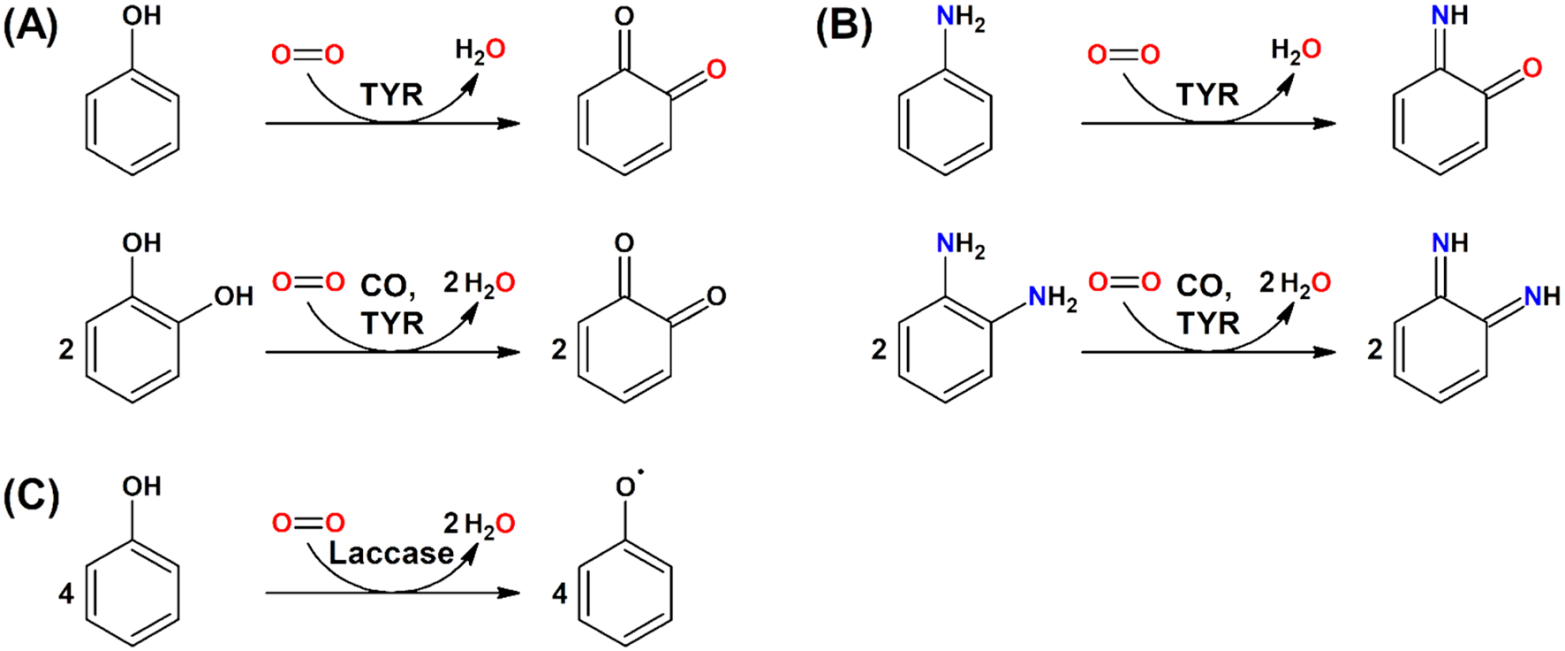
Reactions catalyzed by TYR (EC 1.14.18.1), catechol oxidase (CO, EC 1.10.3.1), and laccase (EC 1.10.3.2). **A** Main enzymatic activity of PPOs: *ortho*-hydroxylation and oxidation of phenols (by TYRs) or only oxidation of catechols (for COs) to the corresponding *o*-quinones [15]. **B** Promiscuous enzymatic activity: hydroxylation and oxidation of aromatic amines (anilines) to *o*-benzoquinone imines [16] and oxidation of vicinal aromatic diamines to *o*-benzoquinone diimines. **C** Laccases oxidize their substrates by single electron abstraction, four electrons and four protons are stored in the enzyme and are used for the reduction of oxygen to water

**Scheme 2:**
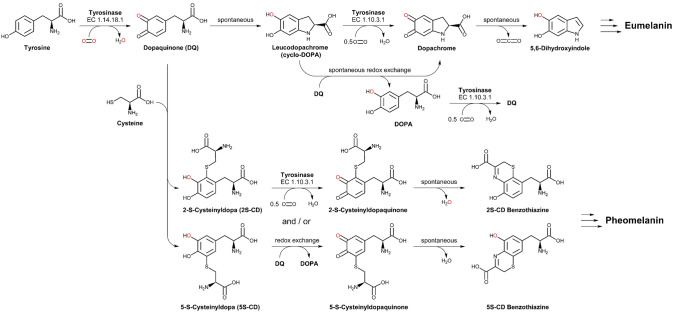
The initial steps in the biosynthesis of melanin [17]. l-Tyrosine is oxidized by molecular oxygen via tyrosinase, yielding *ortho*-dopaquinone and water. This quinone is quite reactive and reacts rapidly with strong nucleophiles like cysteine in a Michael addition reaction (*r* = 3 × 10^7^ M^−1^ s^−1^) [17]. In the absence of strong nucleophiles, however, dopaquinone undergoes spontaneous cyclization (*r* = 7.6 s^−1^) [17] to leucodopachrome, which is again oxidized by either tyrosinase yielding dopachrome or via redox exchange with dopaquinone (*r* < 4 × 10^7^ M^−1^ s^−1^) [17] leading to one molecule of DOPA and dopachrome each. Dopachrome then decomposes in solution, yielding mostly 5,6-dihydroxyindole and a lower amount of 5,6-dihydroxyindole-2-carboxylic acid. Eumelanin is then formed by oxidation of these dihydroxyindoles. Pheomelanin is formed in the presence of cysteine, which reacts rapidly with dopaquinone to 5-*S*-cysteinyldopa and (to a lesser extent) 2-*S*-cysteinyldopa. Those cysteinyldopas are again oxidized by tyrosinase or via redox exchange with dopaquinone (*r* = 8.8 × 10^5^ M^−1^ s^−1^) [17] and the resulting cysteinyldopaquinones react further to benzothiazine intermediates and finally yield pheomelanin

**Scheme 3:**
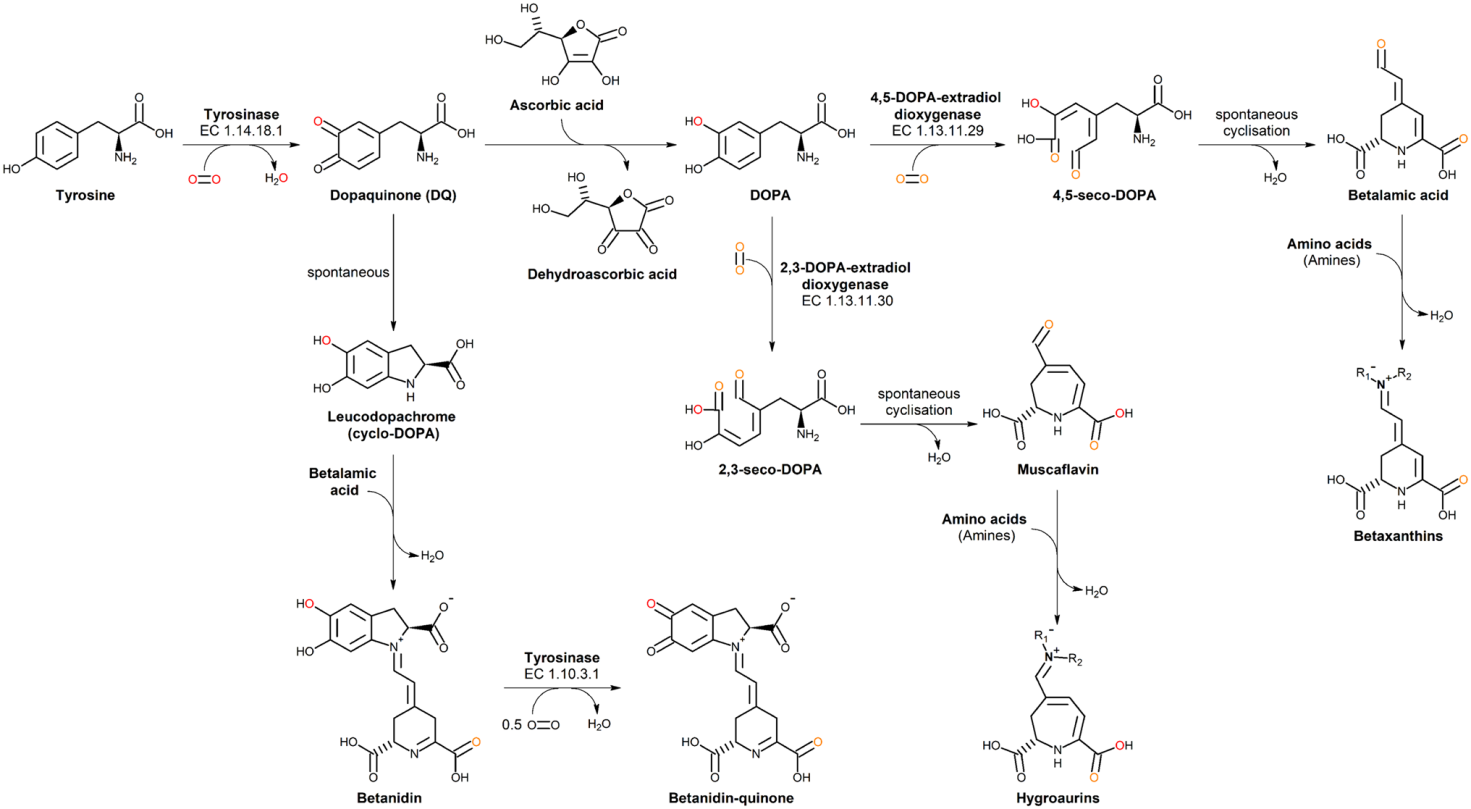
Tyrosinase involvement in the biosynthesis of betalain pigments [18]. The *o*-dopaquinone formed by tyrosinase-mediated oxidation of l-tyrosine is reduced to the catechol l-DOPA by reduction with ascorbic acid or by spontaneous cyclization yielding leucodopachrome (cylo-DOPA). The catecholic ring of l-DOPA is opened via an oxidation introducing both oxygen atoms of molecular oxygen into the molecule that is catalyzed by the metalloenzyme DOPA-extradiol oxygenase [19]. Oxidative cleavage between the 4th and 5th carbon atoms in the aromatic ring leads to betalamic acid, the key intermediate of betalain biosynthesis [18]. A fungal DOPA-extradiol dioxygenase has been shown to possess both 4,5- and 2,3-DOPA-extradiol dioxygenase activity [20]. The latter gives rise to the formation of muscaflavin, which is able to react with amino acids to hygroaurin pigments [21] in a manner analogous to the formation of betanidin and betaxanthins from betalamic acid [18]. R_1_ and R_2_ refer to generic amino acid or amine side chains involved in the formation of hygroaurin and betaxanthin pigments

Tyrosinases (TYRs) are a ubiquitous family of metalloenzymes that is found in all domains of life [1]. They are of central importance for the pigmentation in vertebrates as the oxygenation of l-tyrosine catalyzed by tyrosinase provides the starting material for melanin biosynthesis [2] (Schemes 1 and 2). Melanins are water-insoluble [3] and represent the main pigment in the hair, eyes, and skin of mammals [4]. They provide protection against UV irradiation, are needed for the sclerotization of insect exoskeletons [5], and are the end products of enzymatic browning of fruits and vegetables [6]. TYRs are also involved in the formation of the water-soluble red and yellow betalain pigments (Scheme 3) that, e.g., provide the characteristic red color of fly agaric (*Amanita muscaria*) caps and take part in the formation of color in the petals of ornamental plants [7, 8].

Oxygen-activating agents in mushrooms have been known in the scientific literature since 1855, when Christian Friedrich Schönbein observed the rapid formation of blue color in the lurid bolete (*Suillellus luridus*) fruiting bodies upon wounding [9]. Four decades later, Émile Bourquelot and Gabriel Bertrand described a color change in the basidiocarp of the blackening brittlegill (*Russula nigricans*) from the initial white over red to black on exposure to air, which they attributed to an oxidase [10]. Tyrosinase got its name from the French biochemist Bertrand in 1896,^1^ who identified it in fungal and plant extracts [11] and could also establish the enzyme’s activity as different from the already known laccase [12]: TYR accepted l-tyrosine as a substrate while laccase did not.

TYRs catalyze the *ortho*-hydroxylation and subsequent two-electron oxidation of monophenols to the corresponding *o*-quinones (tyrosinase activity, EC 1.14.18.1), each coupled to the reduction of molecular oxygen to water (Scheme 1A) [13]. As shown by ^18^O-labelling experiments, the oxygen added to the phenol is derived from molecular oxygen [14]. Catechols (commonly referred to as “*o*-diphenols” in the specialized literature) are usually better TYR substrates than the corresponding monophenols as for these substrates only one of the two enzymatic steps is necessary. Enzymes for which only activity on catechols is reported are generally referred to as catechol oxidases (COs, EC 1.10.3.1). TYRs and COs constitute the enzyme family of polyphenol oxidases (PPOs), a term which is particularly common for plant enzymes, while insect PPOs are commonly called phenol oxidases (POs), and both bacterial and fungal PPOs are mainly referred to as tyrosinases.

A number of PPOs have received special names derived from their respective natural substrate. Those are Larreatricin hydroxylase from *Larrea tridentate* [22], Aureusidin synthase (EC 1.21.3.6) from *Antirrhinum majus* [23], Aurone synthase from *Coreopsis grandiflora* [24], and Pyoverdine maturation tyrosinase from *Pseudomonas aeruginosa* (*Pa*PvdP) [25]. While Aureusidin synthase has even received its own EC number, from a biochemical point of view these four enzymes are PPOs.

**Scheme 4:**
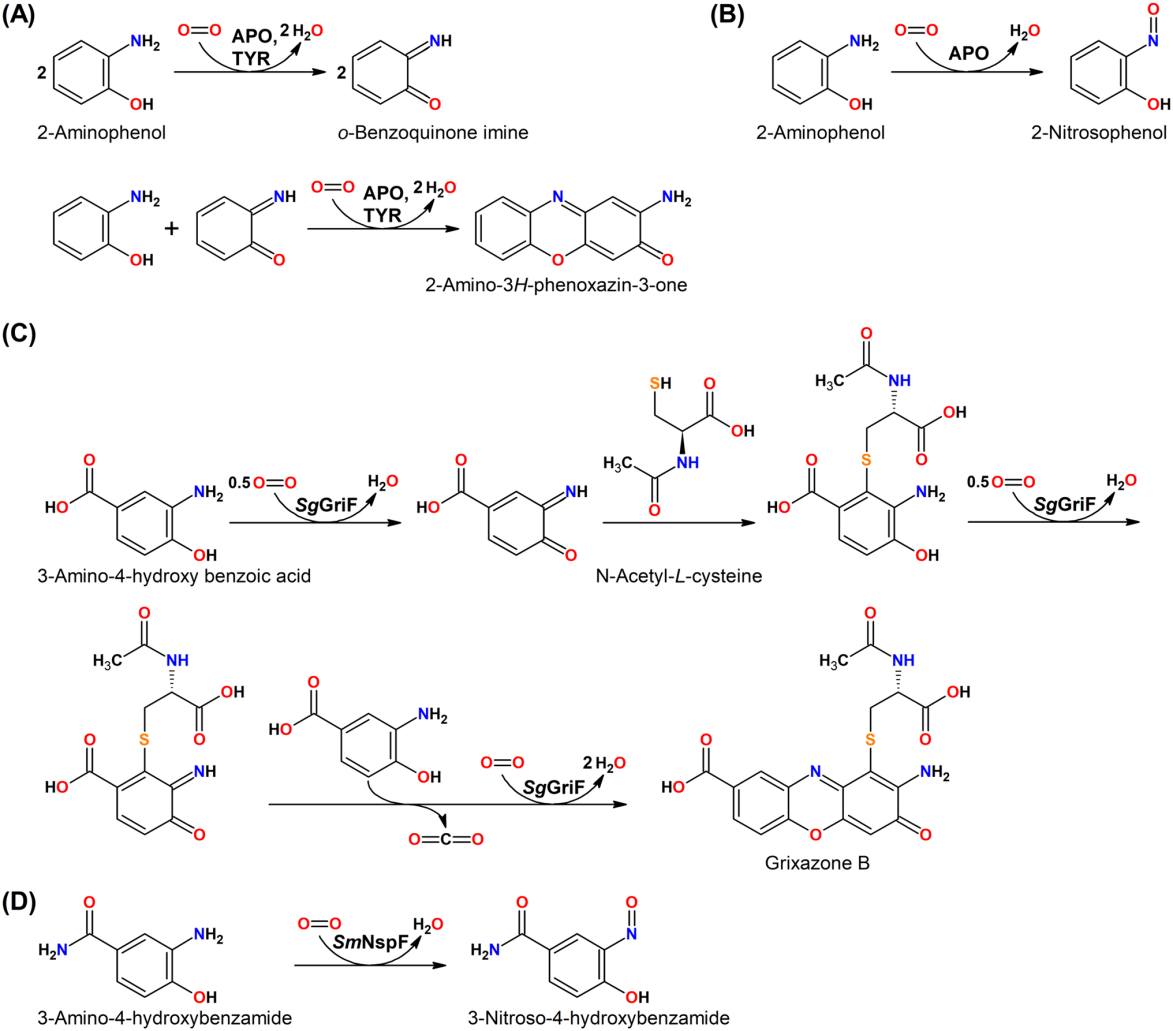
Reactions catalyzed by *Sg*GriF (EC 1.10.3.15, EC 1.10.3.4) and* Sm*NspF (EC 1.10.3.4). **A** Catalytic oxidation of *o*-aminophenols by APOs and formation of the colored phenoxazinone product via 1,4-Michael addition of the *o*-aminophenol and the enzymatic oxidation product followed by two more rounds of enzymatic oxidation leading to ring closure and a tautomeric shift to the final phenoxazinone. **B** Oxidation with APOs leads to the (co-)formation of the nitroso product while non-APO PPOs are only capable of the reactions shown in panel **A**. **C** Proposed biosynthesis pathway of the yellow pigment grixazone B in *Streptomyces griseus* [26]. **D** Proposed biosynthesis of 3-nitroso-4-hydroxybenzamide in *Streptomyces murayamaensis* [27]

A special class within PPOs is formed by enzymes that possess the typical primary sequence and spectroscopic features of tyrosinases but preferentially oxidize *o*-aminophenols (*o*-aminophenol oxidase, APO, EC 1.10.3.4). To date, only two bacterial representatives have been studied: *Sg*GriF from the grixazone biosynthesis gene cluster of *Streptomyces griseus* [26] and *Sm*NspF which is responsible for the final step of 4-hydroxy-3-nitrosobenzamide biosynthesis in *Streptomyces murayamaensis* [27] (Scheme 4).

Enzymatic oxidation of *o*-aminophenols has also been described for the bacterial multi-copper enzyme *Sa*PhsA from *Streptomyces antibioticus* [28]. While this enzyme catalyzes the reaction corresponding to EC 1.10.3.4, structurally it is a laccase that does contain an additional type II copper center that is formed only after the hexamerization of the enzyme [28]. Three eukaryotic enzymes are annotated as *o*-aminophenol oxidases and all three studied enzymes have been designated “isophenoxazine synthases”: These are the plant enzymes from *Tecoma stans* (asterids) [29] and from *Bauhenia monandra* of the pea family [30] as well as a fungal enzyme from the saprotrophic agaricomycete *Pycnoporus coccincus* [31]. While these enzymes do catalyze the oxidation of *o*-aminophenols they are not copper enzymes but are reported to require Mn^2+^ and either flavin adenine dinucleotide (enzyme from *T. stans*) [29] or riboflavin-5′-phosphate (enzyme from *P. coccincus*) [31] as a redox cofactor or even no cofactor at all in the case of the enzyme from *B. monandra* [30]. These cofactor requirements are best considered preliminary as the need for Mn^2+^ was deduced only from an increased reaction rate in the presence of external Mn^2+^. The same methodology was used for determining the need for redox cofactors while the sustained reaction of the *B. monandra* enzyme in the presence of metal chelating agents was construed as evidence against the involvement of metal ions in the catalysis.

Extension of the reactivity of PPOs beyond phenolic compounds has been shown for two fungal enzymes: the tyrosinases from *Neurospora crassa* [16] and *Agaricus bisporus* [32] have been shown to effect the oxidation of aromatic amines, although with rates approximately 1000-fold slower than on the corresponding phenol [16]. The reaction seems to proceed analogous to the standard tyrosinase reaction on phenols, and as for phenolic substrates the incorporation of one atom of oxygen into the oxidized substrate has been demonstrated for anilines using ^18^O_2_ and mass spectrometry of the isolated reaction product [16] (Scheme 1B).

### Copper

The transition metal copper is present at an average of approximately 55 ppm in the earth’s crust [33], making it the 23rd most abundant element available via mining. Its elemental symbol Cu comes from its Latin name *Cuprum* which is derived by contraction of the Latin *Cyprium aes* (ore from Cyprus) and Greek Κύπρος *Kyprios* (Cyprus, “land of cypresses”) as this island used to be (and still is) an important source of copper ore [34]. Copper exists as two stable isotopes: 69% $_{29}^{63}{\text{Cu}}$, 31% $_{29}^{65}{\text{Cu}}$ [35]; in its metallic state it has the electron configuration 1s^2^2s^2^2p^6^3s^2^3p^6^3d^10^4s^1^. Copper exhibits the oxidation states 0, +1, +2, and +3, where 0 is the metallic state. The +3 state is present in a number of copper fluorides and oxides [36] and has also been postulated in a newly proposed reaction mechanism for tyrosinase [37]. Cuprates, a class of ceramic high-temperature superconductors, also contain Cu(III). The first superconducting material with a critical temperature above the boiling point of liquid nitrogen (77 K) to be discovered was YBa_2_Cu_3_O_7_ (critical temperature 93 K) [38], which contains two Cu(II) ions and one Cu(III) ion in its unit cell [39]. The current record for highest critical temperature of a cuprate superconductor is held by the Tl-substituted Hg-Ba-Ca-cuprate Hg_0.8_Tl_0.2_Ba_2_Ca_2_Cu_3_O_8+δ_ (δ represents a small increase in oxygen content induced by annealing of the ceramic in an oxygen atmosphere), which becomes superconductive below 138 K [40].

Standard redox potentials are Cu^+^/Cu = 0.518 V, Cu^2+^/Cu = 0.339 V (two transferred electrons), Cu^2+^/Cu^+^ = 0.161 V, and Cu^3+^/Cu^2+^  = 2.4 V [41]. The high redox potential difference between Cu(III) and Cu(II) indicates a significant energy barrier for attaining the 3d^8^ state of Cu(III) and in accordance with that notion, the metal–ligand bonds of Cu(III) complexes are usually highly covalent, even in the case of strongly electronegative ligands [42]. Suitable ligands can decrease the Cu^3+^/Cu^2+^ redox potential dramatically: in the presence of potassium periodate the redox potential of Cu^3+^/Cu^2+^ at pH 12.2 was 0.617 V [43].

**Fig. 1:**
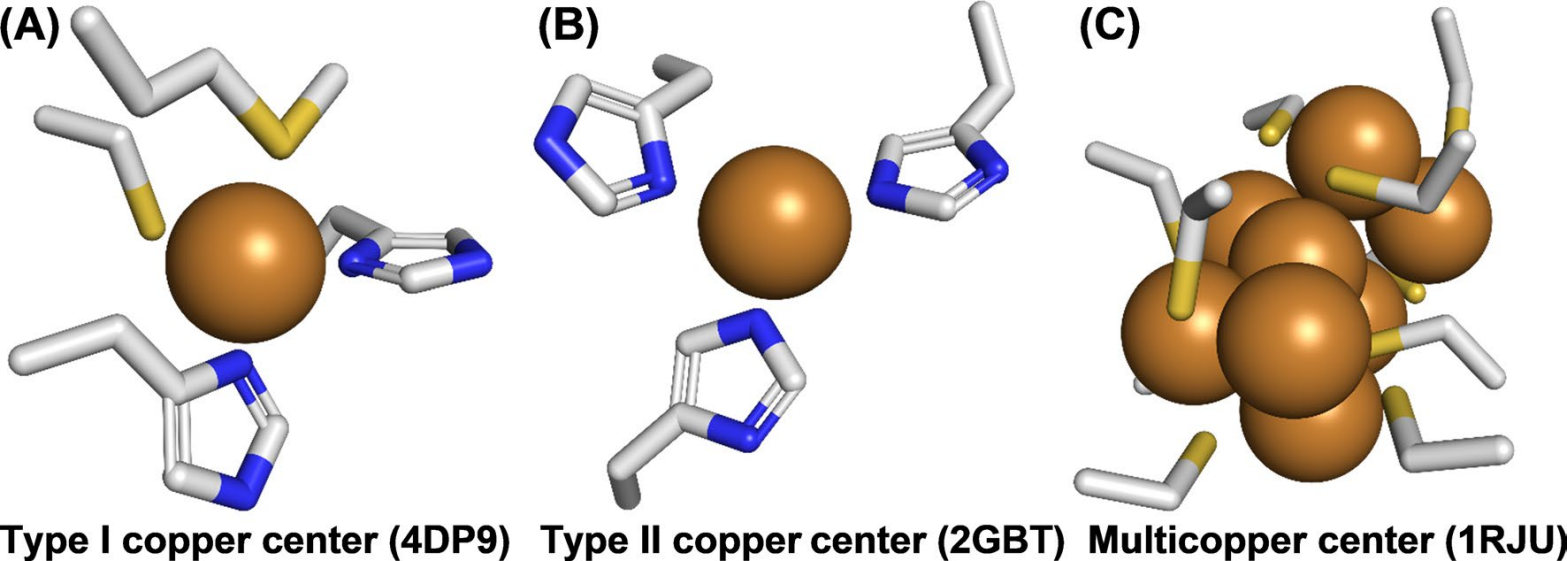
Type I, II, and multi-copper centers. Representative examples for the main types of copper sites are given with **A** poplar plastocyanin (PDB 4DP9) [46] showing the typical coordination sphere of a type I copper center made up of two histidines and one cysteine in a trigonal geometry and a sulfur from a methionine in the axial position, **B** human CuZn superoxide dismutase (PDB 2GBT) [49] with its type II copper center made up of three histidines, and **C** the copper thionein from baker’s yeast (PDB 1RJU) [55] as an example of a multi-copper center. This multi-copper protein contains eight copper ions that are kept in place by ten cysteines. The copper ions are depicted as brown spheres, and the coordinating amino acid side chains are drawn as sticks colored according to the type of atom they contain: carbon (gray), nitrogen (blue), and sulfur (yellow)

The +1 (*cuprous*, electron configuration [Ar]3d^10^) and +2 (*cupric*, [Ar]3d^9^) states are most abundant in copper species and it is also those states that are responsible for virtually all of the biochemistry of copper [44]. Formation of the +2 state removes an electron from the 3d shell and copper is therefore a transition metal. Cu^+^ is a soft metal ion and consequently it prefers soft ligands like sulfur and iodine. Cu^2+^ on the other hand is a borderline/hard metal ion that is more likely to be complexed with hard ligands like nitrogen, chloride, or oxygen. In proteins the available ligands are mostly restricted to amino acid side chains which translates to a preference for Cu(I) coordination by cysteine or methionine (via their thiol or thioether group, respectively; Fig. 1A) and Cu(II) coordination by histidine (via an imidazole nitrogen; Fig. 1B). The different occupation of the five d orbitals (Cu^+^, d^10^; Cu^2+^, d^9^) dictates the different coordination geometries: Cu^+^ prefers tetrahedral coordination while Cu^2+^ is more likely to be present in a octahedral geometry that is distorted by the Jahn–Teller effect due to a twofold degenerate orbital ground state (nine electrons in an octahedral ligand field) [44, 45]. In metalloenzymes copper is often present in a slightly distorted tetrahedral geometry that is maintained during the redox cycling between Cu^+^ and Cu^2+^. For Cu^2+^ this is a high energy geometry that is likely to enhance the reactivity of the metal by increasing its redox potential [45].

### Copper active sites

The classical division of copper-containing proteins is based on their spectroscopic properties, specifically the UV–Vis and electron paramagnetic resonance (EPR) spectra. Type I (mononuclear, Fig. 1A) or “blue copper proteins” exhibit a strong absorption of electromagnetic waves of wavelengths around 600 nm, hence their intense blue color, and are most commonly involved in electron transfer processes, e.g., in photosynthesis and the respiratory chain. Examples of metalloproteins with type I copper centers include the electron transporters plastocyanin (photosynthesis) [46], azurin (bacterial cytochrome chain) [47], and the multi-copper enzyme l-ascorbate oxidase (EC 1.10.3.3) [48].

Type II (mononuclear, Fig. 1B) or “non blue copper proteins” possess a typical Cu^2+^ EPR signal, while no such signal is present for type III copper centers, presumably as a result of strong spin polarization leading to antiferromagnetic coupling of the spin states on both copper ions [44]. Type II copper centers exist in oxygen-activating enzymes, where they form the active centers of, e.g., superoxide dismutase (EC 1.15.1.1) [49], galactose oxidase (EC 1.1.3.9) [50], lysyl oxidase (EC 1.4.3.13) [51], and copper amine oxidase (EC 1.4.3.21) [52].

**Fig. 2:**
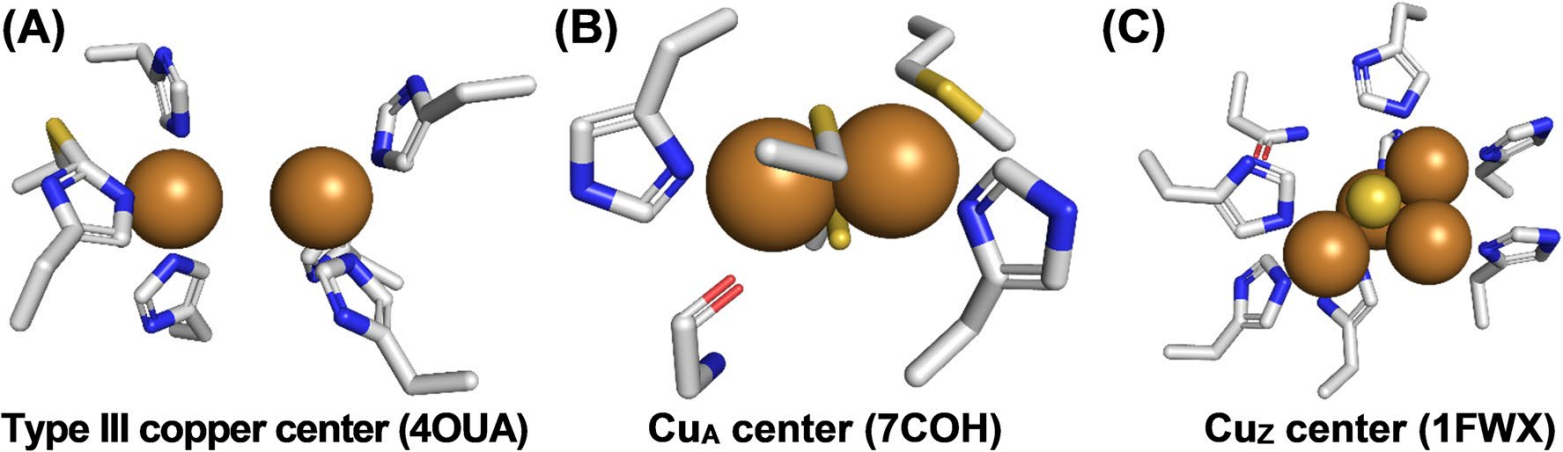
Type III copper, Cu_A_ and Cu_Z_ active sites. **A** Shows the type III copper center of the fungal TYR *Ab*PPO4 from *Agaricus bisporus* (PDB 4OUA) [62]. It contains two copper ions that are coordinated by the nitrogens of three histidines each. One of the histidines displays a modification typical for tyrosinases of eukaryotic origin: a thioether bond between the γ-S of a neighboring cysteine and C-2 in the imidazole ring [63]. **B** The Cu_A_ center of the bovine cytochrome* c* oxidase (PDB 7COH) [64] contains two copper ions that are coordinated by two histidines, two cysteines, one methionine, and one main-chain oxygen (from C=O of a peptide bond). **C** Depicts the Cu_Z_ center of the nitrous oxide reductase from the bacterium *Paracoccus denitrificans* (PDB 1FWX) [65]. Cu_z_ contains four copper ions that are coordinated by seven histidines and one asparagine. Notably, an inorganic sulfur is present between the four copper ions as a sulfide ion. The elements present in the copper-coordinating amino acids (drawn as sticks) are color-coded using gray for carbon, blue for nitrogen, red for oxygen, and yellow for sulfur. The copper ions themselves are depicted as brown spheres and the inorganic sulfide (S^2−^) coordinated at the Cu_Z_ center is shown as a small yellow sphere

Type III copper centers (Fig. 2A) form the active sites of PPOs, bind oxygen in the hemocyanin oxygen transport proteins, and are part of the trinuclear copper cluster that reduces oxygen to water in laccases. Later additions to these three classical types of copper centers are the dinuclear Cu_A_ center (strong absorption around 500 nm and a 7-line EPR signal) present in cytochrome *c* oxidase [53] (Fig. 2B) and the tetranuclear Cu_Z_ center (strong absorption at ≈640 nm and a 2 × 4 line EPR spectrum) in nitrous oxide reductase [54] (Fig. 2C).

#### Type III copper centers

The active site of PPOs is formed by a type III copper center whose two copper ions are coordinated by three histidyl residues each, namely via their Nε-nitrogens (i.e., the ones farther away from the respective peptide backbone atoms) [56] (Fig. 2A). Type III copper centers bind molecular oxygen as peroxide in a characteristic “side-on” (µ-η^2^: η^2^-O_2_) binding mode [57] yielding the oxy-form of the protein. Besides PPOs, type III copper centers are present in hemocyanins, the oxygen-transporting proteins present in the hemolymph of arthropods and mollusks [58], and they also form part of the trinuclear copper cluster of laccases [59].

While the peroxide-binding ability is a general feature of type III copper proteins it is not sufficient for PPO activity: hemocyanins are oxygen transporters, not enzymes (but TYR activity can be induced by a detergent treatment [60]) and laccases, in which the peroxide form is only a short-lived intermediate, oxidize their substrates via a radical mechanism and keep them well away from the site of oxygen reduction [61].

### Structural features of PPOs

**Fig. 3:**
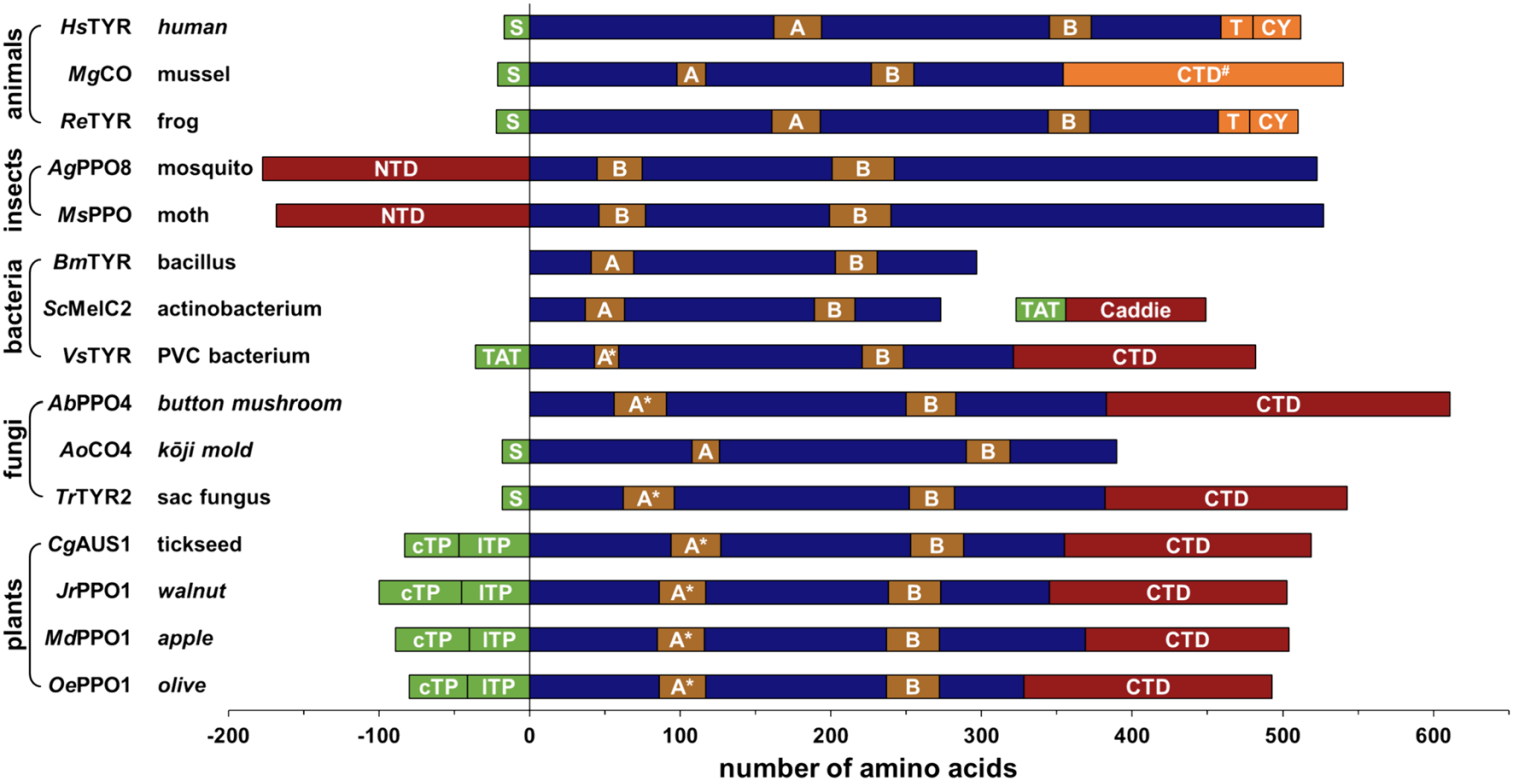
Architecture of PPOs. The central TYR domain (Pfam [73] entry PF00264) is drawn in blue, amino acid numbering starts at its beginning. N-terminal signal peptides (*S* secretory signal peptide recognized by the Sec translocon and signal peptidase I, *TAT* twin arginine signal peptide recognized by the Tat translocon and signal peptidase I, *cTP* chloroplast transit peptide, *lTP* TAT-transfer peptide targeting the protein to the thylakoid lumen) are shown in green and domains covering the active site are depicted in red (*NTD* N-terminal active-site shielding domain of insect PPOs, *Caddie* MelC1 caddie protein of *Streptomyces* tyrosinases—Pfam entry PF06236, *CTD* C-terminal lid-like domain). The caddie (MelC1) protein of *Streptomyces* tyrosinases is part of the *mel*-operon and is thereby transcriptionally linked to the tyrosine MelC2 (i.e., both caddie and TYR are encoded by the same mRNA molecule) [74]. The regions of the central domains containing the copper-coordinating histidines are depicted in brown (A: CuA-motif, B: CuB-motif, A*: CuA-motif of a PPO with a thioether bridge at HisA_2_). In insect PPOs both copper sites are of the CuB type [75], i.e., all six copper-coordinating histidines of these PPOs are located on α-helices. The C-terminal region of animal PPOs does not cover the active site and is drawn orange (*CTD*^*#*^ C-terminal domain of a PPO that does not cover the active site), in human TYR it is responsible for membrane integration and consists of a transmembrane helix (T) and a small tail that protrudes into the cytosolic space (CY) of the melanosome [76]. The *common name of the organism* (in italics) or the group it belongs to (in plain text) is provided next to the name of the enzyme. Presented are the sequences of *Hs*TYR from *Homo sapiens* (UniProt-ID P14679), *Mg*CO from *Mytilus galloprovincialis* (A0A6B7E5P4), *Re*TYR from *Rana esculenta* (Q0MVP0), *Ag*PPO8 from *Anopheles gambiae* (Q8MZM3), *Ms*PPO from *Manduca sexta* (Q25519), *Bm*TYR from *Bacillus megaterium* (B2ZB02), *Sc*MelC2 from *Streptomyces castaneoglobisporus* (Q83WS2, *Sc*MelC1: Q83WS1), *Vs*TYR from *Verrucomicrobium spinosum* (NCBI-ID WP_009958178), *Ab*PPO4 from *Agaricus bisporus* (UniProt-ID C7FF05), *Ao*CO4 from *Aspergillus oryzae* (Q2UNF9), *Tr*TYR2 from *Trichoderma reesei* (A0ZXZ4), *Cg*AUS1 from *Coreopsis grandiflora* (A0A075DN54), *Jr*PPO1 from *Juglans regia* (C0LU17), *Md*PPO1 from *Malus domestica* (P43309), and *Oe*PPO1 from *Olea europaea* (NCBI-ID QXT58294)

In general, PPOs may contain an N-terminal signal peptide, a central domain which comprises the type III copper center responsible for the enzymatic activity and a C-terminal domain that covers the active site [66]. Of these features, the central domain is present in all PPOs while the other two elements are optional (for examples, see Fig. 3 and Table 2).

**Fig. 4:**
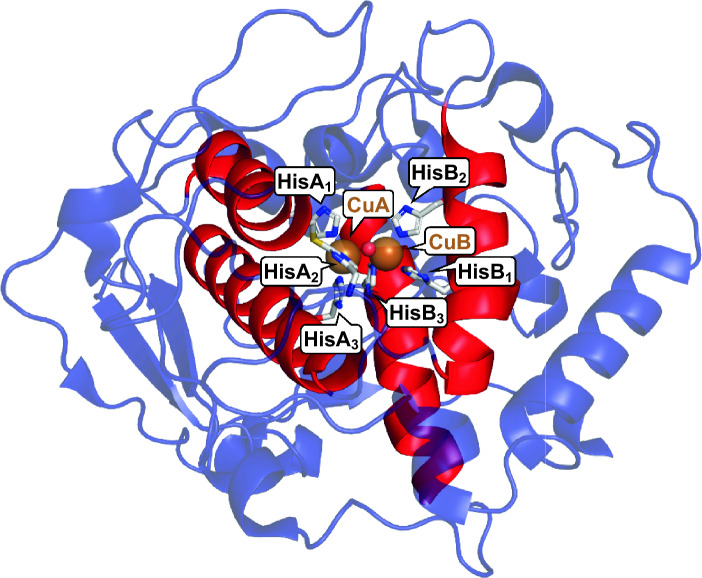
Central domain of tyrosinases: TYR from* Juglans regia* (*Jr*PPO1, PDB 5CE9 [68]). The protein backbone is shown in blue, red highlights the four α-helices carrying five of the six Cu-coordinating histidines (HisA_2_ is located on a loop.) that are found in all type III copper proteins. Coordinated copper ions are depicted as brown spheres, while the small red sphere in between the copper ions indicates a coordinated water or hydroxide ion. The amino acid side chains forming the active center (CuA: HisA_1–3_, the thioether bridge formed between HisA_2_ and an adjacent cysteine, CuB: HisB_1–3_) are depicted as sticks colored grey for carbon, blue for nitrogen and yellow for sulfur

Within the central domain a four α-helix bundle common to all type III copper proteins is found that typically provides five of the six copper-coordinating histidines (Fig. 4, α-helix bundle drawn in red). These histidines are addressed according to the copper ion they coordinate (CuA or CuB) and their relative position in the protein’s primary sequence: HisA_[1–3]_ are closer to the protein’s N-terminus and coordinate CuA while HisB_[1–3]_ have higher sequence numbers and keep CuB in place. H–X_*n*_–H–X_8_–H forms the CuA-motif, while the CuB-binding region is characterized by the motif H–X_3_–H–X_*n*_–H [67]. In these motifs H stands for a Cu-coordinating histidine, X denominates any amino acid, and* n* is an integer usually around or greater than 20 but may also be smaller than 10 in the CuA motif of some bacterial tyrosinases. Note that all insect PPOs characterized so far feature two CuB sites instead of the standard type III copper protein configuration of a CuA and a CuB site [66]. In most plant and fungal enzymes HisA_2_ forms a thioether bridge with a spatially close cysteine [63] (Fig. 2A). This histidine is the one among the six ligands forming the type III copper center that is located on a (more flexible) loop while the other five are sustained by two of the conserved (quite rigid) α-helices (Fig. 4). The thioether bridge rigidifies the position of HisA_2_ and in so doing may help fine-tune the electronic structure of the type III copper center [56].

Non-insect animal PPOs usually have a signal sequence targeting the enzyme to a pigment-forming organelle like the melanosome [69] but the presence of a C-terminal domain is rare among these enzymes (Fig. 3). Insect PPOs have a different architecture with an N-terminal domain that takes over the role of covering the active site [70] and also two CuB sites [66] instead of the usual sequence of CuA and CuB. Bacteria either produce PPOs that consist only of the central domain (e.g., PPOs from *Bacillus* or *Streptomyces* species) or PPOs that also contain a signal sequence and a C-terminal domain (e.g., PPOs from *Pseudomonas* or *Verrucomicrobium*). In fungal PPOs the presence of a signal sequence is rare, leading to a cytosolic location of most of these enzymes, and the vast majority of fungal PPOs possess a C-terminal domain. The situation is reversed in the class of short fungal PPOs that do have a signal sequence targeting them out of the cell but do not contain a C-terminal domain [71]. The majority of plant PPOs are translated with two signal peptides in series that target the freshly synthesized enzyme first to the chloroplast and then further into the thylakoid lumen of this subcellular organelle [72]. All plant PPOs biochemically characterized so far possess both a signal sequence and a C-terminal domain shielding the active site.

#### Role of the C-terminal domain of PPOs

**Fig. 5:**
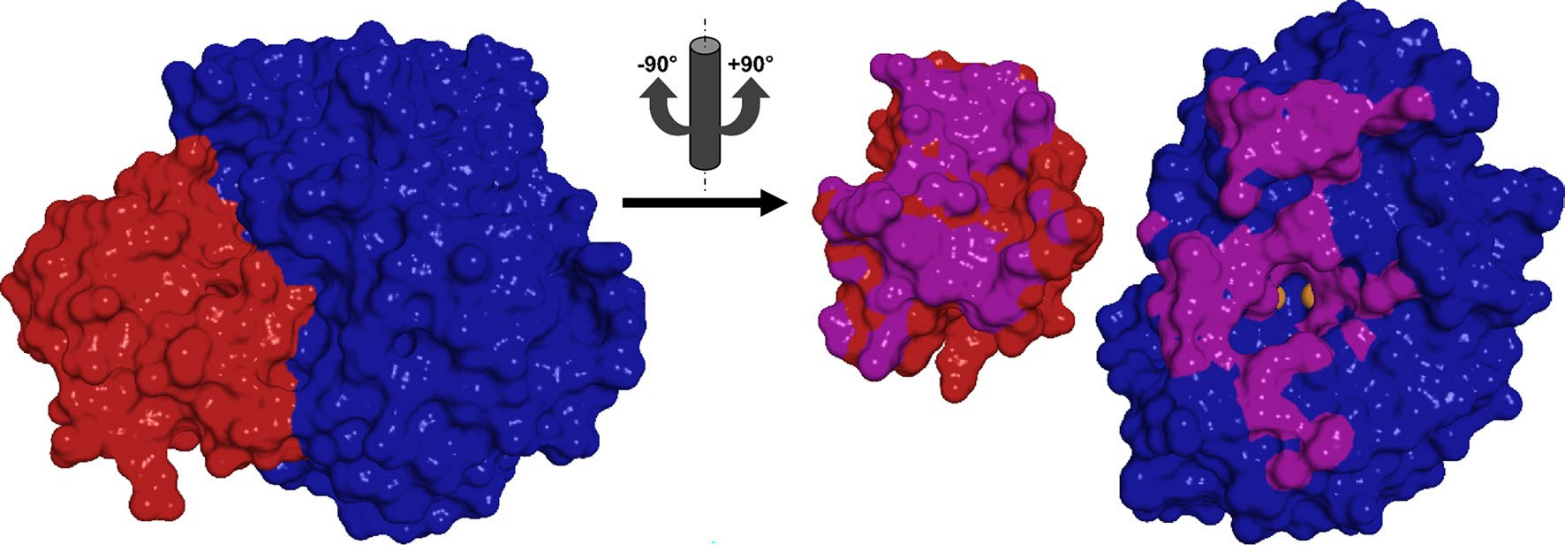
Bacterial tyrosinase from* Streptomyces castaneoglobisporus* (*Sc*MelC1 + 2, PDB entry 5Z0D [80]). Red indicates amino acids of the caddie protein (*Sc*MelC1, 13 kDa), the bacterial tyrosinase* Sc*MelC2 (31 kDa) is shown in blue. On the left the complex of both proteins prior to activation of the TYR is depicted. On the right the two proteins are rotated by ± 90° relative to the left picture and the protein interface (all contacts closer than 4 Å) is highlighted in violet. The brown spheres visible in the right picture indicate the two copper ions of the TYR’s active site

In prokaryotic PPOs from *Streptomyces* species the C-terminal domain is expressed as a separate protein, the so-called caddie protein [77] (Fig. 5) that is also responsible for introduction of copper to the active site [78] and secretion of the PPO from the cell [79]. Following export from the cell and loading of the bacterial TYR with copper the tyrosyl amino acid side chain of the caddie protein that extends into the active center of the TYR gets oxidized, which putatively causes the caddie to dissociate from the TYR and then to precipitate [80].

**Fig. 6:**
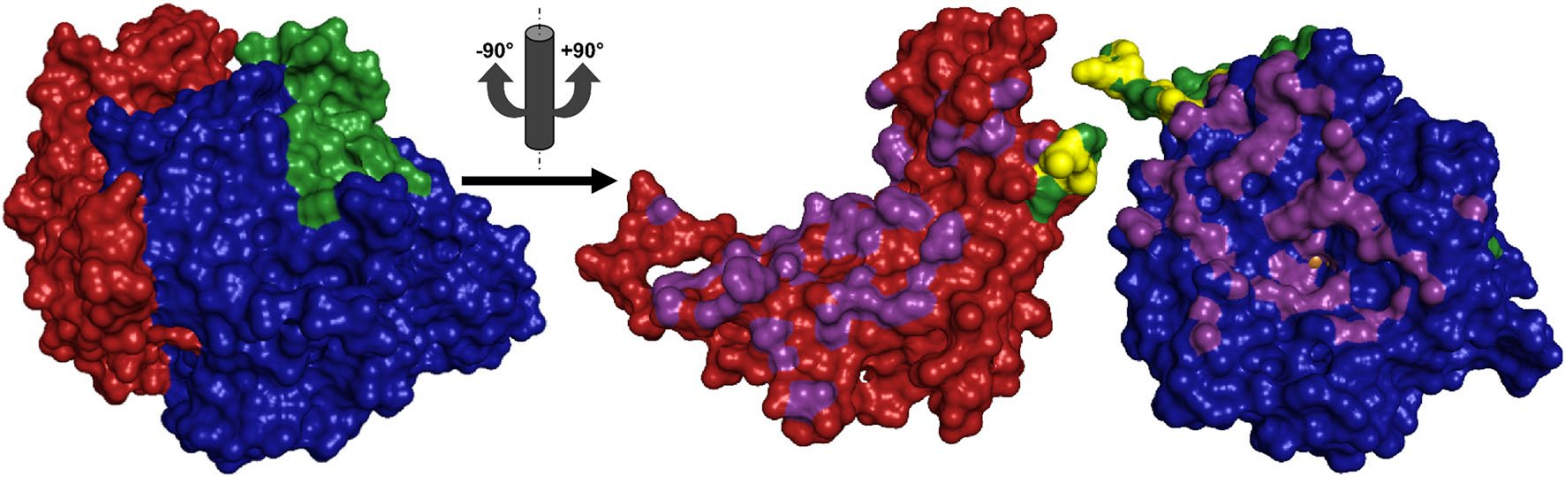
Fungal tyrosinase from *Agaricus bisporus* (*Ab*PPO4, PDB entry 5M6B [82]). Green indicates the linker region (53 amino acids) between the catalytically active domain (blue) and the lid-like, shielding C-terminal domain (red). On the right the situation after proteolytic cleavage is shown with the resulting N- and C-terminal fragments rotated by ± 90° and the interdomain interface (contacts up to 4 Å) highlighted in violet and yellow. Activation yields a 44 kDa N-terminal fragment, the active form of *Ab*PPO4. The two brown spheres in the right picture represent the copper ions of the active site (with the second copper ion barely visible to the right of the first) and the C-terminal domain was shifted downwards in order to allow for a better use of space

The domain nature of the C-terminal extension of PPOs has been experimentally verified, as independent folding of a eukaryotic C-terminal domain has recently been demonstrated for a TYR from apple [81]. PPOs with their C-terminal domain in place (Fig. 6) usually display no or only very limited enzymatic activity and are therefore referred to as latent PPOs. Removal of the C-terminal domain exposes the type III copper center to potential substrates, yielding the enzymatically active form of the PPO [62]. Especially in the context of plant and insect PPOs the active form is often designated by the term “mature” PPO and consequently “maturation” is a commonly encountered term used to refer to the activation process (i.e., removal of the lid-like domain).

#### Autoactivation of PPOs

PPO activation may be effected by external proteases, as has been shown for *Manduca sexta* PPO [83] but for non-insect PPOs the physiological activation mechanism remains elusive. Autoactivation independent of external factors has been reported for two plant PPOs, *Pa*PPO from *Prunus armeniaca* [84] and *Md*PPO1 from *Malus domestica* [81], as well as for the fungal enzyme *Ab*PPO2 from *Agaricus bisporus* [85].

**Fig. 7:**
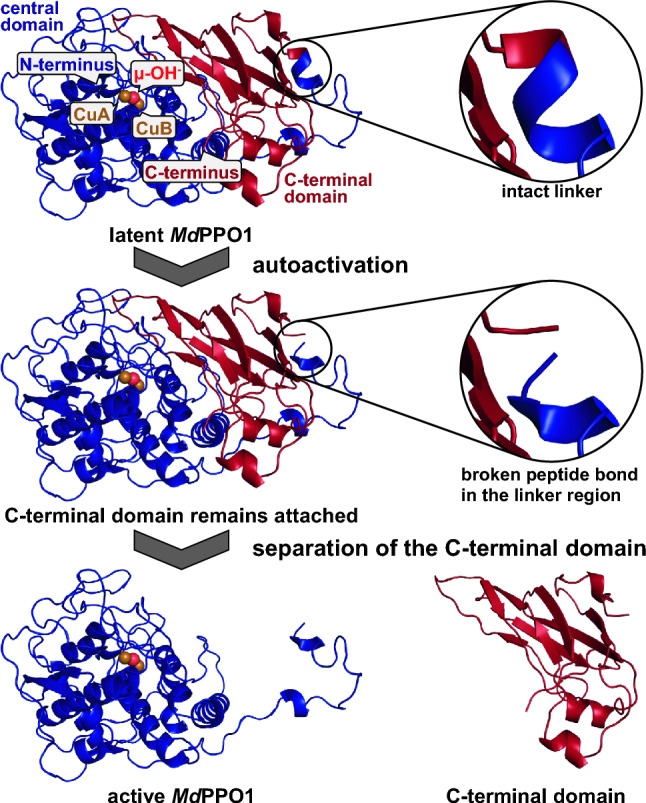
Autoactivation of the plant tyrosinase *Md*PPO1 from *Malus domestica* (PDB entry 6ELS [81]). *Md*PPO1 activates itself via the cleavage of a peptide bond in the linker region between the central domain that carries the type III copper center and the lid-like C-terminal domain that covers the active site of the enzyme. Even after the cleavage of the protein backbone the C-terminal domain remains attached to the central domain of *Md*PPO1 [81], leaving the enzyme in the latent state. PISA analysis [86] of the protein interface detects 25 hydrogen bonds and 7 salt bridges that stabilize the complex of the central and C-terminal domains of *Md*PPO1. Separation of the C-terminal domain from the central tyrosinase domain can be achieved by applying high concentrations of salt (e.g., 1 M NaCl or 0.15 M MgCl_2_) to the autoactivated *Md*PPO1 [81]. The central domain of *Md*PPO1 is drawn as blue cartoon while the C-terminal domain is shown in red. The two copper ions coordinated at the type III copper site of the central domain are drawn as brown spheres, the red sphere between them indicates a bridging hydroxide ion. This figure was prepared using PyMOL 2.6 (Schrödinger LLC)

**Fig. 8:**
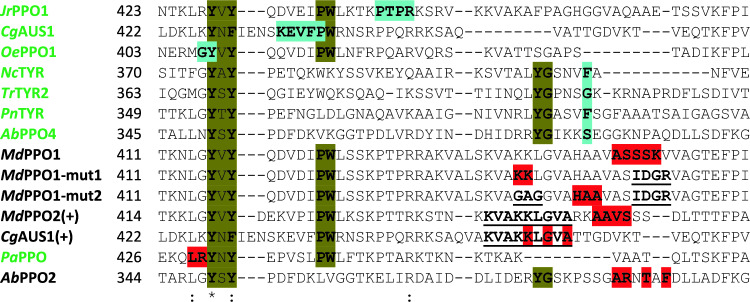
Cleavage positions of PPOs matured in vivo and sites of autoactivation in vitro*.* Enzymes that were isolated from the respective natural source are written in green while PPOs produced heterologously in *Escherichia coli* are shown with their name in black. The tyrosine motif (Y/F–X–Y or Y–X–Y/F) present near the end of the active domain in all PPOs [91] and the PW motif indicating the end of the active domain of plant PPOs as well as the YG motif found at the end of the active domain of fungal PPOs [87] are marked in light olive. The C-termini of PPOs isolated as mature enzymes from their respective host organism are highlighted using light blue while the experimentally determined C-terminal amino acids of in vitro autoactivated PPOs are highlighted in red. Amino acid exchanges that resulted in altered autoactivation behavior [81] are underlined. PPOs isolated from plants for which the exact location of their C-termini were determined are *Jr*PPO1 from *Juglans regia* (UniProt-ID C0LU17) [88], *Cg*AUS1 from *Coreopsis grandiflora* (A0A075DN54) [24], and *Oe*PPO1 from *Olea europaea* (NCBI-ID QXT58294) [89]. The location of the C-terminus was also determined for the fungal TYRs isolated from the respective fungi: *Nc*TYR from *Neurospora crassa* (P00440) [92], *Tr*TYR2 from *Trichoderma reesei* (A0ZXZ4) [93], *Pn*TYR from *Pholiota nameko* (A7BHQ9) [94], and *Ab*PPO4 from *Agaricus bisporus* (UPI00029F7750) [95]. Autoactivation in vitro was observed for the two plant TYRs *Pa*PPO from *Prunus armeniaca* (O81103) [84] and *MdPPO1* from *Malus domestica* (A0A238GSN3) [81] as well as for the fungal TYR *Ab*PPO2 from *A. bisporus* (CAG9553204) [85]. Insertion of the nonapeptide KVAKKLGVA from *Md*PPO1 into the sequences of the originally stable enzymes *Md*PPO2 from *M. domestica* (A0A238GSS3) and *Cg*AUS1 produced the autoactivating mutants *Md*PPO2(+) and *Cg*AUS1(+) while modification of the amino acids cleaved during autoactivation of *Md*PPO1 (*Md*PPO1-mut1 and *Md*PPO1-mut2) did not prevent autoactivation but merely caused the site of autoactivation to move [81]. Amino acid positions are counted from the initial methionine (M1) encoded in the gene of each protein and take the signal sequences of the plant PPOs (*Jr*PPO1, M1–A100; *Cg*AUS1, M1–A85; *Oe*PPO1, M1–A80; *Md*PPO1, M1–A89; *Md*PPO2, M1–A92; and *Pa*PPO, M1–A101) into account. Alignment of the protein sequences was performed with MAFFT (v7.525) [96]

These spontaneous activation reactions proceed via hydrolysis of a peptide bond in the loop linking the central and the C-terminal domain of these PPOs (Fig. 7) [81]. The location of this cleavage coincides with the region of the experimentally determined C-termini of four fungal tyrosinases [87] as well as with those of the three plant enzymes *Jr*PPO1 from *Juglans regia* (walnut) [88], *Cg*AUS1 from *Coreopsis grandiflora* (large-flowered tickseed) [24], and *Oe*PPO1 from *Olea europaea* (olive) [89] that were all isolated from their respective natural sources (Fig. 8).

Autoactivation of the plant enzymes *Md*PPO1 [81] and *Pa*PPO [84] results in the release of the intact C-terminal domain while the lid-like domain of the fungal enzyme *Ab*PPO2 [85] becomes disintegrated completely during the autoactivation of the enzyme. The C-terminal domain of *Md*PPO1 crystallized out of a solution containing latent *Md*PPO1, and the corresponding crystal structure (PDB ID 6ELT) [81] shows no modification to any amino acid in the C-terminal domain.

A proteolytic activity with striking similarity to the autoactivation of PPOs has been reported for the fungal TYR *Ab*PPO4 from *Agaricus bisporus*: *Ab*PPO4 was shown to exert proteolytic activity towards a lipase from *Clostridium botulinum* that results in the removal of a 71 amino acid peptide covering the lipase’s active site, which greatly enhances the enzymatic activity of the bacterial lipase [90]. As in PPO autoactivation the cleaved peptide bond is solvent-exposed and located in a protruding part of the enzyme. Furthermore, exchange of the amino acids at the cleavage site did not stop the proteolysis by the PPO [90]. To date, this remains the only report of a PPO showing proteolytic activity towards another protein or peptide.

#### Catalytically important amino acids

**Fig. 9:**
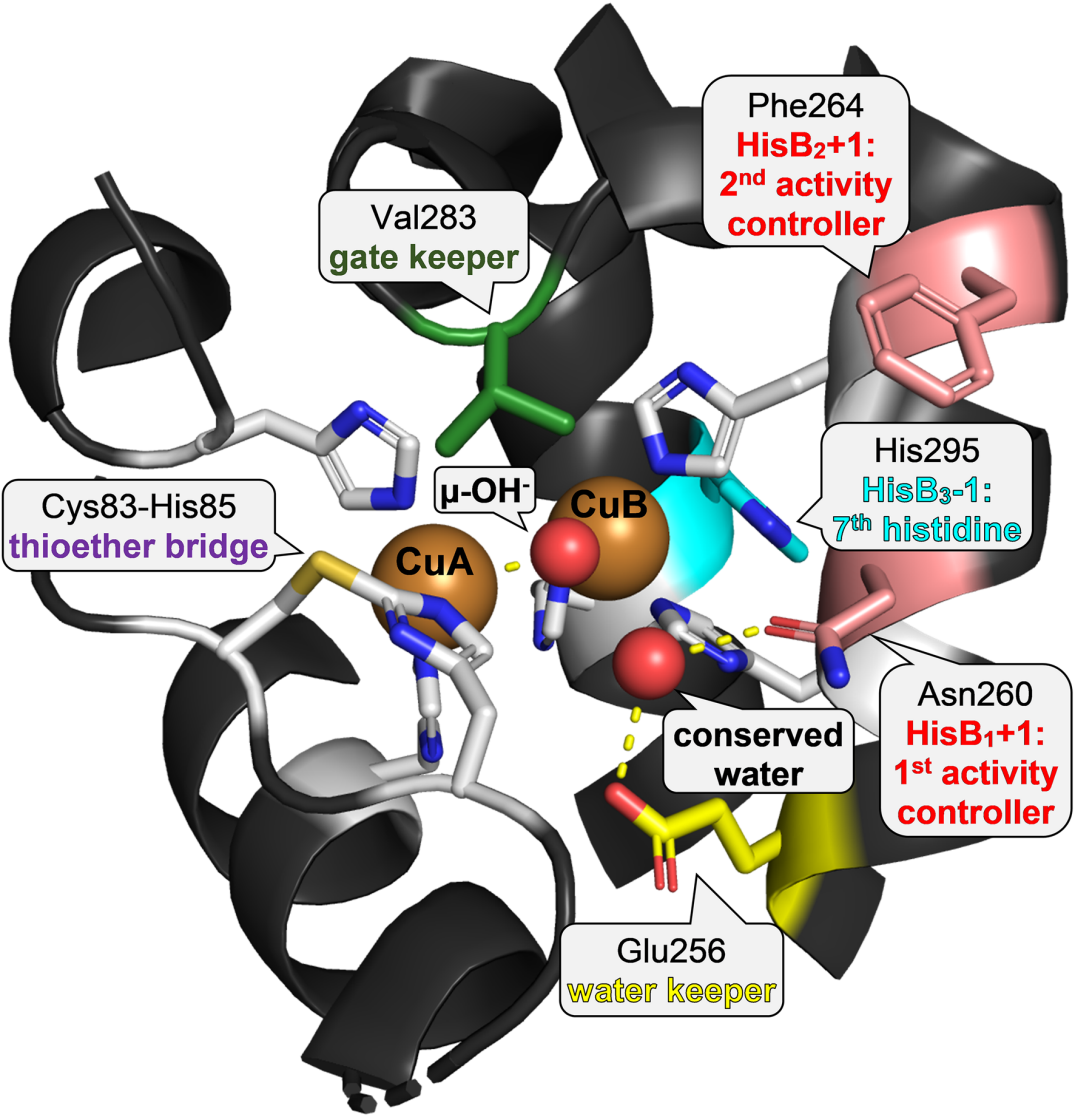
Amino acids that modulate the enzymatic activity of PPOs. Depicted is the active center of *Ab*PPO3 from *Agaricus bisporus* (PDB ID 2Y9W) [99]. Loops and helices around the active site are shown as dark gray cartoons. The side chains of the six copper-coordinating histidines [56], the 7th histidine which has been suggested as an alternative ligand to CuB [62], the two amino acids immediately following the 1st and 2nd histidine of CuB termed “activity controllers” owing to their pronounced influence on the enzymatic activity of PPOs [100], the gate keeper protruding into the active site and modulating the enzyme’s activity [98], and the water keeper residue named for the water molecule it keeps in place close to the copper ions [101] are depicted as sticks with color-coded carbon atoms (copper-coordinating histidines and the cysteine that forms the thioether bridge [63]—white; 7th histidine—light blue; gate keeper—green; activity controllers—salmon; water keeper—yellow), nitrogen atoms in blue, oxygen atoms in red, and sulfur atoms in orange. The two copper ions of the type III copper center are shown as brown spheres, the smaller red spheres depict the oxygen coordinated by both copper ions and the “conserved water” molecule that is kept in place by the water keeper and the asparagine at the 1st activity controller position. The figure was prepared using PyMOL 2.6 (Schrödinger LLC)

**Fig. 10:**
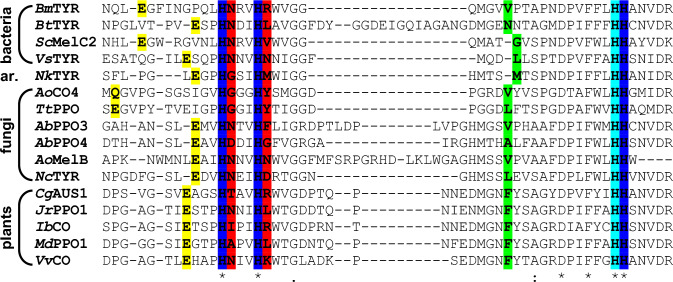
Amino acid sequences of selected PPOs around the CuB site. Amino acids implicated in modulating the enzymatic activity of PPOs are highlighted: the highly conserved glutamic acid (“water keeper”, glutamine in *Ao*CO4) that is thought to stabilize a water molecule [101] in yellow, the three copper-coordinating histidines of CuB [56] in blue, the two amino acids adjacent to the first and second histidine of CuB (HisB_1_ + 1 and HisB_2_ + 1, the two “activity controllers”) [113] in red, the “gate keeper” residue close to the entrance of the active site [98] in green, and the 7th histidine that is highly conserved but does not coordinate a copper ion in light blue. Shown are the sequences of the bacterial enzymes *Bm*TYR (from *Priestia megaterium*, formerly called *Bacillus megaterium*; UniProt-ID B2ZB02), *Bt*TYR (*Burkholderia thailandensis*, Q2T7K1), *Sc*MelC2 (*Streptomyces castaneoglobisporus*, Q83WS2), and *Vs*TYR (*Verrucomicrobium spinosum*, NCBI-ID WP_009958178), the archeal (labeled ar.) enzyme *Nk*TYR (*Candidatus Nitrosopumilus koreensis* AR1, K0B2W2), the two short fungal PPOs [71] *Ao*CO4 (*Aspergillus oryzae*, Q2UNF9) and *Tt*PPO (*Thermothelomyces thermophilus*, G2QLD3), followed by the fungal tyrosinases *Ab*PPO3 (*Agaricus bisporus*, C7FF04), *Ab*PPO4 (*A. bisporus*, C7FF05), *Ao*MelB (*A. oryzae*, Q2UP46), and *Nc*TYR (*Neurospora crassa*, P00440) as well as the PPOs from higher plants *Cg*AUS1 (*Coreopsis grandiflora*, A0A075DN54), *Jr*PPO1 (*Juglans regia*, C0LU17), *Ib*CO (*Ipomoea batatas*, Q9ZP19), *Md*PPO1 (*Malus domestica*, A0A238GSN3), and *Vv*CO (*Vitis vinifera*, P43311). The alignment was prepared with T-Coffee [96]. *A* alanine, *C* cysteine, *D* aspartic acid, *E* glutamic acid, *F* phenylalanine, *G* glycine, *H* histidine, *I* isoleucine, *K* lysine, *L* leucine, *M* methionine, *N* asparagine, *P* proline, *Q* glutamine, *R* arginine, *S* serine, *T* threonine, *V* valine, *W* tryptophan, *Y* tyrosine

The search for the structural factors behind the observed different catalytic activities of type III copper enzymes, primarily the distinction between TYRs and COs (Scheme 1), has been going on for decades. Given that structural data on COs predates equivalent information on TYRs by slightly more than 7 years, the first theories regarding the structural basis of the observed biochemical differences were formulated on the basis of the crystal structure of a single CO, *Ib*CO from sweet potato [56]. With more structural and biochemical data on both TYRs and COs becoming available later on, a number of hypotheses aimed at explaining the observed differences in substrate acceptance between these enzyme families have been formulated [97, 98]. The locations of the amino acids implicated as relevant to the enzymatic activity of PPOs are depicted in Fig. 9 and the actual amino acids at these positions in representative PPOs are shown in Fig. 10.

**Fig. 11:**
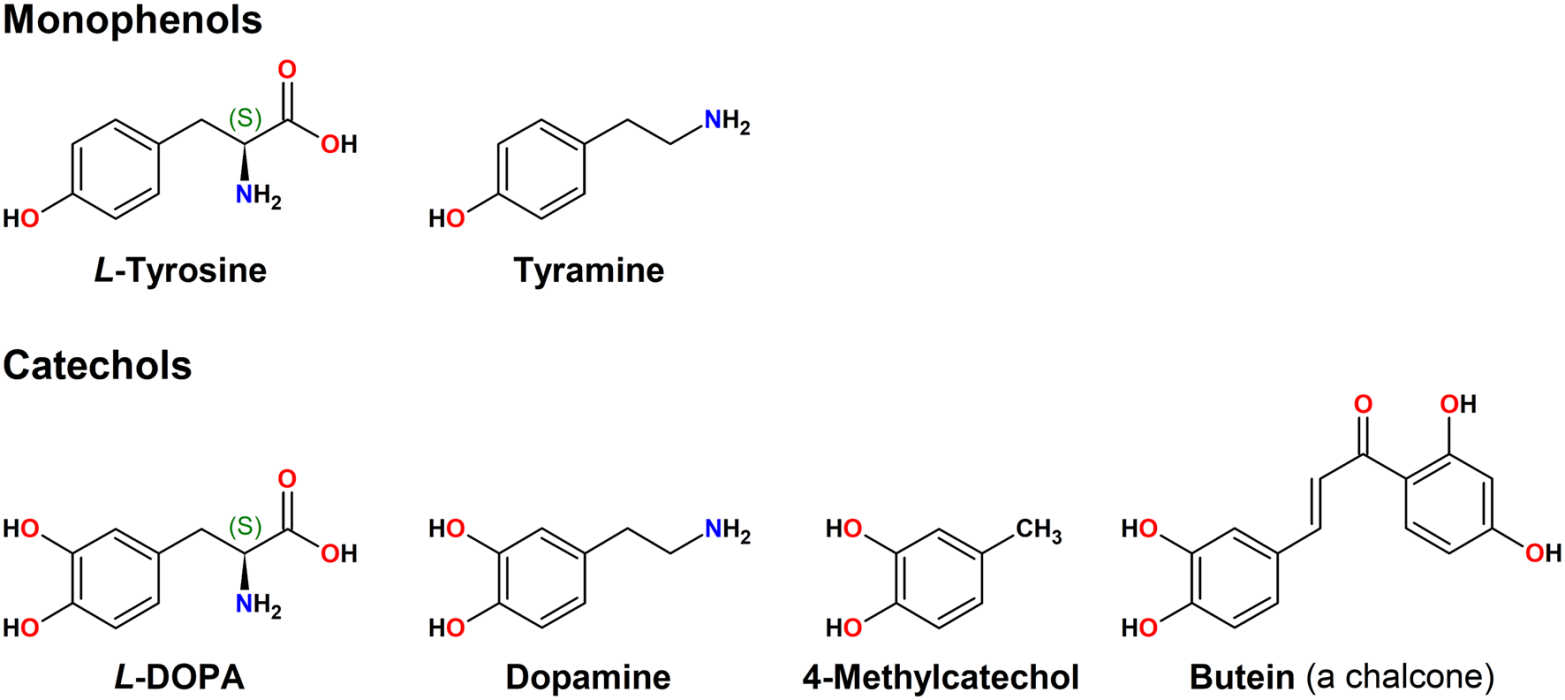
Structures of substrates used for testing the effect of amino acid exchanges on PPO activity. The proteinogenic amino acid l-tyrosine and its decarboxylation product tyramine are standard substrates for the determination of tyrosinase activity. Their corresponding catechols l-DOPA (l-3,4-dihydroxyphenylalanine) and dopamine represent the most common substrates for measuring the catechol oxidase activity of PPOs. Both *L*-tyrosine and *L*-DOPA contain an asymmetric carbon and are optically active. “*L*” (“left”) is used to indicate the stereoisomer according to the Fischer convention and “S” (from lat. *sinistrus*, meaning left) indicates the absolut configuration according to Cahn-Ingold-Prelog. 4-Methylcatechol is a popular substrate for plant PPOs as many of these enzymes oxidize 4-methylcatechol efficiently and the natural compound butein is commonly applied for PPOs involved in plant pigment formation

All percent values of activities given for mutant enzymes in this article are relative to the activity of the respective wild-type enzyme measured with the same substrate and under identical experimental conditions. When available, the values are calculated from the turnover number *k*_cat_ of the enzyme on the respective substrate. Alternatively, reaction rates of the mutant and wild-type enzyme on the same substrate and using identical concentrations of both enzyme and substrate are compared. Structures of the substrates used for activity measurements refered to in this article are shown in Fig. 11.

##### Copper-coordinating histidines

The six histidines (HisA_1–3_ and HisB_1–3_) that coordinate the two copper ions of the type III copper center [56] are perfectly conserved among all type III copper proteins. Five of the six copper-coordinating histidines are anchored on two of the four α-helices that make up the core of type III copper proteins while HisA_2_ sits on a loop (Fig. 4). For the fungal TYR *Ab*PPO3 from *Agaricus bisporus* the binding constant between the *apo*-enzyme (i.e., the enzyme without metal in the active center) and copper ions was measured at pH 6, with the result of 10^15^ M^−1^ for Cu(I) and 10^13^ M^−1^ for Cu(II) [102]. These values explain why removal of copper from its type III copper center requires extremely strong copper chelators like the cyanide anion ([C≡N]^−^, 10^16.8^ M^−1^ with Cu(I) at pH 6 [102]) while other strong chelators like ethylenediaminetetraacetic acid (EDTA; 10^12.2^ M^−1^ for Cu(II) [103]) are not efficient for removing copper from this metalloenzyme [85]. A number of (bacterial) TYRs are weaker chelators for copper ions, e.g., *Bm*TYR from *Bacillus megaterium* exhibited 20% inhibition with 0.1 mM EDTA and 100% inhibition with 1 mM sodium diethyldithiocarbamate [104].

Crystallographic data indicates that both copper ions are somewhat flexible and may move considerably from their standard positions. In crystal structures of both the bacterial TYR *Sc*MelC2 from *Streptomyces castaneoglobisporus* [77] and the fungal TYR *Ao*MelB from *Aspergillus oryzae* [105] both CuA and CuB were detected at different positions. The authors of both studies ascribe an essential role for the binding of an incoming substrate to the copper migration and suggest copper flexibility as a prerequisite of *ortho*-hydroxylation of incoming monophenols [77, 105]. The crystal structure of the fungal TYR *Ab*PPO4 from *Agaricus bisporus* shows an alternative position of CuB 2.3 Å removed from its standard location and stabilized by the 7th histidine [62]. CuB is interacting with four histidines and the conserved 7th histidine (Fig. 10) does participate in copper coordination. This 7th histidine is present in all structurally characterized PPOs, with the notable exception of the insect PPOs and the bacterial TYR *Pa*PvdP, which shares some architecture of the insect PPOs.

Mutation of each copper-coordinating histidine to either asparagine or glutamine in the bacterial TYR *Sg*MelC2 from *Streptomyces glaucescens* leads to complete abolishment of the enzymatic activity for replacement of HisA_1–3_ and HisB_1_ and almost complete abolishment of activity for the mutants targeting HisB_2–3_ (0.02% and 0.04% on l-DOPA) while the copper content remained at 50–90% [106]. Similar results were obtained for the fungal TYR *Ao*MelO from *Aspergillus oryzae* where replacing each of the six copper-coordinating histidines with asparagine led to complete loss of enzymatic activity and a copper content of around or slightly below 50% [107]. Additional confirmation of the essential nature of the six histidines of the type III copper center comes from the plant enzyme *Cg*AUS1 from *Coreopsis grandiflora* where replacing a CuA-coordinating histidine with alanine resulted in a reduction of the copper content by 50% to around one copper ion per protein chain and severely reduced (HisA_1_ to Ala: 0.5% of the wild-type’s activity on the chalcone butein) or abolished (HisA_2_ or HisA_3_ to Ala) the enzymatic activity of the enzyme [108]. Exchange of HisA_3_ of the bacterial enzyme *Sc*MelC2 from *Streptomyces castaneoglobisporus* to phenylalanine abolished the activity of the enzyme on l-tyrosine [80]. Mutation of a copper-coordination histidine severely impairs or abolishes the enzymatic activity of the mutant enzyme.

##### Thioether bridge

The thioether bridge (Fig. 9) is a posttranslational modification typical for tyrosinases of eukaryotic origin. The thioether bond between the γ-S of a neighboring cysteine and C2 in the imidazole ring of HisA_2_ was first observed in the fungal enzyme *Nc*TYR from *Neurospora crassa* [63]. HisA_2_ is the one amino acid among the six copper-coordinating histidines that is located on a loop rather than on a (more rigid) α-helix. The thioether bridge is formed after the active center has been loaded with copper in an intraenzymatic reaction that requires only molecular oxygen as the sole external cofactor [109]. Because formation of the thioether bridge is an intrinsic feature of type III copper centers, this modification is present in all type III copper proteins that feature a cysteine in a suitable position close to HisA_2_.

Thioether bridges are present in all structurally characterized fungal and plant PPOs (Table 1), with the notable exception of the short fungal PPOs (*Ao*CO4 and *Tt*PPO in Table 1). In the case of the bacterial TYRs, all biochemically characterized enzymes that do possess a C-terminal domain (Table 2) also possess a thioether bridge at their CuA site. *Pa*PvdP is the only exception known so far: This enzyme has the architecture of insect PPOs with two CuB sites and an N-terminal shielding domain. The three copper-coordinating histidines of CuB are all located on α-helices and consequently no rigidification of a copper-coordinating histidine is necessary. The known sequences of mammalian PPOs do not contain a suitably positioned cysteine, so no thioether bridge is expected in those enzymes. Hemocyanins of mollusks do contain a thioether bridge [110] while hemocyanins of arthropods do not [111].

It has been suggested that the *o*-hydroxylation of phenols would be blocked by the occurrence of the thioether bridge together with a bulky phenylalanine at the gate keeper position (see below) by disallowing substrate rotation in the active side, which was considered necessary for monophenol hydroxylation [101]. This theory, however, has been disproven by the crystal structure of walnut tyrosinase *Jr*TYR1 which is able to hydroxylate and oxidize monophenols despite possessing both structural features [68].

The thioether bridge plays a role in the suggested mechanism of copper-incorporation into the type III active site [109]. As there are a number of PPOs that lack a thioether bridge but do show enzymatic activity it is obvious that other mechanisms of copper-loading must exits.

Removal of the thioether bridge by mutation of the respective cysteine residue to alanine did result in a severe reduction (to ≈3% activity on l-tyrosine) of enzymatic activity of the fungal tyrosinase *Ao*MelB from *Aspergillus oryzae* [112] while the same mutation abolished the activity of the TYR *Ao*MelO from the same fungus [107]. Severely reduced catechol oxidase activity was also observed in the mutant enzymes of *Cg*AUS1 from *Coreopsis grandiflora* in which the thioether bridge-forming cysteine had been replaced by glycine, alanine, serine, asparagine, and aspartic acid, leading to 8%, 1%, 3%, 0.2%, and 0.3% activity on dopamine, respectively [113]. On the other hand, these same mutant enzymes of *Cg*AUS1 were able to hydroxylate and oxidize the monophenol tyramine, which the wild-type *Cg*AUS1 cannot [113]. Taken together the mutational studies targeting the thioether bridge of PPOs clearly show a pronounced influence of this structural feature on the enzymatic activity.

##### Gate keeper

The “gate keeper” residue is located at the entrance of the active site, slightly above the two copper ions (Fig. 9). It was first described in the crystal structure of the sweet potato (*Ipomoea batatas*) enzyme *Ib*CO [56], where this position is occupied by a phenylalanine. All known plant PPOs feature a phenylalanine as their gate keeper while PPOs from other taxonomical groups have a wider variety of usually smaller amino acids at this position (Fig. 10).

The bulkiness of the gate keeper amino acid was the most striking difference between the first crystal structures of a CO (*Ib*CO from *I. batatas* [56], PDB-ID 1BT3, published in 1998) and a TYR (*Sc*MelC2 from *Streptomyces castaneoglobisporus* [77], PDB-ID 1WXC, published in 2006). Consequently, the presence of a bulky gate keeper was postulated to prevent monophenolic substrates from binding to CuA and thereby preventing their *o*-hydroxylation [114]. Later crystal structures of the plant TYRs *Jr*PPO1 (walnut) [68], *Md*PPO1 (apple) [81], and *Sl*PPO1 (tomato) [115] refuted this theory. These three plant PPOs do possess a phenylalanine as their gate keeper and are highly active on the monophenolic substrates l-tyrosine and tyramine [68, 81, 115].

The effect of exchanging the amino acid at the gate keeper position is varied, with mainly decreased but also two instances of increased enzymatic activities reported. Replacement of the small amino acid valine at the gate keeper position in the bacterial enzyme *Bm*TYR from *Bacillus megaterium* by a bulky phenylalanine did increase the activity on the monophenol l-tyrosine fourfold and halved the reaction speed on the corresponding catechol l-DOPA while the smallest amino acid glycine as the gate keeper of *Bm*TYR resulted in an almost eightfold higher activity on l-tyrosine and also increased the activity on l-DOPA fourfold [116]. The exchange of the phenylalanine gate keeper of the apple TYR *Md*PPO1 to alanine severely reduced the activity of this enzyme on dopamine and completely abolished activity on the monophenol tyramine [117]. The activity was estimated by visually following the darkening of protein crystals soaked with a solution of the respective substrate and consequently no numerical values for the reduction in activity are reported. A similar picture was seen for the plant PPO *To*PPO2 from *Taraxacum officinale* (dandelion): mutation of the phenylalanine gate keeper to leucine reduced the enzymatic activity on 4-methylcatechol to 20% and the activity on tyramine was reduced to 24% while replacement of the *To*PPO2 gate keeper with either proline or glycine resulted in an enzymatically inactive enzyme [118]. For the plant enzyme *Jr*PPO1 from *Juglans regia* (walnut), replacing the phenylalanine gate keeper with glycine resulted in severely reduced enzymatic activity both on dopamine (8%) and tyramine (1%) [119]. In the case of the plant enzyme *Cg*AUS1 from *Coreopsis grandiflora*, mutation of the original phenylalanine at the gate keeper position to alanine, histidine, and aspartic acid caused almost complete abolishment of the enzyme’s activity on the catechol dopamine (0.16%, 0.13%, and 1.7% of the wild-type activity, respectively), while mutation to leucine only reduced the activity on dopamine to 20% of the wild-type activity and induced significant activity on the monophenol tyramine [113]. The mutation studies targeting the gate keeper position revealed the importance of the bulky phenylalanine in plant PPOs to guide incoming substrates towards the active site, likely through hydrophobic and π–π interactions with the aromatic ring of an incoming phenol [68, 108]. All mutants of plant enzymes that replaced the phenylalanine gate keeper with a smaller amino acid received a marked penalty on their enzymatic activity. On the other hand, the *Cg*AUS1 mutant with leucine at the gate keeper position—while weaker on catechols—did become a tyrosinase [113], indicating that a bulky gate keeper may indeed interfere with TYR activity.

##### Glutamic acid as the water keeper

As the name suggests, the water keeper residue keeps a “conserved water” molecule in place (Fig. 9). This water molecule is stabilized by the water keeper and the amino acid at the 1st activity controller position (see below). The position of the water keeper is held by an almost perfectly conserved glutamic acid in all characterized PPOs with the sole exception of some fungal short PPOs like *Ao*CO4 from *Aspergillus oryzae* in which the water keeper position is occupied by a glutamine [120].

It was proposed that it is the conserved water molecule that abstracts a proton from an incoming monophenol [101], that the water keeper itself deprotonates both monophenolic and catecholic substrates [70], and, more recently, that the basicity of the conserved water controls whether a PPO is restricted to catechols or is also able to process monophenols [121].

Mutation of the glutamic acid water keeper did invariably reduce the enzymatic activity of the altered PPO greatly. The exchange of the glutamic acid water keeper in the insect enzyme *Ag*PPO8 from *Anopheles gambiae* to a less acidic, uncharged glutamine caused a reduction of the enzymatic activity on the catechol dopamine to ≈7% of the wild-type activity while the reaction on the monophenol tyramine was slowed to ≈13% [70]. A different effect was reported for the plant enzyme *To*PPO2 from *Taraxacum officinale* (dandelion) which lost all enzymatic activity after its water keeper was changed to glutamine, but the enzyme with aspartic acid as its water keeper was affected only slightly and preserved two-thirds of the wild type’s activity on 4-methylcatechol [118]. Replacement of the acidic water keeper of the plant enzyme *Cg*AUS1 from *Coreopsis grandiflora* with a small and uncharged alanine reduced the enzymatic activity on dopamine to 0.5% of the wild-type activity and *Cg*AUS1 with a positively charged lysine at the water keeper position was enzymatically inactive [113]. The position of the negatively charged water keeper seems to be catalytically relevant as indicated by two double mutants of *Cg*AUS1 that replaced the water keeper with alanine and simultaneously introduced a glutamic acid at either the gate keeper or the 1st activity controller position (see below). Both double mutants displayed catalytic activities even lower (0.2% for both positions of glutamic acid) than the single mutant that replaced only the water keeper with alanine [113]. The mutagenesis studies carried out on the water keeper position of PPOs provide a strong indication for the need of a carefully positioned negative charge in the active center for catalytic activity of type III copper proteins.

##### 1st activity controller at HisB_1_ + 1

The 1st activity controller of a PPO is the amino acid immediately following the first copper-coordinating histidine of CuB, written HisB_1_ + 1 for short (Fig. 9). This amino acid is positioned at the entrance to the active site and it may interact with an incoming substrate. PPOs carry different amino acids at this position: TYRs usually have either a negatively charged (often asparagine or aspartic acid) or a small, uncharged (frequently glycine or alanine) amino acid at this position while enzymes classified as COs often feature a medium sized, uncharged amino acid (e.g., isoleucine or threonine) at HisB_1_ + 1.

A polar or acidic amino acid at HisB_1_ + 1 can—together with the glutamic acid water keeper—coordinate and activate a water molecule close to the copper ions which could then accept a proton from an incoming phenol (p*K*_a_ ≈10). An asparagine at the 1st activity controller position was proposed as the defining feature of TYRs [122], but a number of TYRs with different amino acids at HisB_1_ + 1 make that proposition untenable. Structurally characterized TYRs that feature an amino acid different from asparagine as their 1st activity controller are *Ab*PPO4 from *Agaricus bisporus* (aspartic acid) [62], *Ao*CO4 from *Aspergillus oryzae* (glycine) [123], *Pa*PvdP from *Pseudomonas aeruginosa* (aspartic acid) [124], *Md*PPO1 from *Malus domestica* (alanine) [81], and *Sl*PPO1 from *Solanum lycopersicum* (serine) [115].

For the 1st activity controller position the highest number of mutation studies is available. Exchange of the asgaragine at HisB_1_ + 1 in the bacterial TYR *Sg*MelC2 from *Streptomyces glaucescens* with glutamine resulted in almost complete loss of enzymatic activity (0.05% of the wild-type activity on the catechol l-DOPA) [106]. In the bacterial enzyme *Bm*TYR from *Bacillus megaterium*, mutation of the asparagine it carries at the HisB_1_ + 1 position to either alanine or aspartic acid reduced the enzymatic activity on l-DOPA to ≈45% and ≈7% and on the phenol l-tyrosine to ≈17% and ≈9% of the wild-type activity, respectively (all activities were measured in the presence of 10 µM CuSO_4_) [125]. The enzymatic activity of the plant enzyme *Vv*CO from *Vitis vinifera* (grapes) on both 4-methylcatechol and the phenol tyramine could be improved significantly by exchange of the original glycine at HisB_1_ + 1 to asparagine [122]. Since the activity of the wild-type and mutant *Vv*COs was gauged by staining of a protein band in an acrylamide gel, no numerical values for the activity increase are available. For the plant enzyme *Md*PPO1 from *Malus domestica* (apple), its alanine at HisB_1_ + 1 was replaced by threonine which caused a reduction of its enzymatic activity towards both the catechol dopamine and the phenol tyramine [117]. The activities were estimated by exposing protein crystals to solutions of the respective substrate and consequently also in this case no numerical values for the activity decrease were determined. Replacement of the glycine at HisB_1_ + 1 of the plant enzyme *To*PPO2 from *Taraxacum officinale* (dandelion) with either threonine or asparagine reduced the activity of this TYR towards dopamine to 7% of the wild type’s activity for both mutations and towards tyramine to 6% and 11%, respectively [118]. In the isoenzyme *To*PPO6 exchange of its threonine at HisB_1_ + 1 to glycine resulted in a 15% increase in the enzymatic activity towards dopamine while *To*PPO6 with asparagine at HisB_1_ + 1 retained only 11% of the wild type’s activity on dopamine [118]. Mutation of the original asparagine at HisB_1_ + 1 of the plant TYR *Jr*PPO1 from *Juglans regia* (walnut) to lysine caused a reduction to ≈3% on dopamine and to 0.2% on tyramine [119]. *Jr*PPO1 with a glycine at HisB_1_ + 1 showed a 220% increase in activity on dopamine while its activity on tyramine was reduced to 30% [126]. For the isoenzyme *Jr*PPO2, replacing its original glycine at HisB_1_ + 1 with asparagine caused a reduction of activity on dopamine to 35% while its activity on tyramine showed a 19% increase [126]. Nine mutants of the plant enzyme *Cg*AUS1 from *Coreopsis grandiflora* that replaced its original threonine at HisB_1_ + 1 were tested: *Cg*AUS1 with isoleucine or lysine at HisB_1_ + 1 suffered a severe loss of activity on dopamine to only ≈5% and ≈4%, respectively [113]. Asparagine and glutamic acid at HisB_1_ + 1 of *Cg*AUS1 increased the enzymatic activity on dopamine by 50% and 150%, respectively, and also induced activity towards tyramine [113]. Newly formed activity on tyramine was reported for aspartic acid, glycine, serine, cysteine, and alanine at HisB_1_ + 1 of *Cg*AUS1 while the activity of the mutant enzymes on dopamine was reduced to 95%, 61%, 90%, 56%, and 25%, respectively [113]. Experiments that exchanged the amino acid at the HisB_1_ + 1 position conclusively proved the strong effect of the 1st activity controller on the enzymatic activity of PPOs.

##### 2nd activity controller at HisB_2_ + 1

The amino acid at the position HisB_2_ + 1 is called the 2nd activity controller. It is located on the same α-helix that also carries the water keeper, HisB_1_, and the 1st activity controller (Fig. 9). The diversity of amino acids at HisB_2_ + 1 is greater that at the 1st activity controller position, with few positively charged amino acids present at this position in TYRs while the few PPOs annotated as COs are likely to feature a positively charged amino acid (arginine or lysine) at this position.

The 2nd activity controller was shown to influence the substrate selectivity of the two plant isoenzymes *To*PPO2 and *To*PPO6 from *Taraxacum officinale* (dandelion), and therefore the amino acid at HisB_2_ + 1 has also been dubbed the “substrate selector” [127]. Compared to the effect on the enzymatic activity of changes at the 1st activity controller position of a PPO (see above), the effects brought about by variation of the 2nd activity controller are smaller in general, but increased activity of the mutant enzymes is more frequently reported than for the 1st activity controller [98].

Exchange of the original arginine at HisB_2_ + 1 of the bacterial enzyme *Bm*TYR from *Bacillus megaterium* to histidine reduced the activity of the mutant enzyme on the catechol l-DOPA to 68% while its activity on the phenol l-tyrosine increased by 74% [128]. Mutation of the isoleucine at HisB_2_ + 1 of the plant enzyme *To*PPO2 from *Taraxacum officinale* (dandelion) to arginine caused a reduction of the activity on dopamine to ≈60% while replacement of the original arginine at position HisB_2_ + 1 in the isoenzyme *To*PPO6 with isoleucine increased the activity on dopamine by ≈70% [127]. For the plant TYR *Jr*PPO1, replacing its original leucine at HisB_2_ + 1 with arginine reduced the activity on both dopamine and tyramine to 27% and 7%, respectively [119]. For the plant enzyme *Cg*AUS1 from *Coreopsis grandiflora*, four mutants that replaced the original arginine at HisB_2_ + 1 were characterized. Glycine, leucine, and isoleucine at the HisB_2_ + 1 position of *Cg*AUS1 greatly increased the activity of the mutant enzymes on dopamine by 120%, 300%, and 200%, respectively [113]. *Cg*AUS1 with aspartic acid as its 2nd activity controller showed 150% increased activity on dopamine and was also active on tyramine (*k*_cat_ = 8.3 ± 0.5 s^−1^, well within the usual range for naturally occurring tyrosinases). Remarkably, the mutant of *Cg*AUS1 with aspartic acid at HisB_2_ + 1 exhibited this strong activity despite its very low copper content of 0.22 ± 0.02 copper ions per protein chain [113]. Taken together, the mutation studies targeting the amino acid at the HisB_2_ + 1 position unequivocally showed the pronounced effect of the 2nd activity controller on the enzymatic activity of PPOs.

#### Experimental structures of PPOs

To date (September 2024), experimental structures of 23 different PPOs have been published (Table 1). For all of those, the applied experimental method was X-ray crystallography with synchrotron radiation diffracted by single crystals grown in a vapor-diffusion setup. Eight of those 23 PPOs were isolated from their respective natural source (four plants, two insects, and two PPOs from the same fungi) while 17 (eight bacterial, four fungal, three plant, and two insect PPOs) were produced by heterologous expression in either the bacterium *Escherichia coli* (15 PPOs), the filamentous fungi *Trichoderma reseei* (1 PPO), or the yeast *Komagataella pastoris* (1 PPO). The numbers do not add up to the expected total as the structures of two enzymes were determined for both the enzyme isolated from the natural source and protein obtained by heterologous production (aurone synthase 1 of large-flowered tickseed, *Cg*AUS1: from petals and recombinant *E. coli*; and mushroom tyrosinase isoform 4 of button mushroom, *Ab*PPO4: from the stems of fruiting bodies and recombinant *E. coli*).

**Table 1:** Comprehensive list of PPOs with experimental crystal structures

Enzyme	Organism	I/R^a^	Domains^b^	PDB^c^	*R*/Å	Date^d^	References
*Ib*CO	*Ipomoea batatas*	I	T	1BT3	2.50	15.08.1997	[56]
*Vv*CO	*Vitis vinifera*	I	T	2P3X	2.20	19.06.2006	[129]
*Ms*PPO	*Manduca sexta*	I	L–T	3HHS	1.97	02.04.2008	[130]
*Ab*PPO3	*Agaricus bisporus*	I	T	2Y9W	2.30	02.11.2008	[99]
*Bm*TYR	*Bacillus megaterium*	R	T	3NM8	2.00	08.05.2009	[131]
*Sc*MelC2	*Streptomyces castaneoglobisporus*	R	T^e^	3AWU	1.16	14.12.2010	[78]
*Ao*MelB	*Aspergillus oryzae*	R	T–L	3W6W	1.39	07.12.2011	[132]
*Cg*AUS1	*Coreopsis grandiflora*	I	T–L	4Z11	2.50	09.11.2012	[8]
*Cg*AUS1	*Coreopsis grandiflora*	I	T	4Z0Y	1.60	20.11.2012	[8]
*Ag*PPO8	*Anopheles gambiae*	R	L–T	4YZW	2.60	06.12.2012	[70]
*Mj*PPOβ	*Marsupenaeus japonicus*	I	L–T	3WKY	1.80	24.01.2013	[133]
*Jr*PPO1	*Juglans regia*	I	T	5CE9	1.80	31.01.2013	[68]
*Cg*AUS1	*Coreopsis grandiflora*	R	T–L	4Z12	1.85	07.04.2013	[8]
*Ab*PPO4	*Agaricus bisporus*	I	T–L	4OUA	2.76	15.05.2013	[62]
*Ab*PPO4	*Agaricus bisporus*	I	T	4OUA	2.76	15.05.2013	[62]
*Ab*PPO4	*Agaricus bisporus*	R	T–L	5M6B	3.25	03.07.2015	[82]
*Ao*CO4	*Aspergillus oryzae*	R^f^	T	5OR3	1.79	22.02.2016	[123]
*Sl*PPO1	*Solanum lycopersicum*	R	T–L	6HQJ	1.80	25.02.2016	[115]
*Pa*PvdP	*Pseudomonas aeruginosa*	R	L–T	6EYS	2.09	26.07.2016	[124]
*Md*PPO1	*Malus domestica*	R	T–L	6ELS	1.35	26.09.2016	[81]
*Bt*TYR	*Burkholderia thailandensis*	R	T–L	5ZRD	2.30	23.07.2017	[134]
*Sa*MelC2	*Streptomyces avermitilis*	R	T^e^	6J2U	1.30	18.12.2018	–^g^
*Rs*TYR	*Ralstonia solanacearum*	R	T–L	7XIO	2.64	07.06.2019	–^g^
*Tt*PPO^h^	*Thermothelomyces thermophilus*	R^i^	T	6Z1S	1.53	27.08.2019	[135]
*Vs*TYR	*Verrucomicrobium spinosum*	R	T	8BBQ	1.43	26.11.2021	[136]
*Hc*TYR1	*Hahella* species	R	T–L	8B74	1.97	29.04.2022	–^g^
*Aa*PPO6	*Aedes aegypti*	R	L–T	8JIB	3.15	19.11.2022	–^g^

^a^*I* isolated from the natural source, *R* recombinantly produced in *Escherichia coli* (all PPOs except *Ao*CO4 and *Tt*PPO)

^b^The central tyrosinase domain (*T*) contains the type III copper center and is common to all PPOs. PPOs containing only this domain are commonly referred to as “active” PPOs. Eukaryotic PPOs often contain an additional lid-like domain (*L*) that covers the active site and is usually found at the C-terminal side of the tyrosinase domain. The presence of this domain hinders substrate access to the active site which is why these PPOs are often labelled “latent” enzymes. In insect PPOs the lid-like domain is located N-terminal to the central tyrosinase domain, its function is not changed by the translocation. The bacterial enzyme *Pa*PvdP has structural features similar to an insect PPO: Two CuB-motifs instead of CuA and CuB as well as the presence of a N-terminal domain that does, however, not cover the active site

^c^For each enzyme with more than one entry, the structure with the best resolution is reported here

^d^Day of data collection (X-ray diffraction experiment) for the cited PDB entry

^e^These enzymes were crystallized in complex with their respective caddie protein *Sx*MelC1

^f^The extracellular “short tyrosinase” *Ao*CO4 was expressed in the filamentous fungi *Trichoderma reesei* RUT C-30

^g^No publication linked to the PDB-entry (“to be published”)

^h^Mutant G292N (changed 1st activity controller; for this particular mutation, higher tyrosinase activity is predicted)

^i^The fungal *Tt*PPO was produced in the yeast *Komagataella pastoris* (older name *Pichia pastoris*)

For 24 out of the 27 entries in Table 1 single crystals were grown from a solution containing polyethylene glycols (PEGs) with average molecular weights in the range from 1 to 20 kDa. The three remaining crystallization conditions employed alcohol (*Bt*TYR: ethanol, isopropyl alcohol, and methanol), a mixed polyethylene/polypropylene glycol with primary amines as the end groups (specifically Jeffamine ED-2001 for crystallization of *Mj*PPOβ), or high salt concentrations (*Rs*TYR: 1.7 M ammonium sulfate).

For the plant enzyme *Cg*AUS1, the crystal structure generated with the recombinant enzyme (PDB 4Z12: 1.85 Å) has a superior resolution than the structure generated with protein isolated from the natural source (PDB 4Z11: 2.50 Å). In the case of the fungal enzyme *Ab*PPO4 the situation is reversed; here the structure derived from the enzyme isolated from the natural source (PDB 4OUA: 2.76 Å) features a better resolution than the crystal structure generated with the recombinant enzyme (PDB 5M6B: 3.25 Å). In both cases the protein crystal leading to the structures with superior resolution (4Z12 and 4OUA) were grown in the presence of the tellurium-centered Anderson–Evans polyoxotungstate Na_6_[TeW_6_O_24_] (1 mM in both cases) that helped to rigidify more flexible regions of the proteins and also provided bridges between the crystals that could not have been formed by the protein chains alone [137, 138].

### Catalytic cycle of TYR

**Scheme 5:**
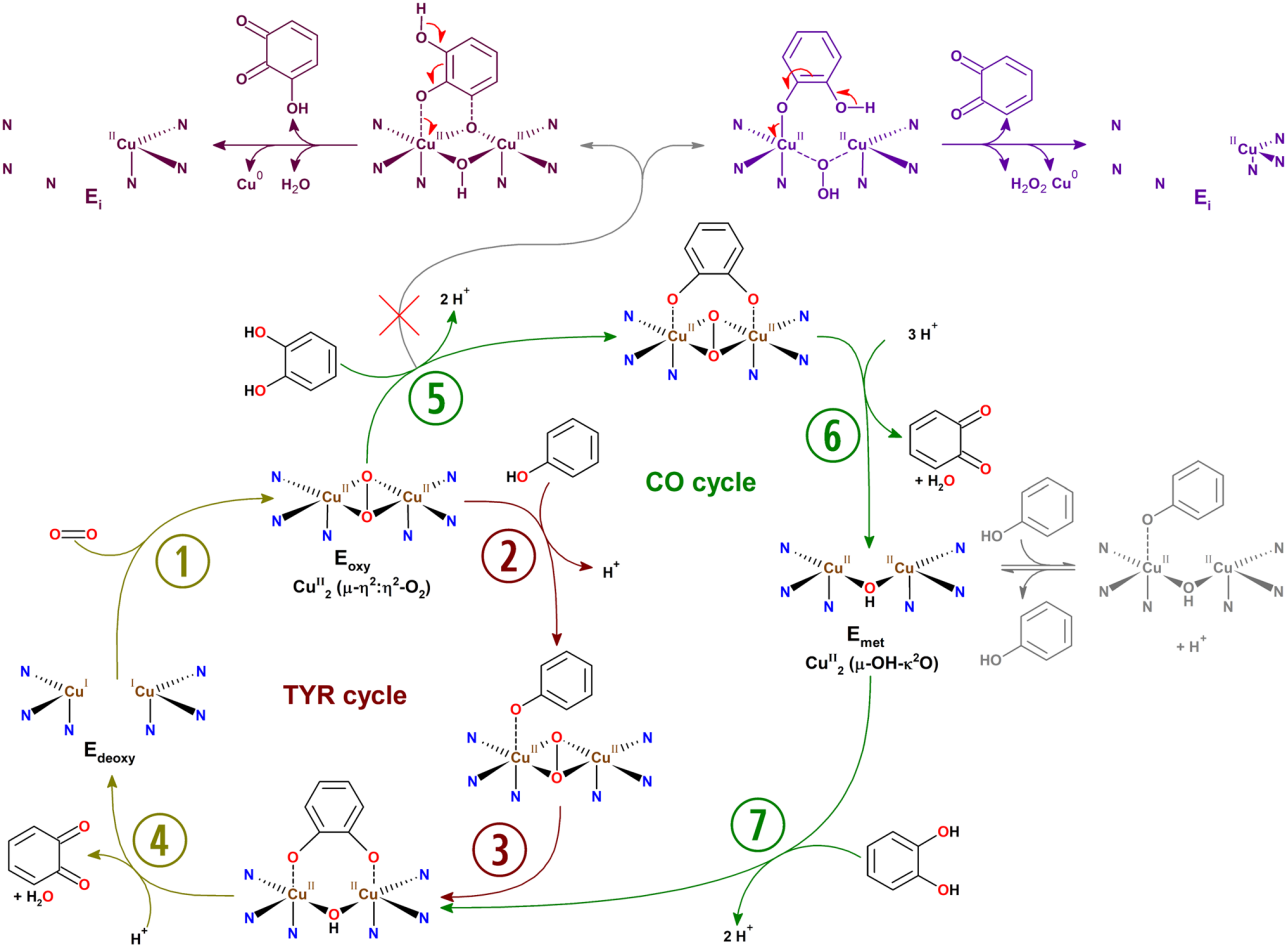
Catalytic cycles of tyrosinase acting on monophenols (TYR cycle) or catechols (CO cycle) [15]. The top row shows the suicide inactivation pathway (see “Suicide inactivation”) that is triggered by a catechol being processed as a monophenol. The model on the left in raspberry color depicts the Quintox mechanism of tyrosinase inactivation [140], for which the characteristic trihydroxylated products were detected in reaction solutions. The “Murcia model” for tyrosinase suicide inactivation [141] based on kinetic considerations is shown on the right in violet. Unproductive binding of monophenols to E_met_ [142] is represented in gray on the right. Note that the charges on copper-coordinated oxygen species (peroxide ion, O^−^–O^−^; hydroxide ion, OH^−^; and deprotonated hydroxy groups of the substrate, phenyl-O^−^) are not shown explicitly in order to make the scheme easier to read

The catalytic cycle of tyrosinase (Scheme 5) begins with binding of molecular oxygen to the deoxy form of the enzyme (E_deoxy_, Cu^I^–Cu^I^) [139] in the characteristic side-on (µ-η^2^: η^2^) binding mode [57] which generates the oxy form (E_oxy_, Cu^II^–µ-O_2_–Cu^II^) that is catalytically competent towards both monophenolic and catecholic substrates [15]. E_oxy_ can hydroxylate and oxidize incoming monophenolic substrates, yielding the corresponding *o*-quinone and water (TYR activity: ①②③④) or just affect the two-electron oxidation of a catecholic substrate to the respective *o*-quinone (CO activity: ①⑤⑥⑦④).

In the case of a monophenolic substrate, processing of a single phenolic molecule transfers the enzyme to the E_deoxy_ state, thereby closing the catalytic cycle. For a catecholic substrate, E_oxy_ first oxidizes the substrate and transitions into the met form (E_met_, Cu^II^–µ-OH–Cu^II^), which may bind and oxidize another catechol molecule but is catalytically inactive towards monophenols. Oxidation of the second catechol again leads to E_deoxy_ and with the binding of a new molecule of dioxygen the next catalytic cycle is set to start. With every molecule of its cofactor dioxygen, TYRs can hydroxylate and/or oxidize either one phenol or two catechols. The reaction products are in both cases *o*-quinone and one molecule of water per *o*-quinone released. In contrast, the multi-copper oxidase laccase (EC 1.10.3.2) oxidizes four substrate molecules (by single electron abstraction) per dioxygen that gets reduced to water in the process [61] (Scheme 1C).

As evidenced by a series of crystal structures, the Cu–Cu distance varies with the oxidation state of the copper ions and the bridging ligand. In E_deoxy_ the cuprous ions are furthest away from each other with intermetal distances of 4.1–4.6 Å, while the cupric ions in E_met_ are spaced at 3.2–4.0 Å and in the E_oxy_ form the two cupric ions come to within 2.8–3.2 Å of each other [97].

#### Lag phase

Enzymatic reactions do not usually proceed at the full reaction speed immediately after substrate and enzyme have been brought into contact. This is due to the finite time it takes for all present catalyst particles to attain their catalytically active form (which is often markedly different from the catalyst’s resting form) and also mass transport limitations slow the reaction down. In the case of enzymes, the protein catalysts may need to bind cofactors, change their folding to accommodate binding of the substrate, or adjust their redox state to allow catalysis. The term lag phase refers to the time between the initial contact of the reaction partners and the attainment of the final full reaction speed of the enzymatic reaction.

The oxidation of catechols by PPOs proceeds almost immediately at the steady state rate, while for monophenols a distinctive period of slowly increasing reaction rate is generally observed—the lag phase. Only the oxy form of the enzyme is competent for the hydroxylation and oxidation of monophenolic substrates while the part of the enzyme that resides as E_met_ cannot participate in the TYR cycle (Scheme 5).

Depending on the enzyme in question and the specific isolation and storage conditions the exact values vary, but in most cases only a small percentage of a given TYR preparation will be present in the E_oxy_ form while the majority is in the E_met_ form. E_met_ cannot enter the TYR cycle but it can be recruited into it either via the oxidation of a catecholic substrate (Scheme 5, ⑦), by the addition of exogenous reductants like dithiothreitol (DTT) [143], or forced formation of the oxy-form by hydrogen peroxide (H_2_O_2_) [144]. The reduction with agents like DTT likely produces E_deoxy_, while treatment with H_2_O_2_ leads to the formation of E_oxy_. Both forms are part of the TYR cycle and therefore both reduction or oxidation work in abolishing the lag phase. If neither reductant nor peroxide source is added, the recruitment of enzyme currently in the E_met_ form depends on the availability of traces of the catecholic form of the substrate. This is often provided by non-enzymatic generation of catecholic moieties, e.g., by the redox exchange between dopaquinone and leucodopachrome (Scheme 2) in the reaction on tyrosine. For monophenolic substrates that do not generate catechols by redox exchange with their respective TYR-generated *o*-quinones, like e.g., 4-*tert*-butylphenol [82, 144], the addition of either reducing agent or H_2_O_2_ is necessary in order to achieve appreciable enzymatic turnover.

The lag phase is reported to be longer for substrates that bind tighter to the enzyme and/or are used at higher concentrations [145], which indicates that non-productive binding of the phenolic substrate to E_met_ (right in Scheme 5) contributes to the phenomenon of the lag-phase.

#### Suicide inactivation

Self-poisoning of a catalyst refers to the irreversible inactivation of a catalyst due to its reaction with a substrate or substrate-like molecule. For enzymes, the term suicide inactivation is used for this phenomenon to highlight that the inactivating species is formed by an action of the enzyme itself. Common mechanisms of irreversible activity loss by suicide inactivation are covalent modifications of active site residues, blocking of substrate entry or product exit channels, or loss of essential cofactors.

Erroneous binding of certain catecholic substrates (e.g., catechol itself) to E_oxy_ can trigger the suicide inactivation pathway leading to loss of copper [146] from the active site which consequently inactivates the enzyme. To explain this phenomenon two models have been proposed that both assume a monophenol-like processing of a catecholic substrate [140, 141] (Scheme 5, top row). Both models postulate the reduction of a copper ion in the active center to the metallic state, which leads to loss of the metal cofactor and consequently inactivates the enzyme.

However, neither of these two proposed mechanisms includes any alterations to the copper-coordinating histidines and so cannot account for the reported (putatively hydroxyl radical-mediated) covalent modification of the third copper-B-binding histidine of *Neurospora crassa* tyrosinase during its suicide inactivation on catechol [146].

For two fungal tyrosinases the relative rates of substrate oxidation and suicide inactivation on catechol have been determined: tyrosinase from *N. crassa* managed approximately 5000 oxidations per inactivation event [146] while the values reported for preparations containing *Agaricus bisporus* tyrosinases are quite dissimilar with ≈69 [140] and 100,000 [147] *o*-quinones formed per inactivation event. The striking disagreement between these two measurements may be explained by the different experimental conditions applied: in the experiment that yielded far more *o*-quinones per inactivation event chromatographically purified tyrosinase was applied while the other experiment utilized a crude mushroom extract that likely also contained a number of other redox-active enzymes [148, 149]. The catalytic rate (*k*_cat_ value) on catechol of the chromatographically purified *A. bisporus* tyrosinase was 13.4 ± 0.8 times higher than the value determined for the crude protein extract, hinting at a substantial level of contaminants in the latter.

#### Reaction mechanism of TYR

**Scheme 6:**
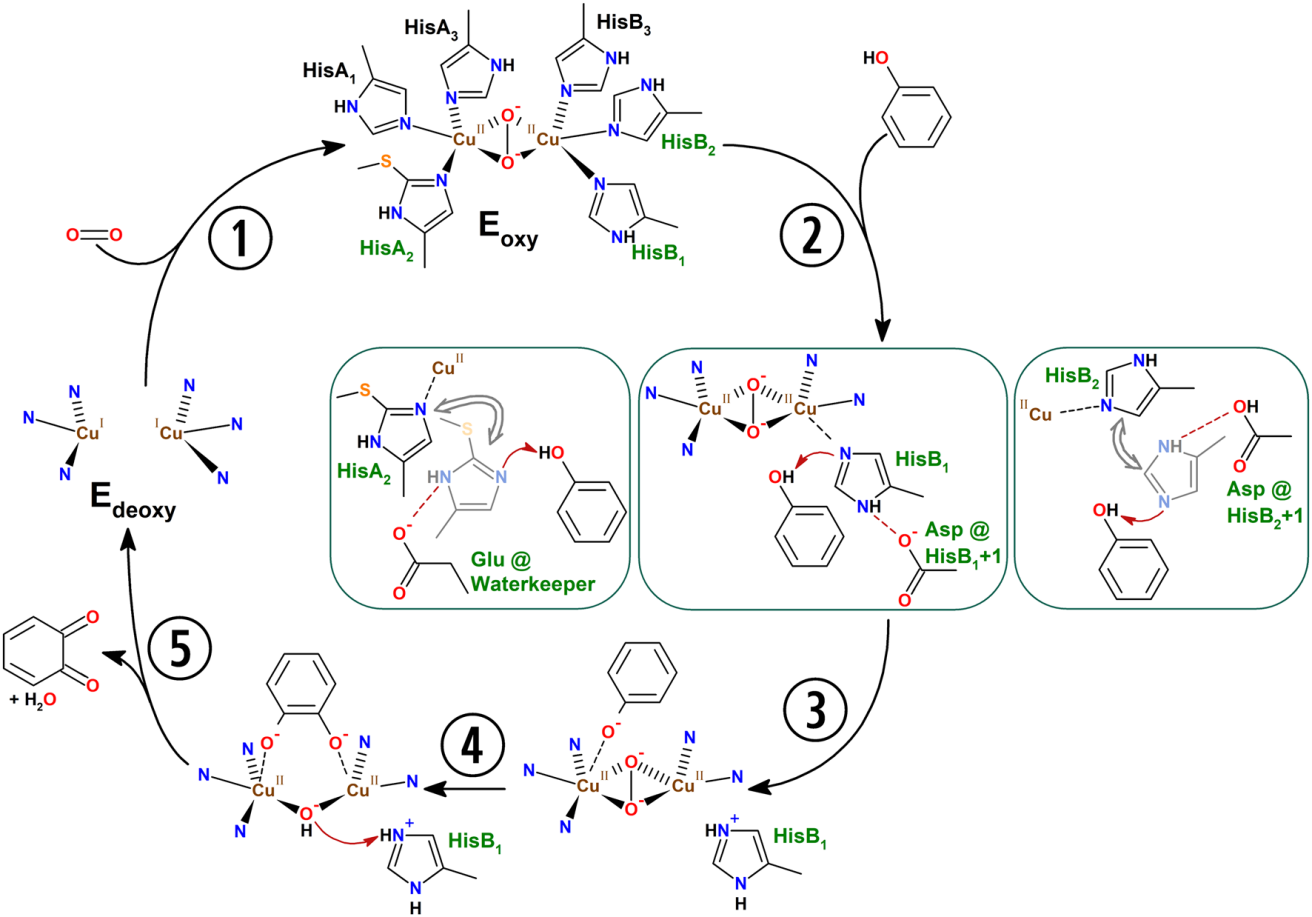
Mechanism of TYR acting on monophenols according to Kampatsikas et al. [113]. ① The catalytic cycle begins with binding of molecular oxygen to the reduced form of the type III copper center, which results in the oxy-form of the enzyme (E_oxy_). The copper-coordinating histidines implicated for substrate deprotonation are marked with green labels while the three histidines at the back of the active site groove have black labels. ② Incoming phenolic substrates are deprotonated by copper-coordinating histidines which have their basicity enhanced by amino acid residues close to the active center: shown are acidic amino acids next to the first two histidines of the CuB site (1st and 2nd activity controllers) and the highly conserved glutamic acid (water keeper) at the entrance to the active site that coordinates a water molecule in all crystal structures of PPOs that have sufficient resolution for the localization of single oxygen molecules. ③ Coordination of the deprotonated substrate (phenolate) to CuA allows its ④ *ortho*-hydroxylation in an electrophilic aromatic substitution reaction that results in the catecholic intermediate still bound to the active center. ⑤ Two-electron oxidation of the coordinated catechol yields the final *o*-quinone product and reduces the copper center to its deoxy form (E_deoxy_), thereby closing the catalytic cycle. The proton abstracted from the substrate combines with the bridging hydroxy group to form water that leaves the active center as well

The mechanistically most demanding and also the slowest step in the two interweaved catalytic cycles of tyrosinase is the *o*-hydroxylation of the monophenolic substrate [150]. On the basis of the experimentally observed slopes in Hammett plots^2^ this hydroxylation proceeds via an electrophilic aromatic substitution [152] and it seems to be dependent on prior deprotonation of the monophenol [153]. This deprotonation step would arguably necessitate the presence of a catalytic base within the enzyme. The currently most widely accepted mechanism locates this functionality in the conserved histidines that coordinate to the copper ions of the active center (Scheme 6) [98].

If TYRs and COs do indeed differ biochemically, the phenol monooxygenation is the step where the difference would manifest itself. To date, no TYR devoid of CO activity is known and on the basis of the best current understanding of the reactivity of type III copper centers, such an enzymatic activity would be incompatible with copper biochemistry. From an application point of view this is somewhat disappointing as such an enzyme would be quite useful for the preparation of a potentially unlimited numbers of catecholic substances. While the addition of a reducing agent like ascorbic acid can be applied to generate a system that yields tyrosinase-generated catechols by reduction of the enzymatically generated *o*-quinones, the biotechnological application of reductants in TYR-catalyzed reactions is hampered not only by considerations concerning (molar) efficiency but also by the detrimental effect those agents have on the stability of the employed oxidases [154].

A number of hypotheses have been forwarded to explain the perceived biochemical differences between TYRs and COs by structural factors unique to either enzyme class, but so far every proposed model of discrimination conflicted with newer (and sometimes also with older) experimental data [97]. One possible explanation for this lack of success in finding a structural discriminator between TYRs and COs is that these differences simply do not exist, in short that TYR = CO.

### Tyrosinases of *Agaricus bisporus*

A crude enzyme preparation from *A. bisporus* fruiting bodies that is sold as “mushroom tyrosinase” provides the majority of tyrosinase activity for basic research studies on this enzyme family and for most of the applied research utilizing tyrosinases. While it is certainly a useful source of tyrosinase that was, e.g., utilized for the first crystal structure of a fungal PPO [99], it is regrettably sometimes also applied as delivered (i.e., without further purification) for experiments that require pure protein. This inevitably leads to wasted effort, money, and time or even introduces misleading results into the scientific literature—often without the authors’ acknowledging the problem. The manufacturers make no claim whatsoever that “mushroom tyrosinase” is a pure protein and there are reports available that specifically deal with the numerous contaminants found in those preparations [148, 149].

The yield of pure TYR from *A. bisporus* fruiting bodies is quite low, e.g., 2 mg of *Ab*PPO4 was purified from 300 g of lyophilized stipes (corresponding to approximately 10 kg of fresh mushrooms since the caps were not used) [95]. This is mainly because of the extensive pre-treatment (removal of phenolics and other interfering substances) which is necessary to generate a stable extract that can be further purified chromatographically.

*A. bisporus* has six genes coding for PPOs (*ab*PPO1-6) that are located on chromosomes V and VIII [155]. Two of these six genes, namely the ones coding for *Ab*PPO3 and *Ab*PPO4, are transcribed circa 100-times more frequently than any of the other four [155] and thus these two enzymes presumably make up the overwhelming majority of PPOs in *A. bisporus*.

The variety *bisporus* accounts for virtually all the commercially grown button mushrooms and does contain the unmixed genetic material of both its parental strains in all its tissues except the spore-bearing cells [156]. With respect to the PPOs of *A. bisporus*, this means that the expected number of isoenzymes is 12. The parental strains of the commercial hybrid “Horst U1” are *A. bisporus* var. *bisporus* H39 and *A. bisporus* var. *bisporus* H97. High-quality genomes are available for both strains [156]. Isoenzyme pairs from these two strains are very similar to each other with identities on the amino acid level between 96.7% to 99.5% while the identities between the isoenzymes are as low as 33.5% [85]. The few differences in the same isoenzyme between the enzymes of these two strains are not in positions previously indicated as important to the enzymatic activity so the effective number of PPO isoenzymes in *A. bisporus* can be assumed as six.

Cloning of the first two of the respective genes (*ab*PPO1 and *ab*PPO2) was first reported in 2003 [157] but the accompanying heterologous expression attempt using *E. coli* as the host yielded only inactive protein. Genes for *Ab*PPO3 and *Ab*PPO4 were cloned in 2010 [158], but again the expression in *E. coli* yielded no enzymatically active protein. The crystal structure of the central tyrosinase domain of *Ab*PPO3 in complex with the lectin-like protein *Ab*MtaL (PDB 2Y9W, 2Y9X) was reported in 2011 [99] and represented a crucial step towards understanding these enzymes.

In 2012 the first successful recombinant production of a “mushroom tyrosinase” was reported [159]. It used *Saccharomyces cerevisiae* as the expression host and produced approximately 0.5 mg *Ab*PPO2 per liter of culture medium. Purification of *Ab*PPO4 from *A. bisporus* was reported in 2014 [95], followed shortly by the crystal structure of both the central tyrosinase domain alone and the latent form still containing the C-terminal domain of *Ab*PPO4 (PDB 4OUA) [62].

High-yield production of recombinant *Ab*PPO4 in *E. coli* was reported in 2017 [82]. The yield was 140 mg *Ab*PPO4 per liter of bacterial culture and the recombinant enzyme was also crystallized (PDB 5M6B) which revealed no significant differences in the protein’s folding compared to the structure from 2014. *Ab*PPO3 and *Ab*PPO4 were subjected to an expression attempt again in 2019 [160], which failed for *Ab*PPO3 and produced an unreported yield of *Ab*PPO4. Production of the complete PPO family of *A. bisporus* var. *bisporus* in *E. coli* was reported in 2024 [85]. All six isoenzymes accept l-tyrosine and tyramine as substrate, which classifies them as TYRs. The most striking difference between the six TYRs of *A. bisporus* uncovered in this study pertains to acceptance of an abundant phenolic substance in *A. bisporus*: *N*-(4-hydroxyphenyl)-l-glutamine (HPG, also called γ-l-glutaminyl-4-hydroxybenzene) [161]. HPG is processed very rapidly by *Ab*PPO4 and is likely to be its physiological substrate while it is not a substrate at all for *Ab*PPO3.

### Production of recombinant PPOs

Except for PPOs from *Streptomyces* species (the largest genus of Actinobacteria which is named for its hyphal-like growth morphology), which “outsourced” the function of the C-terminal domain to another protein within the same operon, PPOs are usually expressed as latent enzymes that may also contain an N-terminal signal sequence responsible for their intracellular localization or secretion from the cell [66]. A notable exception to this general rule is found in the class of fungal “short tyrosinases”, which lack the C-terminal domain already at the genetic level and are generally extracellular enzymes [71]. For PPOs possessing a C-terminal domain the latter was long believed to be necessary for correct folding of the enzymatically active domain [162] as all attempts of expression using truncated versions of the respective PPO-coding genes without the C-terminal domain invariably resulted in either insoluble or at least enzymatically inactive proteins.

The first functional expression of a eukaryotic PPO without its C-terminal domain was reported only recently for grape PPO produced in *E. coli* [163]. While this experiment clearly shows that the C-terminal domain is not an absolute prerequisite for successful folding of the enzymatically active domain connected to it, the report of the heterologous production of active grape PPO remains, for the time being, the only one of its kind. For prokaryotic tyrosinases possessing a C-terminal domain the situation regarding expression of the tyrosinase domain alone is only slightly more consolidated: there are reports on the successful production of only the enzymatically active domain of the bacterial tyrosinase from *Verrucomicrobium spinosum* (“core tyrosinase”) [164] and the archaeal tyrosinase from *Candidatus Nitrosopumilus koreensis* (“mTyr-CNK”) [165], also with *E. coli* as the expression host in both cases.

Starting with the bacterial tyrosinase *San*MelC2 from *Streptomyces antibioticus* in 1983 [166], heterologous expression and/or homologous overexpression of PPO-encoding genes has been reported to date for 123 PPOs from 76 different species (2 archaea, 4 insects, 7 other animals, 10 fungi, 24 bacteria, and 29 plants; see Table 2). Among those, the majority were produced in the bacterium *E. coli* (106 of 156 PPO/expression host combinations), 10 PPOs were (also) produced in other bacteria (*Aeromonas media*, *Bacillus thuringiensis*, *B. subtilis*, *Komagataeibacter rhaeticus*, *Pseudomonas fluorescens*, *Ralstonia solanacearum*, *Streptomyces antibioticus*, *S. avermitilis*, *S. glaucescens*, *S. coelicolor*, *S. lividans*, *S. kathirae*, and *Vibrio natriegens*), and 31 PPOs were (also) expressed in eukaryotic cells (11 in fungi, 11 in plants, 6 in insects, and 3 in primate cells) . Use of two different expression hosts was reported for 19 PPOs, three enzymes (*Ao*MelO from *Aspergillus oryzae*, *Sa*MelC2 from *Streptomyces avermitilis*, and *Sg*MelC2 from *Streptomyces glaucescens*) were expressed in three different hosts, and four different hosts were used for production of another three PPOs (four different bacteria each for *Streptomyces antibioticus San*MelC2 [74, 166–168] and *Bacillus megaterium Bm*TYR [104, 169–171], as well as one bacterial, two insect, and one human expression host for *Homo sapiens Hs*TYR [172–175]).

**Table 2:** Wild-type PPOs: recombinant production attempts and basic biochemical characteristics

PPO	Source organism	Group^a^	Accession^b^	Sig^c^	LD^d^	Act^e^	References
*Hl*TYR1	*Haliplanella luciae*	Animalia	–	Yes	C	TYR	[176]
*Hs*TYR	*Homo sapiens*	Animalia	P14679	Yes	–	TYR	[172]
*Lv*PPO	*Litopenaeus vannamei*	Animalia	A5JSX2	–	C	TYR	[177]
*Mg*CO	*Mytilus galloprovincialis*	Animalia	A0A6B7E5P4	Yes	–	n.r.	[178]
*Mm*TYR	*Mus musculus*	Animalia	P11344	Yes	–	TYR	[179]
*Re*TYR	*Rana esculenta*	Animalia	Q0MVP0	Yes	–	TYR	[180]
*Sj*TYR1	*Schistosoma japonicum*	Animalia	Q5DB10	Yes	–	n.r.	[181]
*Sj*TYR2	*Schistosoma japonicum*	Animalia	Q5DHS0	Yes	–	n.r.	[181]
*Nk*TYR	*Nitrosopumilus koreensis*	Archaea	K0B2W2	–	–	TYR	[182]
*Ns*TYR	*Nitrosopumilus salaria*	Archaea	I3D139	–	–	TYR	[183]
*Am*TyrA	*Aeromonas media*	Bacteria	B2Z3P7	Yes	–	TYR	[184]
*Ba*TYR	*Bacillus aryabhattai*	Bacteria	A0A6H1TJ97	–	–	TYR	[185]
*Bc*Mel	*Bacillus cereus*	Bacteria	Q74NN3	–	–	TYR	[186]
*Bc*TYR	*Burkholderia cepacia*	Bacteria	SEU13261*	–	C	TYR	[187]
*Bm*TYR	*Bacillus megaterium*	Bacteria	B2ZB02	–	–	TYR	[188]
*Bt*Mel	*Bacillus thuringiensis*	Bacteria	Q6SS53	–	–	TYR	[189]
*Bt*TYR	*Burkholderia thailandensis*	Bacteria	Q2T7K1	–	C	TYR	[134]
*Cs*TYR	*Citreicella* sp. SE45	Bacteria	EEX16244*	–	C	CO	[190]
*Mm*PPOA	*Marinomonas mediterranea*	Bacteria	–	Yes	C	TYR	[191]
*Pa*PvdP	*Pseudomonas aeruginosa*	Bacteria	A0A0H2ZBG1	Yes	–	TYR	[25]
*Pf*PvdP	*Pseudomonas fluorescens*	Bacteria	–	Yes	–	n.r.	[192]
*Re*MelA	*Rhizobium etli*	Bacteria	Q8KIL0	–	C	TYR	[193]
*Rm*MepA	*Rhizobium meliloti*	Bacteria	P33180	–	C	TYR	[194]
*Rs*TYR	*Ralstonia solanacearum*	Bacteria	Q8Y2J8	Yes	C	TYR	[195]
*Sa*MelC2	*Streptomyces avermitilis*	Bacteria	Q79ZK1	–	–	TYR	[196]
*San*MelC2	*Streptomyces antibioticus*	Bacteria	P07524	–	–	TYR	[74]
*Sc*MelC2	*Streptomyces castaneoglobisporus*	Bacteria	Q83WS2	–	–	TYR	[197]
*Sg*GriF	*Streptomyces griseus*	Bacteria	B1VTI5	–	–	TYR	[26]
*Sg*MelC2	*Streptomyces glaucescens*	Bacteria	–	–	–	CO	[198]
*Sk*MelC2	*Streptomyces kathirae*	Bacteria	–	–	–	TYR	[199]
*Sm*NspF	*Streptomyces murayamaensis*	Bacteria	D6RTB9	–	–	TYR	[27]
*Sr*OSRT	*Streptomyces* sp. REN-21	Bacteria	–	–	–	TYR	[200]
*Sz*TYR	*Streptomyces* sp. ZL-24	Bacteria	A0A2S3Y8X7	–	–	TYR	[201]
*Vs*TYR	*Verrucomicrobium spinosum*	Bacteria	WP_009958178*	Yes	C	TYR	[202]
*Ab*PPO1	*Agaricus bisporus*	Fungi	OU703638*	–	C	TYR	[85]
*Ab*PPO2	*Agaricus bisporus*	Fungi	O42713	–	C	TYR	[159]
*Ab*PPO3	*Agaricus bisporus*	Fungi	C7FF04	–	C	TYR	[85]
*Ab*PPO4	*Agaricus bisporus*	Fungi	C7FF05	–	C	TYR	[82]
*Ab*PPO5	*Agaricus bisporus*	Fungi	OU703645*	–	C	TYR	[85]
*Ab*PPO6	*Agaricus bisporus*	Fungi	OU703647*	–	C	TYR	[85]
*Ao*CO4	*Aspergillus oryzae*	Fungi	Q2UNF9	Yes	–	TYR	[71]
*Ao*MelB	*Aspergillus oryzae*	Fungi	Q2UP46	–	C	TYR	[203]
*Ao*MelO	*Aspergillus oryzae*	Fungi	Q00234	–	C	TYR	[107]
*Ao*TYR	*Armillaria ostoyae*	Fungi	A0A8A5MZC0	–	C	TYR	[204]
*Le*TYR	*Lentinula edodes*	Fungi	Q96TI3	–	C	CO	[205]
*Mt*PPO7	*Thermothelomyces thermophilus*	Fungi	A0A1C9CXH9	Yes	–	TYR	[206]
*Pa*TYR1	*Polyporus arcularius*	Fungi	Q65Z70	–	C	TYR	[207]
*Pn*TYR	*Pholiota nameko*	Fungi	A7BHQ9	–	C	TYR	[208]
*Ps*TYR	*Pycnoporus sanguineus*	Fungi	Q2TL94	–	C	TYR	[209]
*Tr*TYR2	*Trichoderma reesei*	Fungi	A0ZXZ4	Yes	C	TYR	[93]
*Tt*PPO	*Thermothelomyces thermophilus*	Fungi	G2QLD3	Yes	–	TYR	[210]
*Ag*PPO8	*Anopheles gambiae*	Insecta	Q8MZM3	–	N	TYR	[70]
*Ap*PPO	*Antheraea pernyi*	Insecta	–	–	N	CO	[211]
*Dm*PPO1	*Drosophila melanogaster*	Insecta	–	–	N	CO	[212]
*Dm*PPO2	*Drosophila melanogaster*	Insecta	–	–	N	CO	[213]
*Dm*PPO3	*Drosophila melanogaster*	Insecta	–	–	N	CO	[213]
*Of*PPO1b	*Ostrinia furnacalis*	Insecta	A0A1Q1MKI0	–	N	CO	[214]
*Of*PPO2	*Ostrinia furnacalis*	Insecta	Q2I355	–	N	CO	[214]
*Of*PPO3	*Ostrinia furnacalis*	Insecta	A0A1Q1MKI6	–	N	CO	[214]
*Ac*PPO	*Annona cherimola*	Plantae	A0A168	Yes	C	CO	[215]
*Bs*PPO	*Sesamum indicum*	Plantae	–	Yes	C	TYR	[216]
*Cg*AUS1	*Coreopsis grandiflora*	Plantae	A0A075DN54	Yes	C	CO	[217]
*Cs*PPO	*Camellia sinensis*	Plantae	Q68NI4	Yes	C	CO	[218]
*Cs*PPO1	*Camellia sinensis*	Plantae	–	Yes	C	CO	[219]
*Cs*PPO2	*Camellia sinensis*	Plantae	–	Yes	C	CO	[219]
*Cs*PPO3	*Camellia sinensis*	Plantae	A0A6B7JGJ4	Yes	C	CO	[220]
*Cs*PPO4	*Camellia sinensis*	Plantae	A0A7D0M1V4	Yes	C	n.r.	[220]
*Cr*PPO2	*Ceratopteris rattanii*	Plantae	–	Yes	C	TYR	[221]
*Dk*PPO	*Diospyros kaki*	Plantae	–	Yes	C	n.r.	[222]
*Dl*PPO1	*Dimocarpus longan*	Plantae	A0A8J9T919	Yes	C	TYR	[223]
*Ej*PPO2	*Eriobotrya japonica*	Plantae	I7CML7	Yes	C	CO	[160]
*Gb*PPO2	*Ginkgo biloba*	Plantae	–	Yes	C	TYR	[221]
HjyPPO1	*Camellia sinensis*	Plantae	–	Yes	C	CO	[224]
HjyPPO3	*Camellia sinensis*	Plantae	–	Yes	C	CO	[224]
*Ib*CO2	*Ipomoea batatas*	Plantae	Q5ENY2	Yes	C	i.a.	[160]
*Jr*PPO1	*Juglans regia*	Plantae	C0LU17	Yes	C	TYR	[119]
*Jr*PPO2	*Juglans regia*	Plantae	A0A2I4DDQ0	Yes	C	TYR	[126]
*Lt*LH	*Larrea tridentata*	Plantae	Q6UIL3	Yes	C	TYR	[225]
*Md*PPO1	*Malus domestica*	Plantae	A0A238GSN3	Yes	C	TYR	[100]
*Md*PPO2	*Malus domestica*	Plantae	A0A238GSS3	Yes	C	TYR	[100]
*Md*PPO3	*Malus domestica*	Plantae	A0A238GT27	Yes	C	TYR	[100]
*Md*PPO7	*Malus domestica*	Plantae	–	Yes	C	i.a.	[226]
*Mn*PPO1	*Morus notabilis*	Plantae	W9S216	Yes	C	TYR	[227]
*Mn*PPO2	*Morus notabilis*	Plantae	W9RL63	Yes	C	TYR	[227]
*Mn*PPO3	*Morus notabilis*	Plantae	W9RNM7	Yes	C	TYR	[227]
*Mn*PPO4	*Morus notabilis*	Plantae	W9S222	Yes	C	TYR	[227]
*Mn*PPO5	*Morus notabilis*	Plantae	W9RDC2	Yes	C	TYR	[227]
*Mp*AS1	*Marchantia polymorpha*	Plantae	BBN01239*	Yes	C	TYR	[228]
*Mp*PPO2	*Marchantia polymorpha*	Plantae	A0A176WB99	Yes	C	TYR	[221]
*Mp*PPO3	*Mucuna pruriens*	Plantae	–	Yes	C	TYR	[229]
*Oe*PPO1	*Olea europaea*	Plantae	QXT58294*	Yes	C	CO	[230]
*Oe*PPO2	*Olea europaea*	Plantae	QXT58295*	Yes	C	CO	[230]
*Oe*PPO3	*Olea europaea*	Plantae	WDQ36035*	Yes	C	TYR	[231]
*Oe*PPO4	*Olea europaea*	Plantae	WDQ36036*	Yes	C	i.a.	[231]
*Ofr*PPO1	*Osmanthus fragrans*	Plantae	–	Yes	C	CO	[221]
*Ofr*PPO2	*Osmanthus fragrans*	Plantae	–	Yes	C	TYR	[221]
*Ofr*PPO3	*Osmanthus fragrans*	Plantae	–	Yes	C	CO	[221]
*Ofr*PPO4	*Osmanthus fragrans*	Plantae	–	Yes	C	CO	[221]
*Pa*PPO	*Prunus armeniaca*	Plantae	O81103	Yes	C	TYR	[232]
*Pp*PPO1	*Pyrus pashia*	Plantae	F6L6G6	Yes	C	i.a.	[160]
*Pp*PPO4	*Pyrus pashia*	Plantae	D6P743	Yes	C	i.a.	[160]
*Pt*PPO11	*Populus trichocarpa*	Plantae	F8V189	Yes	C	n.r.	[233]
*Pt*PPO13	*Populus trichocarpa*	Plantae	F8V190	Yes	C	n.r.	[233]
*Rc*PPO	*Rosa chinensis*	Plantae	XP_024157533*	Yes	C	TYR	[234]
*Sl*PPO1	*Solanum lycopersicum*	Plantae	A0A3P3ZL83	Yes	C	TYR	[115]
*Sl*PPO2	*Solanum lycopersicum*	Plantae	A0A3P3ZKL1	Yes	C	CO	[115]
*Sl*PPOF	*Solanum lycopersicum*	Plantae	Q08296	Yes	C	n.r.	[235]
*Sm*PPO1	*Solanum melongena*	Plantae	C7E3U6	Yes	C	i.a.	[236]
*St*PPO1	*Solanum tuberosum*	Plantae	–	Yes	C	CO	[237]
*To*PPO1	*Taraxacum officinale*	Plantae	B2BX66	Yes	C	TYR	[118]
*To*PPO2	*Taraxacum officinale*	Plantae	B3WFP2	Yes	C	TYR	[118]
*To*PPO3	*Taraxacum officinale*	Plantae	G4U402	Yes	C	TYR	[118]
*To*PPO4	*Taraxacum officinale*	Plantae	G4U403	Yes	C	n.r.	[118]
*To*PPO6	*Taraxacum officinale*	Plantae	I7HUF2	Yes	C	CO	[238]
*To*PPO7	*Taraxacum officinale*	Plantae	U3UB86	Yes	C	CO	[118]
*To*PPO9	*Taraxacum officinale*	Plantae	U3UB71	Yes	C	n.r.	[118]
*Tp*PPO1	*Trifolium pratense*	Plantae	Q84YI1	Yes	C	CO	[239]
*Tp*PPO2	*Trifolium pratense*	Plantae	Q84YI0	Yes	C	CO	[239]
*Tp*PPO3	*Trifolium pratense*	Plantae	Q84YH9	Yes	C	CO	[239]
*Vv*PPO1	*Vitis vinifera*	Plantae	A0A024FR46	Yes	C	TYR	[163]
*Vv*PPO2	*Vitis vinifera*	Plantae	A0A024FS61	Yes	C	TYR	[240]
*Vv*PPO3	*Vitis vinifera*	Plantae	A0A0S2SVY3	Yes	C	TYR	[122]
*Zm*PPO2	*Zea mays*	Plantae	–	Yes	C	TYR	[221]

For proteins that have been the target of multiple recombinant production attempts only the most successful (i.e., the one yielding the broadest enzymatic activity: TYR > CO > n.r. > i.a.) and/or earliest one is listed here. This list does not include expression studies that deal exclusively with mutated proteins. Entries are sorted alphabetically, first by group and then by the name of the PPO

^a^For prokaryotic PPOs “Group” provides the phylogenetic domain to which the PPO-producing organism belongs while for eukaryotic PPOs the kingdom or class (in the case of insect PPOs) is given

^b^Unique identifier for the protein in the UniProt database; *NCBI-accession (GenPept database)

^c^Indicates the presence (yes) or absence (–) of an N-terminal signal sequence in the PPO-encoding gene

^d^Gives the position of the lid-like, active site shielding domain; *N* N-terminal, *C* C-terminal, – no shielding domain

^e^Broadest reported activity of the expressed enzyme; *TYR* activity on at least one monophenol is reported,* CO* reported activity only on catechols, n.r. no report of enzymatic activity, i.a. inactive enzyme was produced

Reports stating the production yield of recombinant PPOs are rare, and roughly half of all reported yields are lower than 10 mg of purified enzyme per liter of culture of the respective expression host (Table 3). It is also of note that different expressions of the same enzyme do generally not yield enzyme preparations possessing equal enzymatic activity. In fact the reported specific activities of human tyrosinase (*Hs*TYR), for which the largest number of expression trials is reported, vary over three orders of magnitude from 17.3 U mg^−1^ on 1 mM l-tyrosine [241] to 4.8 U g^−1^ (i.e., ≈3600-fold lower) [242] and also include a number of unsuccessful experiments that yielded inactive protein.

**Table 3:** Expressions of PPOs, sorted by yield of purified enzyme per liter of culture medium

Enzyme	Expression host	PPO form^a^	Fusion partner^b^		*T*^c^/°C	Cu^d^/µM	*Y*^e^/mg l^−1^	*A*_spec_^f^/U mg^−1^	References
			N-terminal	C-terminal					
*Tr*TYR2	*Trichoderma reesei*	Latent	CBH1.S	–	30	2000	2160^#^	n.r.	[243]
*Vs*TYR	*Escherichia coli*	Active	His_6_	–	30	–	240	n.r.	[244]
*Md*PPO1	*Escherichia coli*	Latent	**GST**	–	20	500	225	0.24	[100]
*Vs*TYR	*Escherichia coli*	Latent	–	–	32	7.3	170^#^	n.r.	[245]
*Ab*PPO4	*Escherichia coli*	Latent	**GST**	–	20	500	140	1.2	[82]
*Bm*TYR	*Escherichia coli*	Active	–	His_6_	37	–	86	4.50	[104]
*Sl*PPO1	*Escherichia coli*	Latent	**GST**	–	25	500	80	n.r.	[115]
*Jr*PPO2	*Escherichia coli*	Latent	**GST**	–	20	500	70	0.73	[126]
*Md*PPO2	*Escherichia coli*	Latent	**GST**	–	20	500	60	0.018	[100]
*Pa*TYR1	*Escherichia coli*	Latent	His_6_	–	20	200	54	n.r.	[207]
*Sr*OSRT	*Escherichia coli*	Active^§^	–	–	25	1000	54	53	[200]
*Tr*TYR2	*Trichoderma reesei*	Latent^+^	His_6_	–	28	2000	45	0.84	[93]
*Pa*PPO	*Escherichia coli*	Latent	**GST**	–	18	500	45	n.r.	[232]
*Jr*PPO1	*Escherichia coli*	Latent	**GST**	–	20	500	41	2.6	[126]
*Tt*PPO	*Pichia pastoris*	Active	–	Myc-His_6_	23	25	40	n.r.	[210]
*Sg*MelC2	*Streptomyces glaucescens*	Active	–	–	30	20	40^$^	n.r.	[246]
*Nk*TYR	*Escherichia coli*	Active	–	–	20	–	30	0.54	[182]
*Cg*AUS1	*Escherichia coli*	Latent	**GST**	–	25	500	30	n.r.	[113]
*Vs*TYR	*Escherichia coli*	Active	–	His_6_	20	–	27.1	n.r.	[247]
*Md*PPO3	*Escherichia coli*	Latent	**GST**	–	20	500	26.5	0.064	[100]
*Sa*MelC2	*Pseudomonas fluorescens*	Active^§^	–	–	25	–	26	n.r.	[248]
*Tr*TYR2	*Pichia pastoris*	Latent^+^	His_6_	–	22	10	24	0.026	[249]
*Sz*TYR	*Escherichia coli*	Active	–	–	19	500	24	5.83	[201]
*Ao*MelO	*Escherichia coli*	Latent	**TrxA**-His_6_-S.tag	–	30	–	20	n.r.	[107]
*Vs*TYR	*Escherichia coli*	Latent	–	–	37	–	20	260	[202]
*Ao*MelB	*Escherichia coli*	Latent	HAT	–	15	1000	20	78	[132]
*Ps*TYR	*Aspergillus niger*	Latent	SSS	–	30	–	20	30	[209]
*Sg*MelC2	*Escherichia coli*	Active	–	–	25	1000	20^#^	n.r.	[250]
*Hs*TYR	*Homo sapiens*	Active	–	His_6_	37	–	15.6	n.r.	[173]
*Vs*TYR	*Escherichia coli*	Active	His_6_-T7.tag-Xpress.tag	–	n.r.	–	15.1	n.r.	[251]
*Sc*MelC2	*Escherichia coli*	Active^§^	–	His_6_	28	–	11	n.r.	[252]
*To*PPO2	*Escherichia coli*	Latent	Strep2.tag	–	26	200	8.3	n.r.	[235]
*Hs*TYR	*Spodoptera frugiperda*	Active	–	His_6_	21	2000	8	2.2	[253]
*Ab*PPO1	*Escherichia coli*	Latent	TEE-His_6_-***Sc*Smt3**	–	12	500	6	2.0	[85]
*Cg*AUS1	*Escherichia coli*	Latent	–	–	37	1000	6	n.r.	[217]
*Hs*TYR	*Trichoplusia ni*	Active	MSS	His_6_	n.r.	n.r.	6	n.r.	[174]
*Dl*PPO1	*Escherichia coli*	Latent	**GST**	–	17.5	2000	5.7	0.27	[223]
*Hs*TYR	*Escherichia coli*	Active	–	His_6_	37	1000	3.8	17	[241]
*Rc*PPO	*Escherichia coli*	Latent	**MBP**	His_6_	18	–	3.4	0.001	[234]
*Hs*TYR	*Escherichia coli*	Active	–	His_6_	n.r.	1000	2.8*	0.55	[254]
*Sa*MelC2	*Escherichia coli*	Active	–	–	37	–	2.5	n.r.	[248]
*To*PPO2	*Escherichia coli*	Latent	Strep2.tag	–	26	20	2.1	n.r.	[127]
*To*PPO6	*Escherichia coli*	Latent	Strep2.tag	–	26	20	2	n.r.	[238]
*Sl*PPO2	*Escherichia coli*	Latent	**GST**	–	20	500	2	n.r.	[115]
*Vv*PPO1	*Escherichia coli*	Active	His_6_	–	18	–	2	0.031	[163]
*Mp*PPO3	*Escherichia coli*	Latent	His_6_	–	37	–	2	5.2	[229]
*Ab*PPO4	*Escherichia coli*	Latent	TEE-His_6_-***Sc*Smt3**	–	12	500	1.8	1.2	[85]
*Hs*TYR	*Trichoplusia ni*	Active	ISS	His_6_	n.r.	n.r.	1.6^$^	0.010	[255]
*To*PPO6	*Escherichia coli*	Latent	Strep2.tag	–	26	20	1.2	n.r.	[127]
*Hs*TYR	*Escherichia coli*	Active	His_6_-T7.tag-Xpress.tag	–	30	–	1	n.r.	[172]
*Ab*PPO3	*Escherichia coli*	Latent	TEE-His_6_-***Sc*Smt3**	–	12	500	0.87	0.35	[85]
*Ao*CO4	*Trichoderma reesei*	Active^+^	–	–	n.r.	500	0.60	n.r.	[71]
*Ab*PPO2	*Escherichia coli*	Latent	TEE-His_6_-***Sc*Smt3**	–	12	500	0.50	51	[85]
*Ab*PPO2	*Saccharomyces cerevisiae*	Latent	–	V5.tag-His_6_	18	1000	0.46*	11	[159]
*Ab*PPO6	*Escherichia coli*	Latent	TEE-His_6_-***Sc*Smt3**	–	12	500	0.16	0.04	[85]
*Ab*PPO5	*Escherichia coli*	Latent	TEE-His_6_-***Sc*Smt3**	–	12	500	0.16	27	[85]
*Sg*GriF	*Escherichia coli*	Active^§^	–	His_6_	26.5	–	0.16*	n.r.	[26]

^a^Latent PPOs contain the central tyrosinase domain and the C-terminal lid-like domain that covers the type III copper site while active PPOs consist of only the central tyrosinase domain.^§^For these streptomycetal PPOs coexpression of the respective caddie protein was employed. ^+^These fungal PPOs were expressed with their respective native secretion sequences present and have been purified from the culture medium

^b^Proteins applied as fusion partners are marked with bold letters. The abbreviations used for units translationally fused to the respective PPO are *CBH1.S* signal peptide of cellobiohydrolase I from *Trichoderma reesei*, *His*_*6*_ hexahistidine tag for immobilized metal affinity chromatography, *GST* glutathione S-transferase from *Schistosoma japonicum* (25 kDa), *Myc* 10 amino acid epitope tag of the human c-Myc oncogene, *TrxA* *E. coli* thioredoxin 1 (12 kDa), *S.tag* 15 amino acid affinity and epitope tag derived from pancreatic ribonuclease A, *HAT* 19 amino acid histidine affinity tag containing six histidines, *SSS* 24 amino acid synthetic signal sequence derived from the *Aspergillus niger* glucoamylase gen, *T7.tag* 11 amino acid leader peptide of the bacteriophage T7 gene 10, *Xpress.tag* Xpress™ epitope tag with enterokinase recognition and cleavage site (DLYDDDDK), *Strep2.tag* Strep-Tactin® binding octapeptide (WSHPQFEK), *TEE* 5 amino acid translation enhancing element (MNHKV), *ScSmt3* *S. cerevisiae* ubiquitin-like protein (11 kDa), *MSS* 21 amino acid honey bee melittin signal sequence, *MBP* *E. coli* maltose binding protein (41 kDa), *ISS* insect signal sequence (undisclosed), *V5.tag* 14 amino acid epitope tag from simian virus 5

^c^Cultivation temperature during the induction step of expression; *n.r.* not reported

^d^Concentration of copper(II) added to the culture media as either CuSO_4_ or CuCl_2_; *n.r.* not reported

^e^Yield of purified enzyme (after removal of large fusion partners) per liter of culture medium employed;^#^yield before purification, *culture volume of 1 l was assumed,^$^mg protein purified from 10 g of mycelia or larval biomass

^f^Specific activity of the expressed and purified proteins; the presented values were estimated from the published kinetic data and are given for the reaction on 1 mM l-tyrosine. One unit of enzymatic activity is defined as the amount of enzyme that catalyzes the formation of 1 µmol product per min. *n.r.* not reported

Inspection of Table 3 hints at a yield-enhancing effect of larger, autonomously folding fusion partners, especially for the expression of eukaryotic PPOs. In the upper half of the table, one-third of expression trials that yielded more than 10 mg of protein per liter made use of a second protein translationally fused to the target PPO, with this ratio rising to almost one-half if only the top third of expression yields is considered. To avoid overinterpretation of these numbers it should be mentioned that all but two (*Ao*MelO, which was produced as a TrxA-fusion protein and *Rc*PPO, for which fusion to MBP was used) of the expression trials in Table 3 that rely on a second protein fused to the target PPO were reported by the same group (Rompel workgroup, University of Vienna: 17 of the 57 entries in this table) and use one of two solubility-enhancing fusion partners (GST or *Sc*Smt3).

If a protein is applied as a fusion partner to enhance heterologous expression, it is located N-terminal to the target PPO in most of the reported recombinant PPO production trials. None of the few studies using a C-terminal proteinaceous fusion partner reported a number for the yield of the respective target PPO. This may be due to the presence of signal peptides in many, especially eukaryotic, PPOs: these enzymes were probably not optimized to fold with the N-terminus of the central tyrosinase domain being free when exiting the ribosome. Yield enhancement by using larger fusion partners usually results mainly from an increase in solubility of the expressed fusion protein in contrast to the “passenger” protein expressed alone [256].

The most common fusion partners employed for solubility improvement in the *E. coli* expression host are GST (glutathione S-transferase from *S. japonicum*, 25 kDa) [257], TrxA (*E. coli* thioredoxin 1, 12 kDa) [258], MBP (*E. coli* maltose binding protein, 41 kDa) [259], and NusA (N-Utilization substance from *E. coli*, 55 kDa) [260]. The actual effect of adding a fusion partner to the expression construct depends heavily on the passenger protein’s properties and the employed expression system. Addition of a fusion partner may well be detrimental to the yield and/or solubility of the target protein and as no reliable prediction method exists yet the selection of a suitable fusion partner remains a trial-and-error process.

A second striking observation is that for most PPO expression trials the temperature during the induction step was chosen by considering the original host of the enzyme rather than the expression host’s favored growth conditions. Assuming that this parameter was optimized in most of the studies reported in Table 3, this may indicate folding of the respective target PPO as a major limiting step for the achievable protein production yield. Translation by bacterial ribosomes proceeds significantly faster (around 20 amino acids per second in fast growing *E. coli*) than what is expected for eukaryotic ribosomes (3–8 amino acids per second) and the beneficial effect of lower translation rates on the folding efficiency of eukaryotic proteins has been shown using mutant ribosomes in *E. coli* cells which allowed the production of aggregation prone eukaryotic proteins that could not be produced in bacterial systems before [261]. Lowering the cultivation temperature is expected to slow the bacterial metabolism, including the ribosomal translation of mRNA messages, and in doing so is likely to increase the ratio of correctly folded eukaryotic proteins. Of all production trials of PPOs that yielded 20 mg or more target protein per liter of culture (upper half of Table 3) only two—bacterial—enzymes (*Bm*TYR and *Vs*TYR) were produced at the optimal growth temperature of *E. coli* (37 °C) while for the high-yield production of eukaryotic PPOs temperatures between 15 and 30 °C were applied.

Co-expression of chaperones seems to have been less successful than reducing the temperature during the protein production; for only two production attempts the simultaneous overexpression of the *E. coli gro*ES-*gro*EL chaperonin system (via the plasmid pGro7 from the chaperone plasmid set marketed by Takara Bio Inc.) was reported, both are listed in Table 3: *Pa*TYR1 [207] and *Nk*TYR [182].

### Applications of TYRs

The unique reactivity of TYRs has found applications in organic and analytical chemistry, the biotechnological removal of pollutants from the environment as well as in food biotechnology and medicine. The majority of applications utilize “mushroom tyrosinase” (*Ab*T) isolated from fruiting bodies of *Agaricus bisporus* but especially for applications with a medical focus recombinant TYRs gained more prominence in the last few years.

TYRs are used both free (i.e., as freely dissolved proteins in a homogenous aqueous solution) and immobilized on a solid support. Immobilization of TYRs is often applied to increase the stability of the enzyme, e.g., to allow substrate conversion at high temperatures, extreme pH, or in the presence of elevated levels of organic solvents. The use of immobilized enzymes also allows easy recovery of the catalyst after the reaction and in combination with the improved enzyme stability may enable the reuse of the immobilized TYR for a number of additional reactions. Transition from homogenous to heterogenous catalysis generally comes with a penalty in reaction rate due to less effective mass transport to and from the active sites of the immobilized enzymes, but the improved stability usually overcompensates for this effect and/or the specific application requires immobilization.

#### Enzymatic synthesis of catechols

TYR activity provides a synthetic route to every catechol for which the corresponding monophenol is available. The major obstacle every catechol synthesis scheme employing TYRs has to overcome is the formation of *o*-quinones instead of catechols by these enzymes. The highly reactive *o*-quinones have to be converted to the target catechol, and this process requires a ready source of two electrons and two protons, i.e., a reductant. The reductant needs to reduce the *o*-quinones before they can polymerize and precipitate, inactivate the enzyme, or cause chemical damage to another component of the reaction system (e.g., fouling of membranes).

**Scheme 7:**
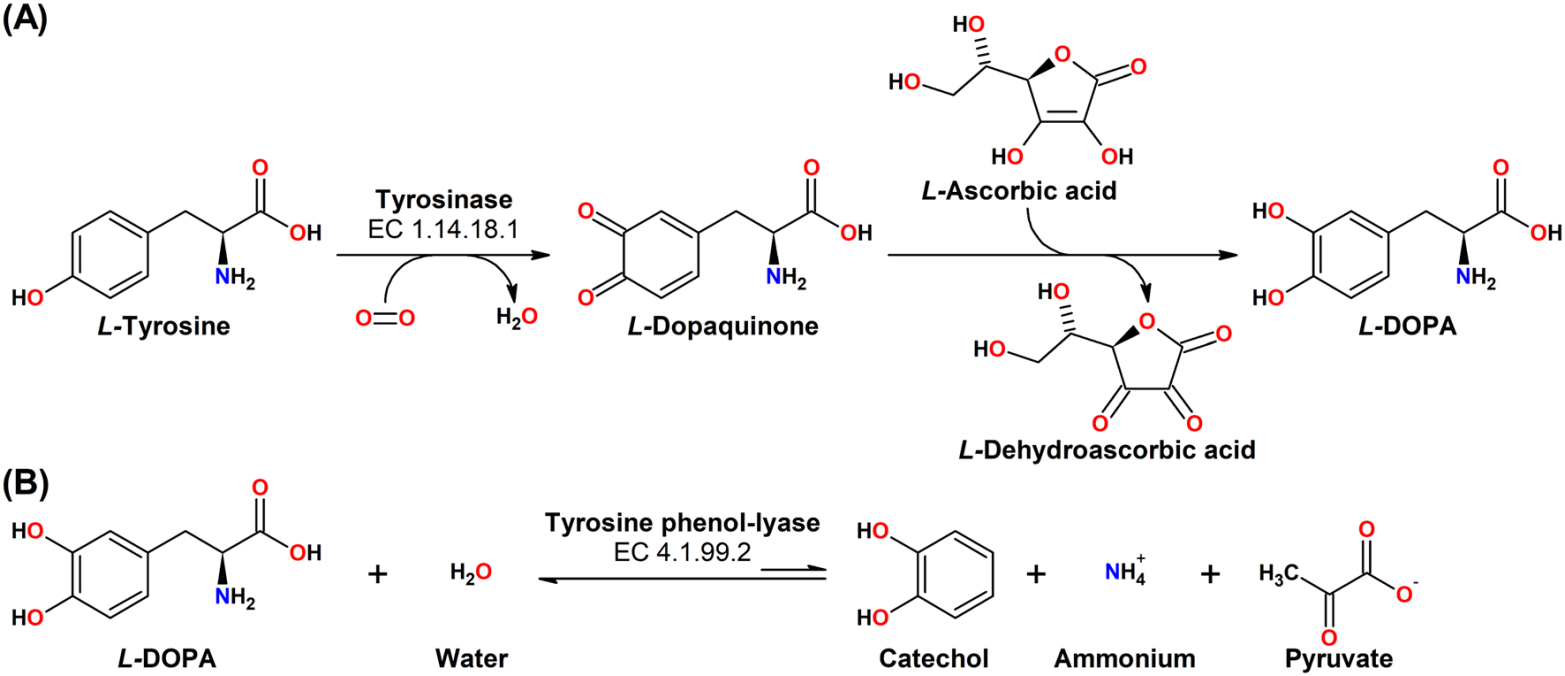
Biotechnological production of l-DOPA by TYR and tyrosine phenol-lyase. **A** *o*-Hydroxylation and oxidation of l-tyrosine to l-dopaquinone by TYR (EC 1.14.18.1) followed by reduction of the *o*-quinone to l-DOPA by ascorbic acid. **B** Formation of l-DOPA from catechol, ammonium, and pyruvate catalyzed by tyrosine-phenol lyase (EC 4.1.99.2) using the reverse reaction of the enzyme

The first enzymatic conversion of a phenol to the corresponding catechol was reported in 1969 by Satō [262] who achieved the production of 3 g l^−1^ h^−1^ of caffeic acid from *p*-coumaric acid using a crude preparation of TYR from *A. bisporus*. That report also recommended the use of ascorbic acid (AA) to reduce the enzymatically formed *o*-quinones to the target catechols, and ascorbic acid has remained the reductant of choice for almost all reported catechol production schemes using TYRs to this day (Scheme 7A).

The dopamine metabolite 3,4-dihydroxyphenylacetic acid has been produced by *Ab*T (TYR isolated from *A. bisporus* fruiting bodies) immobilized as cross-linked enzyme aggregates (341 mg l^−1^ h^−1^ @ 15 mM AA) [263], in intact fungal cells embedded in alginate beads (250 mg l^−1^ h^−1^ @ 25 mM AA) [264], immobilized on glass beads (160 mg l^−1^ h^−1^ @ 5.5 mM AA) [265], and immobilized on two epoxy-resins (Eupergit CM: 145 mg l^−1^ h^−1^ without reductant in the enzymatic reaction but with sodium dithionite workup afterwards [266] and EupergitC250L: 59 mg l^−1^ h^−1^ @ 15 mM AA [267]).

For the enzymatic synthesis of theaflavins from tea polyphenols no extra reductant is added, the catechins used as educts have sufficient antioxidant activity. Theaflavin-3,3ʹ-digallate was produced with the free recombinant bacterial enzyme *Bm*TYR from *Bacillus megaterium* (1920 mg l^−1^ h^−1^ [268] and 1473 mg l^−1^ h^−1^ [269]), the free recombinant plant enzymes *Rc*PPO2 from *Rosa chinensis* (491 mg l^−1^ h^−1^) [234] and *Cs*PPO1 from *Camellia sinensis* (204 mg l^−1^ h^−1^) [220], and the crude free plant enzyme *St*PPO isolated from potatoes (*Solanum tuberosum*, 72 mg l^−1^ h^−1^) [270].

No reductant is needed for the synthesis of bacterial melanin, the end product of the non-enzymatic evolution of TYR-generated *o*-quinones. Melanin was produced with living cells expressing recombinant *Bm*TYR (320 mg l^−1^ h^−1^ with *Vibrio natriegens* [170], 100 mg l^−1^ h^−1^ with *E. coli* [271]), *Pk*TYR from *Pseudomonas koreensis* cells (115 mg l^−1^ h^−1^) [272], recombinant *Re*MelA from *Rhizobium etli* (27 mg l^−1^ h^−1^ in *E. coli*) [273], and *Ds*TYR from *Dietzia schimae* cells (11 mg l^−1^ h^−1^) [274].

##### Production of l-DOPA

Production of l-DOPA, an important drug for treatment of patients afflicted with Parkinson’s disease [275], has received much attention and the route via TYR is especially promising because it requires only readily available l-tyrosine and molecular oxygen (Table 4). The production of the catechol l-DOPA by TYRs is, however, severely hampered by the low solubility of l-tyrosine (≈2 mM in water at neutral pH and 25 °C) in the usually applied aqueous buffers which limits both the reaction speed and the achievable product concentration and therefore puts an upper limit on the possible space–time yield. This limitation was overcome by using a dispersion of 500 mM l-tyrosine and efficient reduction of the generated *o*-quinones to the target catechol by a glassy carbon electrode and a space–time yield of 15.3 g l^−1^ h^−1^ of l-DOPA was attained [276]. This excellent yield was achieved with a crude TYR preparation from the mushroom *Agaricus bisporus* containing mainly TYRs with medium catalytic efficiency for the substrate l-tyrosine (*Ab*PPO3: *k*_cat_* K*_M_^−1^ for l-tyrosine = 0.37 s^−1^ mM^−1^, *Ab*PPO4: 1.6 s^−1^ mM^−1^) [85], so a substantial improvement should be possible with a TYR optimized for fast hydroxylation of l-tyrosine.

**Table 4:** Enzymatic production of 3,4-dihydroxyphenylalanine using TYRs

Enzyme^#^	Source^$^	Encapsulation of the enzyme	l-DOPA/(mg l^−1^ h^−1^)	Reductant^§^	References
*Ab*T	Commercial	Immobilized on a carbon nanotube /polypyrrole composite electrode	15,300	Electrochemical	[276]
*Yl*TYR	Yeast culture	Intact yeast cells	6960	28 mM AA	[277]
*Bm*TYR	Recombinant	Free	351	5 mM AA	[281]
*Ab*T	Mushrooms	Cross-linked enzyme aggregates	209	4 mM AA	[282]
*Vs*TYR	Recombinant	In polyhydroxyalkanoate nano-granules	149	5 mM AA	[283]
*Ao*MelX	Fungal cells	Living mycelium	145	28 mM AA	[284]
*Ab*T	Mushrooms	Free	143	2 mM AA	[285]
*Ab*T	Commercial	Immobilized on the zeolitic imidazolate ZIF-8 and encapsulated in alginate beads	140	12 mM AA	[286]
*Ab*T	Commercial	Immobilized in Cu-alginate	110	2.5 mM AA	[287]
*Mx*PPO	Banana peel	Encapsulated in agar–agar beads	63	2.5 mM AA	[288]
*Ab*T	Commercial	Adsorbed on chitosan flakes	54	2.5 mM AA	[289]
*Ab*T	Commercial	Adsorbed on zeolithes	36	2.5 mM AA	[290]
*Ab*T	Commercial	Immobilized on nylon 6.6 membranes	33	2.5 mM AA	[291]
*Ao*MelX	Fungal cells	Living mycelium	28	100 mM glucose	[292]
*Ab*T	Commercial	Immobilized on polyamide membranes	23	2.5 mM AA	[293]
*Rs*TYR	Bacterial cells	Free (secreted from living cells)	8.3	4 mM AA	[294]
*Ab*T	Commercial	Adsorbed on polymethylchloride styrene	5.5	2.5 mM AA	[295]

^#^*Ab*T “mushroom tyrosinase” isolated from *Agaricus bisporus* (predominantly *Ab*PPO3 and *Ab*PPO4), *Yl*TYR TYR from the yeast *Yarrowia lipolytica*, *Bm*TYR bacterial TYR from *Bacillus megaterium*, *Vs*TYR bacterial TYR from *Verrucomicrobium spinosum*, *Ao*MelX fungal TYRs from *Aspergillus oryzae*, *Mx*PPO TYR isolated from an unspecified banana species (*Musa* sp.), *Rs*TYR bacterial TYR from *Ralstonia solanacearum*

^$^All commercial TYR preparations in this table were purchased from Sigma-Aldrich (now a subsidiary of Merck KGaA): “Tyrosinase from mushroom”, catalogue number T3824

^§^*AA* l-ascorbic acid; AA is usually applied in the form of its sodium salt to avoid acidification of the reaction medium

Efficient conversion of l-tyrosine to l-DOPA in homogenous solution was also achieved with pre-grown *Yarrowia lipolytica* NRRL-143 yeast cells that could synthesize 17.6 mM l-DOPA from 19.3 mM l-tyrosine (at pH 3.5, in the presence of 28 mM ascorbic acid) provided that 2 g l^−1^ diatomite (kieselgur) was added 15 min after the start of the reaction (total reaction time 30 min) [277].

For l-DOPA a competing enzymatic production route exists that uses the reverse reaction of tyrosine phenol-lyase (EC 4.1.99.2) starting from catechol and pyruvate (Scheme 7B). This route does not require the addition of a reductant as tyrosine phenol-lyase does not oxidize catechols. Current industrial production is based on tyrosine-induced (to trigger expression of the tyrosine phenol-lyase) wild-type *Pantoea agglomerans* (old name *Erwinia herbicola*) bacteria and yields 375 mg l^−1^ h^−1^ l-DOPA [278]. Significantly higher space–time yields can be achieved by applying the enzyme from the bacterium *Fusobacterium nucleatum* which is an efficient tyrosine phenol-lyase and allows the production of 5.2 g l^−1^ h^−1^ l-DOPA by *E. coli* cells expressing the recombinant enzyme [279]. Even higher productivity was demonstrated for *P. agglomerans* bacteria genetically modified for constitutive expression of tyrosine phenol-lyase that allowed the production of up to 11.1 g l^−1^ h^−1^ of l-DOPA in tyrosine-free media [280].

#### TYR-based biosensors

The *o*-hydroxylation and oxidation of phenolic molecules catalyzed by TYRs moves two electrons to each processed phenol (Scheme 5). To make a biosensor, TYR is immobilized on the surface of an electrode which allows the measurement of the enzymatically generated currents (amperometry) and finally the determination of the analyte concentration in question.

The first entry in Table 5 uses a solution of the pre-substrate phenyl phosphate from which the enzymatic action of alkaline phosphatase liberates phenol that is in turn hydroxylated and oxidized by the TYR immobilized on the surface of a graphite-Teflon composite electrode [296]. The combination of two enzymatic activities results in an excellent sensitivity with a detection limit of 40 molecules of alkaline phosphatase per nanoliter (1 nl = 0.1 mm × 0.1 mm × 0.1 mm).

**Table 5:** Biosensors based on TYR immobilized on the electrode

Enzyme^#^	Source^$^	Immobilization of the enzyme	Analyte	LoD^§^	References
*Ab*T	Commercial	In a graphite-Teflon composite	Alkaline phosphatase	67 fM	[296]
*Ab*T	Commercial	TYR-labeled anti-parathyroid hormone immunoglobulin G (sandwich assay)	Parathyroid hormone	0.2 pM	[297]
*Ab*T	Commercial	On a gold-coated cantilever	17β-Estradiol Estrone	0.36 pM 1.5 pM	[298]
*Ab*T	Commercial^a^	On porous carbon	Prostate-specific antigen	0.4 pM	[299]
*Ab*T	Commercial	On a gold-coated cantilever	17β-estradiol Estrone	1.6 pM 1.9 pM	[300]
*Ab*T	Commercial	In a chitosan film	Phenol	50 pM	[301]
*Ab*T	Mushrooms	In a in polyacrylamide gel	Free copper ions	0.16 nM	[302]
*Ab*T	Commercial	On the gate of a field-effect transistor	Dopamine	3.3 nM	[303]
*Ab*T	Commercial	On magnetic carbon nanotubes	Phenol	5 nM	[304]
*San*MelC2	Recombinant	In polyvinyl alcohol, coupled to a Clark electrode	l-DOPA Adrenaline	5 nM 5 nM	[305]
*Ab*T	Commercial	Encapsulated in and cross-linked with polyacrylamide	Bisphenol A	12 nM	[306]
*Ab*T	Commercial	On Fe_3_O_4_-chitosan nanoparticles	Catechol	25 nM	[307]
*Ab*T	Commercial	On a carbon nanotube–cobalt phthalocyanine–silk fibroin composite	Bisphenol A	30 nM	[308]
*Ab*T	Commercial^b^	In a polyacrylonitrile film containing aniline	Catechol Phenol *p*-Cresol *m*-Cresol	50 nM 100 nM 200 nM 200 nM	[309]
*Ab*T	Commercial	On TiO_2_ nanotubes grown on graphene	Catechol	55 nM	[310]
*Ab*T	Commercial	Enzyme mixed in a graphite layer	Phenol	200 nM	[311]
*Ab*T	Commercial	Cross-linked with BSA	Leupeptin	250 nM	[312]
*Ab*T	Commercial	On copper oxide nanoparticles	Dopamine	300 nM	[313]
*St*PPO	Potato peel	Crude enzyme preparation dried on filter paper together with 3 mM 3-methyl-2-benzothiazolone hydrazone	Catechol 4-Methylcatechol Phenol *p*-Cresol	500 nM 500 nM 500 nM 500 nM	[314]
*Ct*TYR	Yeast cells	Yeast cells in a carbon paste	Adrenaline Dopamine	2.3 µM 8 µM	[315]
*Ab*T	Commercial	On polypyrrole-functionalized single wall carbon nanotubes	Dopamine	5 µM	[316]
*Ab*T	Commercial	In a polythiophene/polypyrrole matrix	Tea polyphenols	85 µM	[317]
*Ab*T	Commercial	On a polyvinyl chloride membrane	Catechol	365 µM	[318]

^#^*Ab*T “mushroom tyrosinase” isolated from *Agaricus bisporus* (predominantly *Ab*PPO3 and *Ab*PPO4), *San*MelC2 bacterial TYR from *Streptomyces antibioticus*, *St*PPO plant TYR isolated from potatoes (*Solanum tuberosum*), *Ct*TYR fungal TYR from the yeast *Candida tropicalis*

^$^The commercial TYR preparations referred to in this table were purchased from Sigma-Aldrich (now a subsidiary of Merck KGaA): “Tyrosinase from mushroom”, catalogue number T3824. ^a^From Sangon Biotechnology Co. ^b^From Amresco Inc

^§^*LoD* limit of detection, smallest analyte concentration that can be distinguished from the background signal

Immobilization of TYR on an indium-tin oxide electrode, selectively only in the presence of parathyroid hormone, was attained by coupling the TYR to an antibody specific for the analyte and coating the electrode surface with another analyte-specific antibody [297]. Parathyroid hormone present in the assayed solution binds to both antibodies and links them together, effectively anchoring TYR on the electrode surface. This sandwich-type assay strategy allowed the detection of as little as 130 parathyroid hormone chains per nanoliter.

##### Analytical applications of free TYRs

Besides the utilization of TYR-coated electrodes as biosensors, freely dissolved TYRs are also applied analytically. Measuring the length of the lag phase for the oxidation of 4-*tert*-butylcatechol by free *Ab*T (mushroom TYR from *Agaricus bisporus*) allows the determination of thiols in the solution (LoD for cysteine 2.12 µM, LoD for* N*-acetylcysteine 4.94 µM) [319]. Free *Ab*T has also been applied as part of a developing solution for thin-layer chromatography for detection of TYR inhibitors in natural products (LoD for kojic acid 1 ng ≈7 pmol per spot) [320]. Sensitive detection of dopamine (LoD 1.93 nM) was achieved using free *Ab*T by exploiting the quenching effect of the enzymatically generated *o*-quinones on the fluorescence of ROX (5(6)-carboxy-X-rhodamine) coupled to CdZnTeS quantum dots [321]. Inclusion of the genes for the bacterial TYR *San*MelC2 and its caddy protein *San*MelC1 in a conjugal plasmid allows for easy visual detection of the transfer of genetic material between mating bacterial strains in *mel*^−^ strains. Simple cultivation on solid medium containing tyrosine leads to easily discernible melanin formation only in those bacteria that received the plasmid and thus secrete the TYR [322]. Highly sensitive detection of the dithiocarbamate fungicide ziram (LoD 1.5 nM) was achieved by the *Ab*T-catalyzed oxidation of the catechol dye pyrocatechol violet coupled to boron-doped carbon nanodots, which quenches the fluorescence of the nanodots. Ziram in the analyte solution inhibits the TYR and thereby reduces the fluorescence quenching, resulting in a higher intensity of the nanodot fluorescence [323]. A detection system for the protease caspase-3 in human serum was designed that applies the latent form (with the lid-like C-terminal domain in place) of the bacterial enzyme *Vs*TYR from *Verrucomicrobium spinosum*: the protease removes the C-terminal domain, thereby activating the TYR, and the resulting enzymatic activity on l-DOPA added later to the assay solution can be used to determine the amount of protease in the original sample (LoD in human serum 3.5 U ml^−1^) [247].

#### Bioremediation

In addition to synthetic and analytic applications, TYRs are also applied for bioremediation, the biotechnological removal of contaminants from bodies of air, water, or soil. The majority of TYR applications in this field target phenolic contaminants in wastewater. While free *Ab*T has been applied for phenol removal in combination with chitosan for trapping the generated *o-*quinones [324, 325] or in combination with cyanide hydratase [326], most TYRs used in bioremediation are immobilized on various carriers. *Ab*T was used immobilized on aluminosilicates [327], in a cross-linked chitosan matrix [328], on aminopropyl-derivatized glass [329], coagulated with potash, ammonium, and iron alums [330], immobilized on silica aerogels [331], immobilized on cross-linked chitosan-clay beads [332], as cross-linked enzyme aggregates [333], on chitosan-gold nanoparticles/montmorillonite [334], in the pores of mesoporous silica nanoparticles [335], or on granular activated carbon or activated chitosan beads [336]. In the last few years, the use of magnetic supports has emerged as a trend in enzyme immobilization as it allows for a convenient separation of the catalyst from the treated medium. *Ab*T has been applied immobilized on magnetic iron oxide nanoparticles [337] and on β-cyclodextrin–azobenzene polymers coating metallic iron oxide particles [338].

Aside from the removal of phenolics in general from wastewater streams, the xenoestrogen bisphenol A found in polycarbonates and as a stabilizer in high-performance plastics is of special concern, especially in drinking water. For the removal of bisphenol A free *Ab*T [339, 340] or *AbT* encapsulated layer-by-layer chitosan [341], immobilized on polyacrylonitrile beads [342], as cross-linked enzyme aggregates [333], in whole fungal cells immobilized in alginate modified with colloidal silica [343], or in Cu_3_(PO_4_)_2_ nanoflowers/copper-alginate or Fe_3_O_4_-based metal–organic frameworks [344] was applied.

While much less numerous, applications of other TYRs beside *Ab*T for bioremediation have been reported: living cells of the plant-root associated fungus *Amylomyces rouxii* were effective for co-metabolic degradation of the recalcitrant pesticide pentachlorophenol when tyrosine was added to the culture medium [345]. Use of lignin building blocks pre-activated by the fungal TYR *Mt*PPO7 from *Myceliophthora thermophila* achieved a 75-fold improvement in the lytic polysaccharide monooxygenase (LPMO)-driven degradation of cellulose [346]. *Cs*PPO isolated from tea leaves (*Camellia sinensis*) and immobilized in magnetic iron oxide core–shell nanoparticles [347] and TYR isolated from apples and immobilized on a polypyrrole membrane [348] were applied for the removal of phenol from wastewater. Fungal TYR secreted from cells of *Pleurotus florida* was successfully applied in the biodegradation of gas oil up to a concentration of 10%(v/v) [349]. Recombinant bacterial *Bm*TYR from *Bacillus megaterium* immobilized on polyhydroxyalkanoate beads allowed the removal of bisphenol A and its analogues from water [350], and a crude preparation of *St*PPO isolated from potato peels (*Solanum tuberosum*) could remove the dichlorotriazine dye Reactive Blue 4 from wastewater [351].

#### Cross-linking of proteins and peptides

**Scheme 8:**
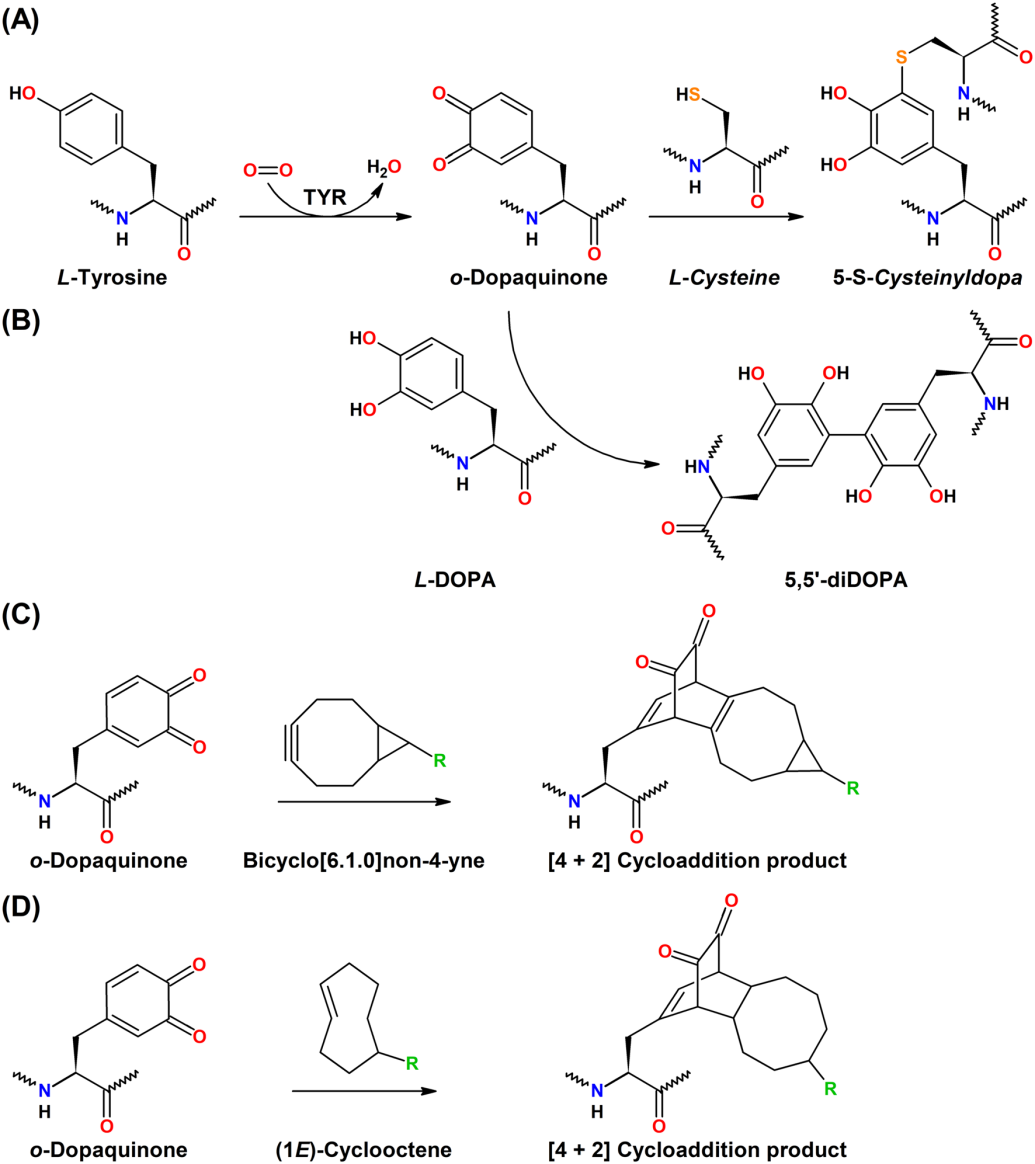
Cross-linking and bioorthogonal labelling of proteins using TYRs. **A** *o*-Hydroxylation and oxidation of l-tyrosine in a protein chain (indicated by the wiggly lines in place of the peptide bond) to l-dopaquinone by TYR (EC 1.14.18.1) and subsequent formation of a protein–protein cross-link with a cysteinyl residue from another protein; thiols form predominantely the depicted 5-S adduct while amines are more prone to form 6-S adducts with *o*-quinones [354]. **B** Protein cross-linking via diDOPA as observed in mussel foot proteins (which contain many l-DOPA residues) [355]. **C** Bioorthogonal labeling of proteins at surface tyrosyl residues with TYR-generated *o*-quinones and the strained bicyclo[6.1.0]non-4-yne that react in a hetero Diels–Alder reaction [356], the speed of which is greatly enhanced by the strain in the cyclooctyne ring. **D** A second example for bioorthogonal labelling of proteins using a cycloaddition that also employs a strained dienophile, in this case *trans*-cyclooctene [357], **R** indicates the label that is to be attached to the protein, e.g., a drug or a fluorescent molecule

Cross-linking of proteins by TYR is mainly caused by the Michael-type 1,4 or 1,6 addition of nucleophiles (most often thiol groups from cysteines in the protein) to the *o-*quinones generated from tyrosines by the enzymatic action of TYRs [352, 353] (Scheme 8). For proteins with a sufficient number of sterically available tyrosine side chains, cross-linking with TYR alone is usually sufficient; if not enough *o*-quinones can be generated from the protein itself, the addition of small phenolic or catecholic molecules like phenol or caffeic acid can help to achieve sufficient degrees of cross-linking. Since steric hindrance is a major limiting factor for protein cross-linking, the used TYRs are almost always applied in freely dissolved form.

Direct cross-linking with free *Ab*T has been reported for flour proteins [358], gluten [353], α-lactalbumin [359], gelatine and the polysaccharide chitosan [360], 3–10 kDa peptides from enzymatically hydrolyzed wool fibers [361], sericin peptides purified from silk textile industry wastewater [362], collagen I [363], silk fibroin [364], fibroin and elastin [365], shrimp tropomyosin to produce hypoallergens [366, 367], the peanut allergene Ara h 2 in order to reduce its allergenicity [368], potato proteins [369], and heat-denatured ovalbumin for the development of hypoallergenic food [370].

Cross-linking of proteins with free *Ab*T in the presence of small catechols is reported for sodium caseinate with catechol or chlorogenic acid [371] and whey proteins with caffeic acid [372] while for the gelation of chitosan with caffeic acid in mixtures of water and deep eutectic solvents, cross-linked enzyme aggregates mixed with magnetic iron oxide nanoparticles were applied [373].

Pigment formation limited to the inside of liquid droplets was achieved with *Ab*T freely dissolved in the medium and the amphiphilic peptide Asp-Tyr-Phe-Arg_9_ in the presence of adenosine triphosphate (ATP, to stimulate liquid–liquid phase separation and the formation of coacervates) [374]. Components were dissolved in aqueous buffers of low ionic strength and upon mixing the predominantly cationic peptide and the anionic adenosine triphosphate liquid–liquid phase separation with coacervation occurred. The coacervation process orients the amphiphilic peptides so that the tyrosyl residues face the inside of the formed droplets. To this mixture *Ab*T was added and formation of colored pigments was limited to the inside of the coacervates.

Free *Ab*T was applied for the cross-linking of a decellularized liver extracellular matrix hydrogel for the fabrication of a robust multiscale three-dimensional liver organoid [375] and the stiffening of gelatine-norbornene-hydroxyphenylacetic acid hydrogels cross-linked with 4-arm poly(ethylene glycol)-thiol for the culture of pancreatic cancer spheroids [376]. Cross-linking of cell-laden poly(ethylene glycol)-hydrogels with free *Ab*T and the peptide cross-linker Cys-Tyr-Gly-Gly-Gly-Tyr-Cys effected the stiffening of the hydrogel and allowed the cultivation of pancreatic stellate cells [377]. Similarly, but without the addition of peptides, the bacterial TYR *San*MelC2 from *Streptomyces antibioticus* was applied for the cross-linking of a protein-based hydrogel for stem cell cultivation [378].

Fungal *Ps*TYR isolated from fruiting bodies of *Pycnoporus sanguineus* was able to cross-link casein without the need for the addition of small catechols [379]. Treatment with *Tr*TYR2 isolated from the culture supernatant of the fungus *Trichoderma reesei* increased the firmness and water-holding capacity of low-meat chicken breast meat homogenate gels by forming a network of cross-linked collagen [380] and could cross-link β-casein [381]. Recombinant *Tr*TYR2 catalyzed the cross-linking of soy glycinin for the stabilization of oil-in-water emulsions [243].

The recombinant bacterial *Vs*TYR from *Verrucomicrobium spinosum* was effective in cross-linking bovine caseins without additives, and with the addition of 1 mM phenol or 0.55 mM caffeic acid also horse myoglobin, horse heart cytochrome *c*, hen egg white lysozyme, and *Candida antarctica* lipase B were cross-linkable [382].

Squid ink TYR from *Loligo formosana* could cross-link proteins in Sardine surimi gels, resulting in a more compact gel structure and increased strength of the gels [383]. Recombinant bacterial *Bm*TYR from *Bacillus megaterium* effected the oligomerization of four soy glycinin-derived tyrosine-containing peptides [384] and allowed the formation of a stable, gel-like emulsion by the enzymatic cross-linking of potato proteins in the aqueous phase and corn zein proteins in the oil phase [385]. Archaeal *Nk*TYR from *Nitrosopumilus koreensis* was produced in *Escherichia coli* and could trigger the gelation of stable chitosan (0.3% w/v)–gelatin (3% w/v) blends [386]. The plant TYR isolated from the corms of the elephant foot yam (*Amorphophallus campanulatus*) was applied to synthesize melanin from l-DOPA as an effective absorbent for the extraction of uranium from dilute solutions [387].

*Ab*PPO4 from the mushroom *Agaricus bisporus* efficiently catalyzed the hydroxylation and oxidation of tyrosine side chains in a peptide derived from a mussel-foot protein, which triggered the polymerization of the peptide into a water-resistant bioglue [388]. The glue was further improved by introduction of a switchable cohesion control module into the peptide that allows more efficient cross-linking by the TYR without sacrificing cohesion of the finished bioglue [389]. A living variant of a TYR-based bioglue was engineered by producing the bacterial TYR *San*MelC2 from *Streptomyces antibioticus* in *Bacillus subtilis* which converted the *B. subtilis* biofilms to cellular glues [390]. The bacterial *Bc*TYR from *Burkholderia cepacia* was applied for the synthesis of homogenous and soluble (< 20 nm diameter) melanin nanoparticles from dopamine at pH 3 [187].

##### Functionalization of proteins and surfaces

While cross-linking usually generates a three-dimensional network of covalent bonds, the same chemistry is also applicable for the joining of two entities or the modification of a surface. As for cross-linking reactions almost exclusively free TYRs are used for coupling and grafting reactions.

Free *Ab*T was applied for the grafting of peptides (Tyr-Ala and casein hydrolysate) onto the amine-containing polysaccharide chitosan [391], grafting of silk fibroin on chitosan [392], coupling of phenolic antioxidants (caffeic acid and chlorogenic acid) onto wool fiber proteins [393], conjugation of human serum albumin with an antibody using caffeic acid and* p*-coumaric acid as cross-linking mediators [394], screening of enzymatically activatable peptides for adhesion on Al_2_O_3_ [395], covalent immobilization of catalase onto silk fibroin [396], coimmobilization of the anticoagulant heparin and antibiotic silver nanoparticles for antithrombotic and antimicrobial applications [397], formation of uniform films of catechols on inorganic surfaces or living cells [398], modification of tyrosine in proteins and peptides (e.g., peptide antitumor agents, hormones, cyclic peptide antibiotics) [399], modification of proline residues in the N-terminal positions of proteins [400], site-specific labelling of proteins, e.g., for fluorophore labelling or the generation of antibody–drug conjugates [356], enzymatic immobilization of functional, tyrosine-containing peptides on gold nanoparticles [401], site-specific coupling of proteins using native amino acids leading to a 20-fold increase in genome editing efficiency of CRISPR-Cas9 by the conjugation of cell-penetrating peptides to Cas9 [402], conversion of monophenols into catechols for the formation of functional coatings [403], deposition of pseudo-polydopamine films for surface functionalization and coating [404], introduction of DOPA into silk sericin for coating of Ti surfaces in orthodontic and orthopedic applications [405], click chemistry labelling of proteins by oxidation of genetically encoded DOPA residues [406], substrate-independent surface coating and clickable film functionalization [407], chemical editing of Gram-positive bacterial cell walls (via teichoic acids embedded in the cell walls) [408], conjugation of nanobodies to cell surfaces via a tyrosine tag on the target protein [409], grafting of phlorizin (a chalcone) on soy proteins for functional foods [410], oxidation of protein-bound tyrosine for site-selective radiolabeling of antibodies with ^89^Zr chelated by deferoxamine for positron emission tomography [357], and conjugation of antibodies to drugs for targeted immunotherapy [411].

*Ab*T immobilized on an epoxy-functionalized resin (Immobead 150P) was applied for the selective bioconjugation leading to the formation of chimeric macromolecules like drug-loaded antibodies [412].

An enzymatic cascade using two TYRs allowing the selective oxidative coupling of proteins was engineered. First, *Ab*T hydroxylates and oxidizes an exposed tyrosine residue on the first protein but cannot access the target tyrosine on the second protein. The coupling between the two proteins is then done by a Michael-type addition of an engineered cysteine on the second protein with the activated tyrosine of the first protein. If needed, a second specific coupling on the same protein can be made using the recombinant bacterial *Bm*TYR from *Bacillus megaterium* which is less sterically hindered than *Ab*T and can access a second tyrosine on the first protein that is inaccessible to *Ab*T [413]. Use of either of the two TYRs *Ab*T or *Bm*TYR allows the conjugation of cysteine-substituted antibodies with drugs [414].

*Bm*TYR has also been used for the oxidative coupling of tyrosine-containing tags onto proteins and peptides [415], the site-specific protein modification by oxidation of C-terminal tyrosine residues (with the possibility of recovering the catalyst as *Bm*TYR is immobilized on magnetic beads) [188], and peptide cyclization to set up an mRNA display for discovery of macrocyclic peptide ligands against challenging protein targets [416]. Plant TYR isolated from the corms of *Amorphophallus campanulatus* was applied for the melanin-coating of the surface of a porous agarose-alginate-silica matrix usable for uranium recovery from dilute solutions [417]. The bacterial TYR *San*MelC2 was effective in the oxidation of tyrosyl residues in a mussel foot protein-derived peptide for the biocompatible functionalization of surfaces [418].

Recombinant *Ab*PPO4 from the mushroom *Agaricus bisporus* (and also *Ab*T) removed the 71 N-terminal amino acids from a bacterial carboxylesterase (*Cb*EstA from *Clostridium botulinum*), leading to exposure of the active site and a 32-fold increase in its hydrolytic activity on the polyester polyethylene terephthalate (PET) [90]. Note that the here applied TYR showed proteolytic activity, but inactived TYR (activity on phenols or catechols lost by treatment with an irreversible inhibitor or as the result of mutated amino acids at the active site) was also unable to cleave the bacterial esterase.

Co-expression of the bacterial *Vs*TYR from *Verrucomicrobium spinosum* with mussel foot protein introduced DOPA into the recombinant mussel foot protein, which increased its adhesivity [419] and also the in vitro hydroxylation of peptide-bound tyrosines in the mussel foot protein with *Vs*TYR worked and made the protein an adhesive [244]. Use of the bacterial TYR *Sa*MelC2 from *Streptomyces avermitilis* allows proteins to be modified site-specifically [420]; the PEGylation of sfGFP (superfolder green fluorescent protein) and the conjugation of a fluorescent dye to sfGFP by *Sa*MelC2 were demonstrated.

#### Medical applications

*Ab*T is rarely applied in patients but its use either immobilized in a metal–organic framework for the activation of the non-toxic prodrug paracetamol for cancer therapy [421], loaded into microreactors encapsulated in liposomes for the local depletion of tyrosine leading to the inhibition of melanoma growth [422], and free for the inhibition of tumor growth (measured in cell culture) [423] has been reported.

The melanized outer membrane vesicles synthesized by the *Escherichia coli* strain W3110-K12 expressing the recombinant bacterial TYR *Re*MelA from *Rhizobium etli* have been designed as biological nanoheaters for optoacoustic imaging and photothermal therapy [424].

Use of the human *Hs*TYR (*Homo sapiens*) overexpressed in melanoma cells has been suggested for the melanoma-specific activation of paracetamol to produce cytotoxic benzoquinone metabolites [425], for polymerization of therapeutic aspartic acid-phenylalanine-tyrosine tripeptides and cross-linking of proteins in melanoma cells for suppression of tumor growth and postsurgery recurrence [426], and the activation of a cyclometalated Pt(II) complex for melanoma photodynamic therapy and fluorescence imaging [427].

Recombinant bacterial TYR *Sa*MelC2 from *Streptomyces avermitilis* was applied encapsulated in liposomes [428] or on single-walled carbon nanotubes [429] delivered via microneedles into skin tissue for in vivo biosynthesis of artificial melanin for improved photoprotection against UV irradiation. *Sa*MelC2 was also successfully applied for the cross-linking of a modified chitosan hydrogel (glycol chitosan and hyaluronic acid modified with 4-hydroxyphenylacetic acid) containing pancreatic β-cell spheroids for long-term blood glucose regulation [430].

The bacterial *Bm*TYR from *Bacillus megaterium* expressed in the *Escherichia coli* strain Nissle 1917 was applied for the in situ synthesis of melanin in tumor tissue allowing improved photothermal immunotherapy [431]. To alleviate concerns against introducing live genetically modified bacteria into patients, the approach was later refined to apply not the living bacterial cells themselves but only the melanized outer membrane vesicles produced by the *Bm*TYR-expressing bacteria that were in addition covered with a thick layer of calcium phosphate [432].

The same approach was applied with the bacterial TYR *San*MelC2 from *Streptomyces antibioticus*, which was produced by recombinant *E. coli* cells for the in vivo, tumor-specific melanin production that allowed targeted photothermal therapy [168].

Crude *St*PPO isolated from potato peels (*Solanum tuberosum*) that was immobilized in magnetic cross-linked enzyme aggregates has proven effective for the inhibition of biofilm growth (*Escherichia coli*, *Staphylococcus aureus*, *Klebsiella pneumoniae*, methicillin-resistant *Staphylococcus aureus*, and *Candida albicans*) [433].

##### Hydrogels for improved wound healing

TYRs allow the stiffening of phenol-containing (e.g., from tyrosine) hydrogels without the addition of toxic chemical cross-linkers like acyl azides, Cr^3+^, formaldehyde, or glutardialdehyde, which markedly improves the safety profile of the final gels. As for other cross-linking applications, the used TYR is usually applied freely dissolved.

Cross-linking of a gelatin hydrogel with both *Ab*T and a peroxidase yielded a strong and adhesive gelatin hydrogel for tissue-regeneration applications [434].

Cross-linking with free *Ab*T resulted in the formation of tissue-adhesive hydrogels from tyramine-conjugated hyaluronic acid and gelatin for repair of meniscus (joint cartilage) tissue [435], allowed the fabrication of a bioink made of gelatin methacrylamide and collagen doped with TYR for the three-dimensional bioprinting of living skin tissues [436], induced dynamic matrix stiffening in a gelatin-hyaluronic acid hybrid hydrogel for the study of cell–matrix interactions in pancreatic ductal adenocarcinoma [437], effected the gelation of hydrogels made from star-shaped oligo(ethylene glycol)s modified with desaminotyrosine for controlled release of active pharmaceutical ingredients [438], modified soybean protein for the development of medical adhesives [439], triggered the gelation of silk fibroin hydrogels with enhanced adhesion to intestinal tissue for wound closure applications [440], initiated the gelation of a tissue-adhesive alginate hydrogel seeded with chondrocytes for cartilage repair [441], stiffened collagen encapsulating living cells for the manufacture of combined hard-soft tissue without a barrier in between them [442], gelled tyramine-alginate gels [443] and chitosan/silk fibroin hydrogels [444] for cartilage repair, oxidized and assembled antimicrobial peptides to promote wound healing [445] and cross-linking of silk fibroin for manufacture of biodegradable wound repair membranes [446].

Treatment with the freely dissolved, recombinant bacterial TYR *Sa*MelC2 from *Streptomyces avermitilis* resulted in the formation of an injectable tissue adhesive hydrogel from tyramine-conjugated hyaluronic acid (1% w/v) and gelatin (3% w/v) for tissue engineering and regenerative medicine [447] and caused the gelation of chitosan-based hydrogels containing epigallocatechin gallate with antibacterial and immuno-modulating effects for tissue regeneration [448]. *Sa*MelC2 encapsulated in mesoporous silica nanoparticles was used for the cross-linking of poly(dimethylacrylamide) and gelatin hydrogels yielding tissue adhesives [449].

Following the same idea, also the archaeal enzyme *Nk*TYR from *Nitrosopumilus koreensis* was applied for the preparation of an injectable chitosan/gelatin hydrogel intended for tissue engineering and drug delivery [450].

## Data Availability

No datasets were generated or analyzed during the current study.

## Abbreviations

AA: l-Ascorbic acid (vitamin C)
*A. bisporus*: *Agaricus bisporus*
Å: Ångström (after Swedish physicist Anders Jonas Ångström, 1841–1874), 1 Å = 10^–^^10^ m = 0.1 nm
*Ab*MtaL: *Agaricus bisporus* Mushroom tyrosinase associated lectin-like protein
*Ab*PPOx: *Agaricus bisporus* Polyphenol oxidase x
*ab*PPOx: Gene coding for *Agaricus bisporus* polyphenol oxidase x
*Ab*T: Crude tyrosinase preparation from *Agaricus bisporus* fruiting bodies, “mushroom tyrosinase”
*Ao*CO4: *Aspergillus oryzae* Catechol oxidase 4
*Ao*MelO: *Aspergillus oryzae* Tyrosinase (*mel*O gene product)
APO: *ortho*-Aminophenol oxidase (EC 1.10.3.4)
*Bm*TYR: *Bacillus megaterium* Tyrosinase
*Bt*TYR: *Burkholderia thailandensis* Tyrosinase
*Cg*AUS1: *Coreopsis grandiflora* Aurone synthase 1
CLEA: Cross-linked enzyme aggregate
CO: Catechol oxidase (EC 1.10.3.1)
DOPA: 3,4-Dihydroxyphenylalanine = *ortho*-hydroxytyrosine
DTT: 1,4-Dithiothreitol; (2*S*,3*S*)-1,4-dimercaptobutane-2,3-diol
*E.** coli*: *Escherichia coli*
EDTA: Ethylenediaminetetraacetic acid
EPR: Electron paramagnetic resonance
GST: Glutathione S-transferase
H_2_O_2_: Hydrogen peroxide
HPG: *N*-(4-Hydroxyphenyl)-l-glutamine, γ-l-glutaminyl-4-hydroxybenzene
*Hs*TYR: *Homo sapiens* Tyrosinase
*Jr*PPO1: *Juglans regia* Polyphenol oxidase 1
kDa: Kilodalton, 1 kDa = 1000 g mol^−^^1^
LoD: Limit of detection
M: Mole per liter = 1000 mol m^−^^3^
MBP: Maltose binding protein
*Md*PPO1: *Malus domestica* Polyphenol oxidase 1
*Mj*PPOβ: *Marsupenaeus japonicus* Polyphenol oxidase β
mRNA: Messenger ribonucleic acid
NADH: Reduced nicotinamide adenine dinucleotide
NH_2_OH: Hydroxylamine
*Nk*TYR: *Nitrosopumilus koreensis* Tyrosinase
NusA: Nitrogen-utilization substance
*o*-: *Ortho*-
*Pa*PPO: *Prunus armeniaca* Polyphenol oxidase
*Pa*PvdP: *Pseudomonas aeruginosa* Pyoverdine maturation tyrosinase
*Pa*TYR1: *Polyporus arcularius* Tyrosinase 1
PEG: Polyethylene glycol
PO: Phenol oxidase
ppm: Parts per million
PPO: Polyphenol oxidase
*r*: Rate of a chemical reaction: commonly defined as the first derivative with respect to time of the product’s concentration; e.g., $A+2 B\to C:r= -\frac{d\left[c\left(A\right)\right]}{dt}=-\frac{1}{2}\frac{d\left[c\left(B\right)\right]}{dt}=\frac{d\left[c\left(C\right)\right]}{dt}$
*Rc*PPO: *Rosa chinensis* Polyphenol oxidase
*Rs*TYR: *Ralstonia solanacearum* Tyrosinase
*S. cerevisiae*: *Saccharomyces cerevisiae*
*Sc*MelC1: Caddie protein of the bacteria *Streptomyces castaneoglobisporus*
*Sc*MelC2: Tyrosinase from the bacteria *Streptomyces castaneoglobisporus*
*Sg*GriF: Gene F (a tyrosinase) in the grixazone biosynthesis gene cluster from *Streptomyces griseus*
*Sm*NspF: Gene F (a tyrosinase) in the 4-hydroxy-3-nitrosobenzamide biosynthesis gene cluster from *Streptomyces murayamaensis*
SUMO: Small ubiquitin-like modifier (a protein)
TrxA: Thioredoxin A
*Tt*PPO: *Thermothelomyces thermophilus* Polyphenol oxidase
TYR: Tyrosinase (EC 1.14.18.1)
U: Unit of enzymatic activity; commonly for PPOs: 1 U = 1 µmol of converted substrate per minute
UV: Ultraviolet
UV–Vis: Ultraviolet–visible
var.: Variety
*Vs*TYR: *Verrucomicrobium spinosum* Tyrosinase

## Footnotes

1 « Il devenait ainsi très probable que dans les sucs noircissants la tyrosine était accompagnée par **une oxydase particulière**. J’ai pu mettre celle-ci en évidence et je l’appelle ***tyrosinase***.» (emphasis added, italics in the original [11]): “It thus became very likely that the tyrosine in the blackening juices was accompanied by **a particular oxidase**. I was able to prove its existence and I name it ***tyrosinase***.”

2 The Hammett equation [151] is an empirical linear free energy relationship: log(*k*) = log(*k*_0_) + σ·ρ, where* k* is the reaction rate with a *meta*- or *para*-substituted benzene derivative,* k*_0_ is the rate of the reference reaction with the unsubstituted (i.e., H as substituent) benzene derivative, σ is the Hammett substituent constant, and ρ is the reaction constant that depends on the type of reaction and is accessible via a (Hammett) plot of log(*k*) versus σ for different substituents (same reaction, unchanged conditions).
